# Synthesis of the aggregation pheromone of *Tribolium castaneum*

**DOI:** 10.3762/bjoc.21.38

**Published:** 2025-03-06

**Authors:** Biyu An, Xueyang Wang, Ao Jiao, Qinghua Bian, Jiangchun Zhong

**Affiliations:** 1 Department of Applied Chemistry, China Agricultural University, 2 West Yuanmingyuan Road, Beijing 100193, China; 2 Shijiazhuang Camford Royal School, Shijiazhuang, Heibei, 050800, China

**Keywords:** aggregation pheromone, chiral 2-methyloxirane, red flour beetle, total synthesis

## Abstract

*Tribolium castaneum* Herbst is a destructive stored product pest. The aggregation pheromone of this pest was prepared via a new and effective strategy. The key steps include the ring-opening reaction of chiral 2-methyloxirane, the stereospecific inversion of chiral secondary tosylate, Li_2_CuCl_4_-catalyzed coupling of tosylate with Grignard reagent, and oxidation with RuCl_3_/NaIO_4._

## Introduction

The red flour beetle, *Tribolium castaneum* Herbst (Coleoptera: Tenebrionidae), is a cosmopolitan, destructive stored product pest [1], which has been found to damage 246 grain commodities, especially starchy products [2–3]. In addition, the adult *T. castaneum* secretes carcinogenic methyl-1,4-benzoquinone and ethyl-1,4-benzoquinone to inhibit the microorganisms and the predators [4–5]. Therefore, *T. castaneum* infected stored products are harmful to human health and this became a significant challenge to food security [6]. Long-term synthetic pesticide applications to control the red flour beetle has resulted in the development of resistance to organophosphates, pyrethroids, methyl carbamates, and neonicotinoids [7–8]. It became critical for devising a more effective and environmentally friendly strategy to control this pest [9].

**Figure 1:**
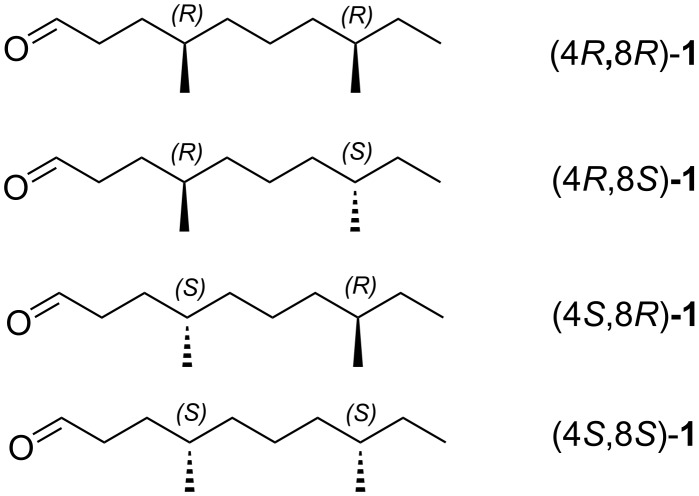
The aggregation pheromone of *Tribolium castaneum*.

Pheromone-based pest management is one of most environment benign, effective, and promising solutions [10–11]. The aggregation pheromone of *T. castaneum* was first reported by Ryan in 1976, secreted by the male, is attractive to both sexes [12]. Later, Suzuki identified the compound as 4,8-dimethyldecanal [13]. Mori synthesized four possible stereoisomers of 4,8-dimethyldecanal, and found that the response of *T. castaneum* to the (4*R*,8*R*)-isomer was identical to the natural pheromone [14–15]. In 2011, Mori and Phillips achieved the complete separation of the derivatives from the four stereoisomers by reversed-phase HPLC at −54 °C, and revealed that the natural pheromone consists of four stereoisomers of 4,8-dimethyldecanal (Figure 1) [16–17]. Previous syntheses mainly focused on chiral sources of (*R*)-citronellic acid [18], methyl (*S*)-3-hydroxy-2-methylpropanoate, (*S*)-2-methyl-1-butanol [19], (*R*)-2,3-*O*-isopropylideneglyceraldehyde [20], (*R*)- and (*S*)-citronellol [21], (*R*)-4-methyl-δ-valerolactone [22], porcine pancreatic lipase (PPL)-catalyzed acetylation of racemic citronellol [23], and Evan′s inductive methylation [24].

To research further the bioactivity of the pheromone, herein, we report an effective synthesis of the aggregation pheromone of *T. castaneum*, which uses the cheap (*R*)- and (*S*)-2-methyloxirane as chiral sources, connects two chiral building blocks through Li_2_CuCl_4_-catalyzed coupling, and finally leads to the target pheromones by olefin oxidation with RuCl_3_/NaIO_4_.

## Results and Discussion

**Scheme 1:**
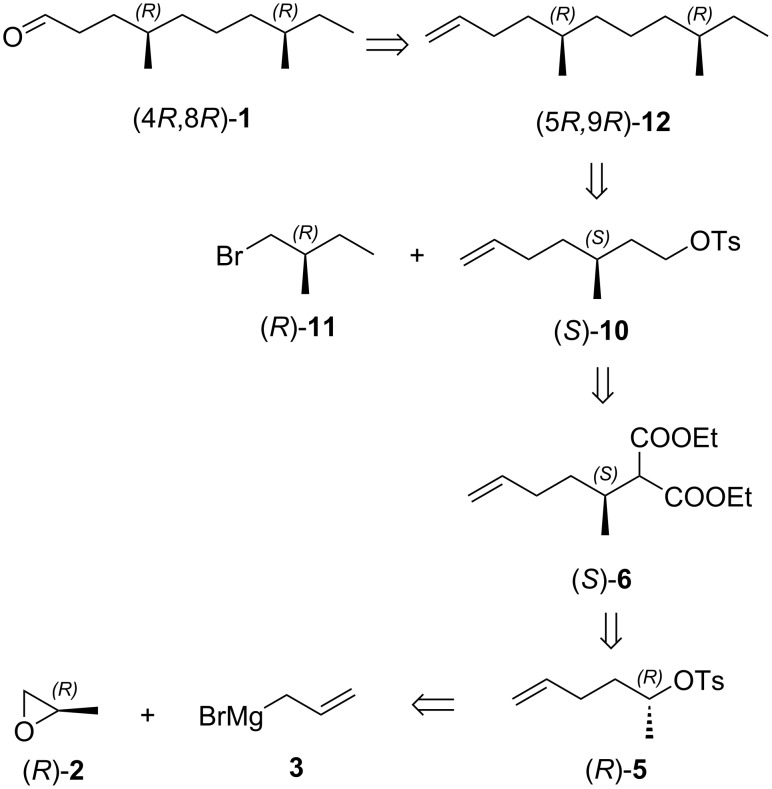
Retrosynthetic analysis of the aggregation pheromone (4*R*,8*R*)-**1**.

The retrosynthetic analysis of the aggregation pheromone (4*R*,8*R*)-**1** is shown in Scheme 1. Obviously, the target pheromone (4*R*,8*R*)-**1** could be synthesized via an oxidation of chiral terminal olefine (5*R*,9*R*)-**12**, which could be obtained through Li_2_CuCl_4_-catalyzed coupling of chiral tosylate (*S*)-**10** with a Grignard reagent derived from (*R*)-1-bromo-2-methylbutane ((*R*)-**11**). The key chiral building block (*S*)-**10** was envisaged to be prepared through a sequence of hydrolyzation, decarboxylation, borane-amine reduction and tosylation from diethyl (*S*)-2-(hex-5-en-2-yl)malonate ((*S*)-**6**). The stereocenter in geminal ester (*S*)-**6** could be derived from (*R*)-2-methyloxirane ((*R*)-**2**) via a ring-opening reaction and a stereospecific inversion of the chiral secondary tosylate (*R*)-**5**. Following the similar procedure for (4*R*,8*R*)-**1**, the other constituents of the aggregation pheromone (4*R*,8*S*)-**1**, (4*S*,8*R*)-**1** and (4*S*,8*S*)-**1** could be prepared.

**Scheme 2:**
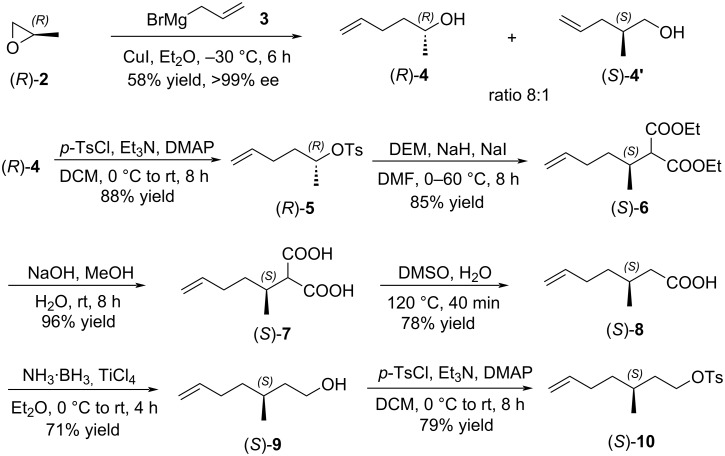
Synthesis of chiral tosylate (*S*)-**10**.

Based on the retrosynthetic analysis of the aggregation pheromone (4*R*,8*R*)-**1**, our synthesis began with the preparation of chiral tosylate (*S*)-**10** (Scheme 2). The ring-opening reaction of (*R*)-2-methyloxirane ((*R*)-**2**) with allylmagnesium bromide (**3**) catalyzed by CuI produced a mixture of (*R*)-hex-5-en-2-ol ((*R*)-**4**) and (*S*)-2-methylpent-4-en-1-ol ((*S*)-**4’**) (ratio 8:1, determined by ^1^H NMR spectroscopy) [25–26]. The primary alcohol (*S*)-**4’** could be easily removed by a selective TEMPO oxidation. The optical purity of the chiral secondary alcohol (*R*)-**4** was more than 99% ee, determined by ^1^H NMR spectrum of its Mosher ester [27–28]. The subsequent tosylation with *p*-tosyl chloride gave (*R*)-hex-5-en-2-yl 4-methylbenzenesulfonate ((*R*)-**5**) in 88% yield [29]. The reaction of (*R*)-**5** with the enolate of diethyl malonate yielded (*S*)-2-(hex-5-en-2-yl)malonate ((*S*)-**6**), and realized a stereospecific inversion of chiral secondary tosylate (*R*)-**5** [30–31]. The geminal ester (*S*)-**6** was next treated with NaOH in methanol to afford (*S*)-2-(hex-5-en-2-yl)malonic acid ((*S*)-**7**) in 96% yield [32]. Then, geminal acid (*S*)-**7** was decarboxylated with DMSO to yield chiral acid (*S*)-**8** [33], followed by TiCl_4_-catalyzed reduction with ammonia-borane to obtain the chiral alkenyl alcohol (*S*)-**9** [34]. The final tosylation with *p*-tosyl chloride provided (*S*)-3-methylhept-6-en-1-yl 4-methylbenzenesulfonate ((*S*)-**10**) [29].

**Scheme 3:**
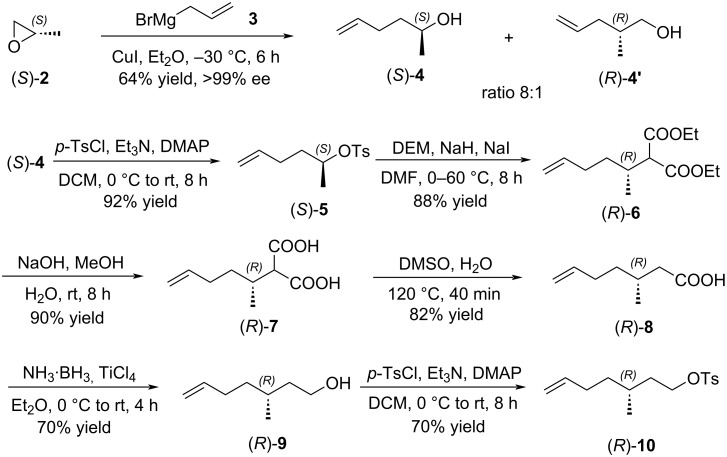
Synthesis of chiral tosylate (*R*)-**10**.

Similarly, chiral tosylate (*R*)-**10** could be prepared from (*S*)-2-methyloxirane ((*S*)-**2**) through the ring-opening reaction, tosylation, stereospecific inversion, hydrolysis, decarboxylation, reduction, and second tosylation (Scheme 3).

**Scheme 4:**
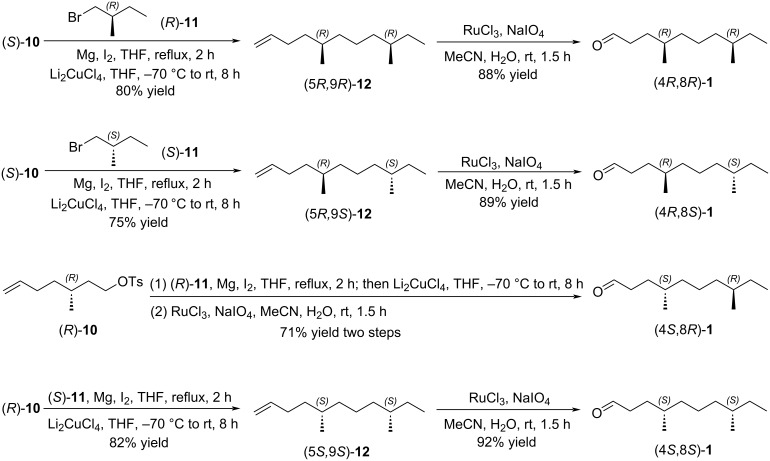
Synthesis of the aggregation pheromone of *Tribolium castaneum*.

With two the chiral building blocks (*R*)-**10** and (*S*)-**10** in hand, we next prepared the target aggregation pheromone (4*R*,8*R*)-**1**, (4*R*,8*S*)-**1**, (4*S*,8*R*)-**1**, and (4*S*,8*S*)-**1** (Scheme 4). Li_2_CuCl_4_-catalyzed coupling of chiral tosylate (*S*)-**10** with the Grignard reagent derived from (*R*)-1-bromo-2-methylbutane ((*R*)-**11**) and Mg afforded (5*R*,9*R*)-5,9-dimethylundec-1-ene ((5*R*,9*R*)-**12**) in 80% yield [35]. (4*R*,8*R*)-4,8-Dimethyldecanal ((4*R*,8*R*)-**1**) was obtained from chiral terminal olefine (5*R*,9*R*)-**12** through the oxidation with RuCl_3_ and NaIO_4_ [36], and its specific rotation and NMR spectrum matched with the reference [20]. Moreover, using the similar procedure for (4*R*,8*R*)-**1**, the other three constituents of the aggregation pheromone (4*R*,8*S*)-**1**, (4*S*,8*R*)-**1**, and (4*S*,8*S*)-**1** were prepared through Li_2_CuCl_4_-catalyzed coupling and oxidation with RuCl_3_/NaIO_4_ from chiral building blocks (*R*)-**10**, (*S*)-**10**, (*R*)-**11** and (*S*)-**11**, which were characterized by NMR spectroscopy and HRMS.

## Conclusion

In summary, we have achieved a novel and effective synthesis of the aggregation pheromone of *T. castaneum*, (4*R*,8*R*)-, (4*R*,8*S*)-, (4*S*,8*R*)- and (4*S*,8*S*)-4,8-dimethyldecanal. In our strategy, (*S*)- and (*R*)-2-methyloxirane acted as chiral sources, whereas a Li_2_CuCl_4_-catalyzed coupling was used to connect two key building blocks, a chiral tosylate and a chiral Grignard reagent. The synthetic pheromone could be valuable for the control of the red flour beetle.

## Supporting Information

File 1 General information, synthesis of compounds **1**–**12**, research on the optical purity of chiral alcohols (*R*)- and (*S*)-**4**, and copies of ^1^H, ^13^C and ^19^F NMR spectra.

## Data Availability

All data that supports the findings of this study is available in the published article and/or the supporting information of this article.
