# Supplementary material for: Synthesis of the aggregation pheromone of Tribolium castaneum
Source: Beilstein J Org Chem. 2025 Mar 6;21:510–4. doi: 10.3762/bjoc.21.38 (PMC11897649; doi:10.3762/bjoc.21.38)
Supplement: File 1 — General information, synthesis of compounds 1–12, research on the optical purity of chiral alcohols (R)- and (S)-4, and copies of 1H, 13C and 19F NMR spectra. [file Beilstein_J_Org_Chem-21-510-s001.pdf]

BEILSTEIN JOURNAL OF ORGANIC CHEMISTRY

Supporting Information

for

Synthesis of the aggregation pheromone of *Tribolium castaneum*

Biyu An, Xueyang Wang, Ao Jiao, Qinghua Bian and Jiangchun Zhong

Beilstein J. Org. Chem. **2025,** *21,* 510–514. doi:10.3762/bjoc.21.38

General information, synthesis of compounds 1–12, research on the optical purity of chiral alcohols (*R*)- and (*S*)-4, and copies of ^{1}H, ^{13}C and ^{19}F NMR spectra

License and Terms: This is a supporting information file under the terms of the Creative Commons Attribution License (https://creativecommons.org/licenses/by/4.0). Please note that the reuse, redistribution and reproduction in particular requires that the author(s) and source are credited and that individual graphics may be subject to special legal provisions.
The license is subject to the *Beilstein Journal of Organic Chemistry* terms and conditions: (https://www.beilstein-journals.org/bjoc/terms)

Table of contents

1. General information S2

2. Synthesis of the aggregation pheromone of *Tribolium castaneum* S2

3. Research on the optical purity of chiral alcohols (*S*)- and (*R*)-**4** S17

4. ^{1}H,^{13}C and ^{19}F NMR spectra of the products S20

5. References S46

1. General information

Unless otherwise indicated, all reactions were conducted in dry glassware with a Schlenk line under an argon atmosphere. The commercial reagents of CuI, *p*-TsCl, DMAP, NaH, DEM, $NH_3{\cdot}BH_3$, $RuCl_3$, and (*S*)-MTPACl were analytical reagent grade and used as received. The solvents of Et_2O, DCM, THF, and Et_3N were dried from CaH_2 and distilled prior to use. Optical rotations were measured on a Rudolph AUTOPOL-IV polarimeter. ^{1}H and ^{13}C NMR spectra were collected on a Bruker AscendTM 500 MHz spectrometer, and the chemical shifts were reported in ppm with the references of TMS (0.00 ppm) and $CDCl_3$ (77.16 ppm). High-resolution mass (HRMS) data were obtained from a Waters LCT Premier™ mass spectrometer equipped with an ESI source.

2. Synthesis of the aggregation pheromone of *Tribolium castaneum*

Scheme S1: Synthesis of chiral tosylate (*S*)-**10**.

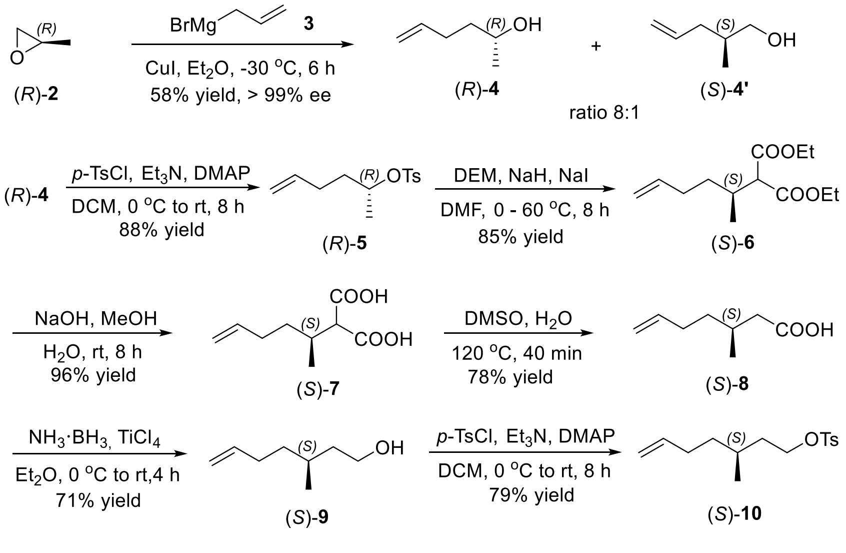

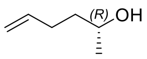 (R)-Hex-5-en-2-ol ((R)-**4**) (CAS 17397-29-4) [1,2]

To a 500 mL Schlenk flask was added CuI (0.98 g, 5.17 mmol) at room temperature. The flask was cooled to −30 °C, and then allylmagnesium bromide (**3**, 77.5 mL, 1.0 M in THF, 77.50 mmol) was added at the same temperature. After being stirred for 30 min, (R)-2-methyloxirane ((R)-**2**, 3.00 g, 51.65 mmol) in dry Et_2O (30.0 mL) was added dropwise through a syringe over 2 h. The reaction mixture was maintained for 6 h at −30 °C, followed by quenching with saturated NH_4Cl aqueous solution (50 mL). After allowing the mixture to warm to room temperature, it was diluted with water (50 mL). The two layers were separated, and the aqueous layer was extracted with Et_2O (50 mL × 3). The ether extracts were combined with the organic layer, and washed with brine (280 mL). The solution was dried over anhydrous Na_2SO_4, and concentrated by a rotary evaporator at low temperature. The residue was a yellow liquid, which was a mixture of (R)-hex-5-en-2-ol ((R)-**4**) and (S)-2-methylpent-4-en-1-ol ((S)-**4'**) (4.83 g, ratio 8:1, determined by the ^{1}H NMR spectrum).

In order to remove (S)-2-methylpent-4-en-1-ol ((S)-**4'**) from the chiral secondary alcohol (R)-**4**, a selective oxidation of the primary alcohol with TEMPO was conducted. To another 200 mL Schlenk flask were added TEMPO (0.17 g, 1.06 mmol) and TBACl (0.29 g, 1.06 mmol) at room temperature, and an aqueous buffer solution (55 mL, 0.5 M $NaHCO_3$, and 0.05 M K_2CO_3) and DCM (55 mL) were added through a syringe. Subsequently, the mixture of (R)-hex-5-en-2-ol ((R)-**4**) and (S)-2-methylpent-4-en-1-ol ((S)-**4'**) (4.83 g) and NCS (1.84 g, 13.78 mmol) were added. The reaction mixture was maintained for 8 h at room temperature, followed by quenching with H_2O (50 mL). The two layers were separated, and the aqueous layer was extracted with DCM (50 mL × 3). The dichloromethane extracts were combined with the organic layer, and washed with brine (230 mL). The solution was dried over anhydrous Na_2SO_4, and concentrated by a rotary evaporator at low temperature. The residue was

purified by column chromatography on silica gel with an eluent of *n*-pentane/Et_2O 3:2 to afford (*R*)-hex-5-en-2-ol ((*R*)-**4**, 2.83 g, 58% yield, >99% ee, determined by ^{1}H NMR spectroscopy of its Mosher ester) as a colorless oil. $[\alpha]_D^{22}$ = –7.38 (c = 2.17, $CHCl_3$). ^{1}H NMR (500 MHz, $CDCl_3$) δ 5.84 (ddt, *J* = 16.9, 10.2, 6.7 Hz, 1H), 5.05 (dq, *J* = 17.2, 1.7 Hz, 1H), 4.98 (dq, *J* = 10.1, 1.5 Hz, 1H), 3.85 – 3.81 (m, 1H), 2.21 – 2.11 (m, 2H), 1.61 – 1.52 (m, 2H), 1.39 (br s, 1H), 1.21 (d, *J* = 6.2 Hz, 3H). ^{13}C NMR (126 MHz, $CDCl_3$) δ 138.6, 114.9, 67.9, 38.4, 30.3, 23.6. HRMS (ESI) m/z: calcd for $C_6H_{12}O$ $[M]^+$ 100.08827, found 100.08735.

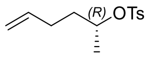

(*R*)-Hex-5-en-2-yl 4-methylbenzenesulfonate ((*R*)-**5**) (CAS 2715086-71-6) [3]

To a 200 mL Schlenk flask were added *p*-TsCl (5.93 g, 31.10 mmol), DMAP (1.74 g, 14.20 mmol), and DCM (100 mL) at room temperature. After the resulting mixture was cooled to 0 °C and stirred for 30 min, Et_3N (5.75 g, 56.80 mmol) and (*R*)-hex-5-en-2-ol ((*R*)-**4**, 2.83 g, 28.27 mmol) were added sequentially. After allowing the reaction solution to warm to room temperature and maintain for 8 h, the reaction was quenched with saturated $NaHCO_3$ aqueous solution (50 mL). The two layers were separated, and the aqueous layer was extracted with DCM (50 mL × 3). The dichloromethane extracts were combined with the organic layer, washed with brine (210 mL). The solution was dried over anhydrous Na_2SO_4 and concentrated by a rotary evaporator. The residue was purified by column chromatography on silica gel with an eluent of petroleum ether/ethyl acetate 20:1 to afford (*R*)-hex-5-en-2-yl 4-methylbenzenesulfonate ((*R*)-**5**, 6.33 g, 88% yield) as a pale yellow oil. $[\alpha]_D^{22}$ = –1.75 (c = 3.43, $CHCl_3$). ^{1}H NMR (500 MHz, $CDCl_3$) δ 7.80 (d, *J* = 8.1 Hz, 2H), 7.34 (d, *J* = 8.0 Hz, 2H), 5.69 – 5.65 (m, 1H), 4.94 (dq, *J* = 13.7, 1.8 Hz, 2H), 4.65 – 4.62 (m, 1H), 2.45 (s, 3H), 2.05 – 1.96 (m, 2H), 1.75 – 1.70 (m, 1H), 1.62

– 1.56 (m, 1H), 1.27 (d, *J* = 6.4 Hz, 3H). ^{13}C NMR (126 MHz, $CDCl_3$) δ 144.6, 137.2, 134.7, 129.9, 127.9, 115.5, 80.0, 35.8, 29.2, 21.8, 20.9. HRMS (ESI) m/z: calcd for $C_{13}H_{18}O_3SNa$ [M+Na]$^+$ 277.0869, found 277.0850.

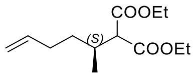

Diethyl (*S*)-2-(hex-5-en-2-yl)malonate ((*S*)-**6**) (new compound) [4,5]

To a 200 mL Schlenk flask was added NaH (0.65 g, 60% in mineral oil, 16.14 mmol) at room temperature. The flask was cooled to 0 °C, then diethyl malonate (4.39 g, 27.40 mmol) in dry DMF (125 mL) was added. After the resulting mixture was warmed to room temperature and stirred for 30 min, it was cooled to 0 °C. Subsequently, (*R*)-hex-5-en-2-yl 4-methylbenzenesulfonate ((*R*)-**5**, 3.73 g, 14.67 mmol) and NaI (2.20 g, 14.67 mmol) were added. The reaction mixture was heated to 60 °C and stirred for 8 h, followed by quenching with water (50 mL) at 0 °C. The two layers were separated, and the aqueous layer was extracted with Et_2O (50 mL × 3). The ether extracts were combined with the organic layer, and washed with saturated NH_4Cl aqueous solution (200 mL) and brine (200 mL). The solution was dried over anhydrous Na_2SO_4, and concentrated by rotary evaporation. The residue was purified by column chromatography on silica gel with an eluent of petroleum ether/ethyl acetate 20:1 to afford diethyl (*S*)-2-(hex-5-en-2-yl)malonate ((*S*)-**6**, 3.02 g, 85% yield) as a colorless oil. ^{1}H NMR (500 MHz, $CDCl_3$) δ 5.71 (ddt, *J* = 16.8, 10.1, 6.6 Hz, 1H), 4.96 (dq, *J* = 17.1, 1.8 Hz, 1H), 4.88 (dq, *J* = 10.2, 1.5 Hz, 1H), 4.12 (q, *J* = 7.1 Hz, 4H), 3.17 (d, *J* = 7.9 Hz, 1H), 2.22 – 2.19 (m, 1H), 2.11 – 2.06 (m, 1H), 1.99 – 1.96 (m, 1H), 1.49 – 1.45 (m, 1H), 1.27 – 1.23 (m, 1H), 1.22 – 1.18 (m, 6H), 0.92 (d, *J* = 6.7 Hz, 3H). ^{13}C NMR (126 MHz, $CDCl_3$) δ 169.1, 168.9, 138.4, 114.9, 61.3, 61.2, 57.8, 33.6, 33.0, 31.2, 16.9, 14.3, 14.2. HRMS (ESI) m/z: calcd for $C_{13}H_{23}O_4$ [M+H]$^+$ 243.15909, found 243.15937.

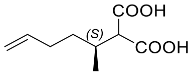 (S)-2-(Hex-5-en-2-yl)malonic acid ((S)-**7**) (new compound) [6]

To a 100 mL Schlenk flask was added diethyl (S)-2-(hex-5-en-2-yl)malonate ((S)-**6**, 2.09 g, 8.63 mmol) in methanol (13.5 mL) at room temperature. Then, NaOH (1.15 g, 28.77 mmol) in water (13.5 mL) was added. The reaction solution was maintained at room temperature for 8 h, followed by an acidification to pH 2 with aqueous hydrochloric acid solution (1.0 M). The two layers were separated, and the aqueous layer was extracted with EtOAc (15 mL × 3). The ester extracts were combined with the organic layer and washed with brine (30 mL). The solution was dried over anhydrous Na_2SO_4 and concentrated by rotary evaporation. The residue was a white solid, which was (S)-2-(hex-5-en-2-yl)malonic acid ((S)-**7**, 1.54 g, 96% yield). ^{1}H NMR (500 MHz, CD_3SOCD_3) δ 12.64 (br s, 2H), 5.81 – 5.73 (m, 1H), 5.04 – 4.99 (m, 1H), 4.96 – 4.93 (m, 1H), 3.08 (d, J = 8.1 Hz, 1H), 2.11 – 1.94 (m, 3H), 1.51 – 1.45 (m, 1H), 1.27 – 1.22 (m, 1H), 0.92 (d, J = 6.7 Hz, 3H). ^{13}C NMR (126 MHz, CD_3SOCD_3) δ 170.3, 170.2, 138.5, 114.9, 57.5, 32.9, 31.9, 30.6, 16.6. HRMS (ESI) m/z: calcd for $C_9H_{16}O_5$ $[M+H_2O]^+$ 204.09923, found 204.09829.

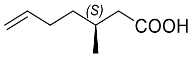 (S)-3-Methylhept-6-enoic acid ((S)-**8**) (CAS 116370-22-0) [7]

To a 100 mL four-necked flask were added DMSO (28 mL) and H_2O (0.1 mL) at room temperature. After the mixture was heated to 120 °C and stirred for 10 min, (S)-2-(hex-5-en-2-yl)malonic acid ((S)-**7**, 1.84 g, 9.88 mmol) in DMSO (19 mL) was added through a syringe. The reaction mixture was maintained for 40 min, followed by quenching with water (10 mL) at room temperature. The two layers were separated, and the aqueous layer was extracted with EtOAc (20 mL × 3). The ester extracts were combined with the organic layer and washed with brine (30 mL). The solution was dried over anhydrous Na_2SO_4 and concentrated by rotary evaporation. The residue was purified by column

chromatography on silica gel with an eluent of dichloromethane/methanol 20:1 to afford (*S*)-2-(hex-5-en-2-yl) malonic acid ((*S*)-**8**, 1.10 g, 78% yield) as a pale yellow liquid. $[\alpha]_D^{22}$ = –1.83 (c = 1.09, $CHCl_3$). ^{1}H NMR (500 MHz, CD_3SOCD_3) δ 12.00 (br s, 1H), 5.82 – 5.74 (m, 1H), 5.03 – 4.99 (m, 1H), 4.95 – 4.92 (m, 1H), 2.24 – 2.20 (m, 1H), 2.06 – 1.96 (m, 3H), 1.87 – 1.80 (m, 1H), 1.40 – 1.35 (m, 1H), 1.26 – 1.20 (m, 1H), 0.88 (d, *J* = 6.6 Hz, 3H). ^{13}C NMR (126 MHz, CD_3SOCD_3) δ 173.9, 138.7, 114.7, 41.2, 35.1, 30.7, 29.2, 19.4. HRMS (ESI) m/z: calcd for $C_8H_{16}O_3$ $[M+H_2O]^+$ 160.10940, found 160.11054.

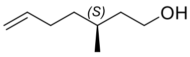

(*S*)-3-Methylhept-6-en-1-ol ((*S*)-**9**) (CAS 84717-45-3) [8a]

To a 100 mL four-necked flask was added (*S*)-3-methylhept-6-enoic acid ((*S*)-**8**, 0.91 g, 6.40 mmol) in Et_2O (20 mL) at room temperature, and $TiCl_4$ (0.65 mL, 1.0 M in CH_2Cl_2, 0.65 mmol) was added dropwise. After being cooled to 0 °C, $NH_3 \cdot BH_3$ (0.40 g, 12.80 mmol) was added in portions and stirred for 10 min. After allowing the reaction mixture to warm to room temperature and stir for 4 h, it was acidified to pH 2 with aqueous hydrochloric acid solution (1 M) at 0 °C. The two layers were separated, and the aqueous layer was extracted with Et_2O (20 mL × 3). The ether extracts were combined with the organic layer, and washed with brine (30 mL). The solution was dried over anhydrous Na_2SO_4 and concentrated by rotary evaporation at low temperature. The residue was purified by column chromatography on silica gel with an eluent of *n*-pentane/Et_2O 3:2 to afford (*S*)-3-methylhept-6-en-1-ol ((*S*)-**9**, 0.58 g, 71% yield) as a pale yellow liquid. $[\alpha]_D^{22}$ = –2.50 (c = 1.28, $CHCl_3$). ^{1}H NMR (500 MHz, $CDCl_3$) δ 5.85 – 5.77 (m, 1H), 5.02 (dd, *J* = 17.1, 1.8 Hz, 1H), 4.93 (dd, *J* = 10.1, 1.9 Hz, 1H), 3.73 – 3.64 (m, 2H), 2.13 – 2.01 (m, 3H), 1.63 – 1.57 (m, 2H), 1.45 – 1.38 (m, 2H), 1.24 – 1.20 (m, 1H), 0.91 (d, *J* = 6.5 Hz, 3H). ^{13}C NMR (126 MHz, $CDCl_3$) δ 139.2, 114.4, 61.2, 39.9, 36.4, 31.4, 29.1, 19.6. HRMS (ESI) m/z: calcd for $C_8H_{16}ONa$ $[M+Na]^+$ 151.10934, found 151.10876.

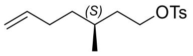

(*S*)-3-Methylhept-6-en-1-yl 4-methylbenzenesulfonate ((*S*)-**10**) (CAS 1147880-47-4)

Prepared in a similar manner as described for chiral tosylate (*S*)-**5**, the tosylation of (*S*)-3-methylhept-6-en-1-ol ((*S*)-**9**, 0.55 g, 4.29 mmol) with *p*-TsCl (0.90 g, 4.72 mmol) afforded (*S*)-3-methylhept-6-en-1-yl 4-methylbenzene-sulfonate ((*S*)-**10**, 0.96 g, 79% yield) as a pale yellow oil. ^{1}H NMR (500 MHz, $CDCl_3$) δ 7.79 (d, *J* = 8.3 Hz, 2H), 7.35 (d, *J* = 8.2 Hz, 2H), 5.76 – 5.71 (m, 1H), 4.99 – 4.94 (m, 1H), 4.93 – 4.91 (m, 1H), 4.09 – 4.04 (m, 2H), 2.45 (s, 3H), 2.04 – 1.94 (m, 2H), 1.70 – 1.65 (m, 1H), 1.55 – 1.53 (m, 1H), 1.48 – 1.42 (m, 1H), 1.32 – 1.28 (m, 1H), 1.20 – 1.16 (m, 1H), 0.82 (d, *J* = 6.6 Hz, 3H). ^{13}C NMR (126 MHz, $CDCl_3$) δ 144.8, 138.8, 133.3, 129.9, 128.0, 114.6, 69.1, 35.9, 35.7, 31.1, 28.8, 21.8, 19.1. The ^{1}H NMR data for this compound is consistent with the literature data [8b].

Scheme S2: Synthesis of chiral tosylate (*R*)-**10**.

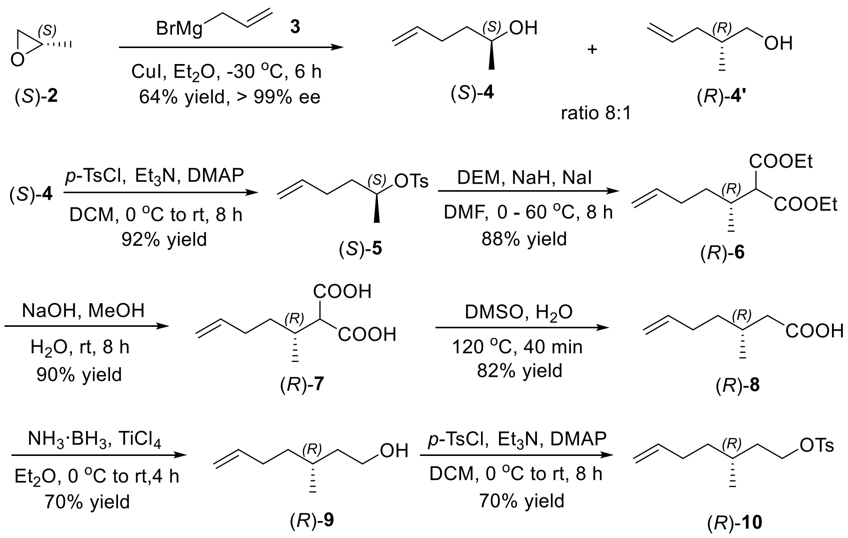

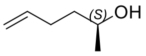 (*S*)-Hex-5-en-2-ol ((*S*)-**4**) (CAS 17397-24-9) [1,2]

In a similar manner as described for chiral alcohol (*R*)-**4**, the ring-opening reaction of (*S*)-2-methyloxirane ((*S*)-**2**, 3.30 g, 56.82 mmol) with allylmagnesium bromide (85.3 mL, 1.0 M THF, 85.3 mmol) catalyzed by CuI (1.08 g, 5.69 mmol) afforded (*S*)-hex-5-en-2-ol ((*S*)-**4**, 3.64 g, 64% yield, >99% ee, determined by ^{1}H NMR of its Mosher ester) as a colorless oil. $[\alpha]_D^{22}$ = +4.43 (c = 5.51, $CHCl_3$). ^{1}H NMR (500 MHz, $CDCl_3$) δ 5.84 (ddt, *J* = 16.9, 10.2, 6.7 Hz, 1H), 5.08 – 4.96 (m, 2H), 3.84 – 3.81 (m, 1H), 2.19 – 2.12 (m, 2H), 1.58 – 1.54 (m, 2H), 1.36 (br s, 1H), 1.21 (d, *J* = 6.2 Hz, 3H). ^{13}C NMR (126 MHz, $CDCl_3$) δ 138.6, 114.9, 67.9, 38.4, 30.3, 23.6. HRMS (ESI) m/z: calcd for $C_6H_{12}ONa$ $[M+Na]^+$ 123.07804, found 123.0788.

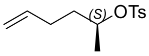 (*S*)-Hex-5-en-2-yl 4-methylbenzenesulfonate ((*S*)-**5**) (CAS 2715086-72-7) [3]

In a similar manner as described for chiral tosylate (*R*)-**5**, the tosylation of (*S*)-hex-5-en-2-ol ((*S*)-**4**, 4.26 g, 42.54 mmol) with *p*-TsCl (8.89 g, 46.80 mmol) afforded (*S*)-hex-5-en-2-yl 4-methylbenzenesulfonate ((*S*)-**5**, 9.92 g, 92% yield) as a colorless oil. $[\alpha]_D^{22}$ = +4.94 (c = 3.65, $CHCl_3$). ^{1}H NMR (500 MHz, $CDCl_3$) δ 7.81 – 7.79 (m, 2H), 7.34 (d, *J* = 8.1 Hz, 2H), 5.70 – 5.65 (m, 1H), 4.96 – 4.91 (m, 2H), 4.66 – 4.62(m, 1H), 2.45 (s, 3H), 2.05 – 1.96 (m, 2H), 1.76 – 1.69 (m, 1H), 1.62 – 1.55 (m, 2H), 1.27 (d, *J* = 6.3 Hz, 3H). ^{13}C NMR (126 MHz, $CDCl_3$) δ 144.6, 137.2, 134.7, 129.9, 127.9, 115.5, 80.0, 35.8, 29.2, 21.8, 20.9. HRMS (ESI) m/z: calcd for $C_{13}H_{19}O_3S$ $[M+H]^+$ 255.10494, found 255.10446.

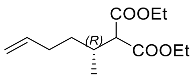 Diethyl (*R*)-2-(hex-5-en-2-yl)malonate ((*R*)-**6**) (new compound) [4,5]

In a similar manner as described for alkenyl malonate (*S*)-**6**, the reaction of (*S*)-hex-5-en-2-yl 4-methylbenzenesulfonate ((*S*)-**5**, 3.73 g, 14.67 mmol) with diethyl malonate (4.39 g, 27.40 mmol) afforded diethyl (*R*)-2-(hex-5-en-2-yl) malonate ((*R*)-**6**, 3.13 g, 88% yield) as a colorless oil. ^{1}H NMR (500 MHz, $CDCl_3$) δ 5.78 (ddt, *J* = 16.9, 10.2, 6.6 Hz, 1H), 5.04 – 4.94 (m, 2H), 4.20 (q, *J* = 7.1 Hz, 4H), 3.24 (d, *J* = 7.9 Hz, 1H), 2.29 – 2.27 (m, 1H), 2.22 – 2.15 (m, 1H), 2.06 – 2.02 (m, 1H), 1.57 – 1.52 (m, 1H), 1.32 – 1.29 (m, 1H), 1.27 (td, *J* = 7.2, 1.4 Hz, 6H), 1.00 (d, *J* = 6.8 Hz, 3H). ^{13}C NMR (126 MHz, $CDCl_3$) δ 169.1, 168.9, 138.4, 114.9, 61.3, 61.2, 57.8, 33.6, 33.0, 31.2, 16.9, 14.3, 14.2. HRMS (ESI) m/z: calcd for $C_{13}H_{23}O_4$ $[M+H]^+$ 243.15909, found 243.16007.

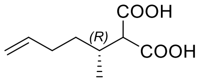 (*R*)-2-(Hex-5-en-2-yl)malonic acid ((*R*)-**7**) (new compound) [6]

In a similar manner as described for alkenyl malonic acid (*S*)-**7**, the hydrolysis of diethyl (*R*)-2-(hex-5-en-2-yl)malonate ((*R*)-**6**, 1.97 g, 8.13 mmol) with NaOH (1.08 g, 27.10 mmol) afforded diethyl (*R*)-2-(hex-5-en-2-yl) malonic acid ((*R*)-**7**, 1.36 g, 90% yield). ^{1}H NMR (500 MHz, $CDCl_3$) δ 11.07 (br s, 2H) 5.91 (ddt, *J* = 16.9, 10.2, 6.6 Hz, 1H), 5.16 (dd, *J* = 17.1, 1.8 Hz, 1H), 5.10 (dd, *J* = 10.2, 1.8 Hz, 1H), 3.52 (d, *J* = 7.2 Hz, 1H), 2.46 – 2.40 (m, 1H), 2.33 – 2.21 (m, 1H), 2.20 – 2.15 (m, 1H), 1.78 – 1.71 (m, 1H), 1.56 – 1.48 (m, 1H), 1.20 (d, *J* = 6.8 Hz, 3H). ^{13}C NMR (126 MHz, $CDCl_3$) δ 173.9, 173.6, 137.9, 115.3, 56.7, 33.5, 33.4, 31.2, 16.8. HRMS (ESI) m/z: calcd for $C_9H_{16}O_5$ $[M+H_2O]^+$ 204.09923, found 204.09978.

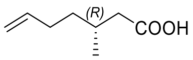

(*R*)-3-Methylhept-6-enoic acid ((*R*)-**8**) (CAS 161029-67-0) [7]

In a similar manner as described for alkenyl acid (*S*)-**8**, the decarboxylation of (*R*)-2-(hex-5-en-2-yl)malonic acid ((*R*)-**7**, 1.35 g, 7.25 mmol) with DMSO (35 mL) and H_2O (0.1 mL) afforded (*R*)-3-methylhept-6-enoic acid ((*R*)-**8**, 0.85 g, 82% yield) as a colorless oil. $[\alpha]_D^{22}$ =+0.20 (c = 2.05, $CHCl_3$). ^{1}H NMR (500 MHz, $CDCl_3$) δ 11.70 (s, 1H), 5.80 (ddt, *J* = 16.9, 10.2, 6.6 Hz, 1H), 5.78 (dd, *J* = 16.9, 1.6 Hz, 1H), 4.96 (dd, *J* = 10.2, 1.6 Hz, 1H), 2.37 (dd, *J* = 15.1, 6.0 Hz, 1H), 2.17 (dd, *J* = 15.1, 8.1 Hz, 1H), 2.15 – 2.06 (m, 2H), 2.04 – 1.96 (m, 1H), 1.45 – 1.43 (m, 1H), 1.35 – 1.28 (m, 1H), 0.99 (d, *J* = 6.7 Hz, 3H). ^{13}C NMR (126 MHz, $CDCl_3$) δ 180.0, 138.6, 114.8, 41.6, 35.9, 31.3, 29.8, 19.6. HRMS (ESI) m/z: calcd for $C_8H_{15}O_2$ $[M+H]^+$ 143.10666, found 143.1060.

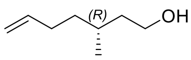

(*R*)-3-Methylhept-6-en-1-ol ((*R*)-**9**) (CAS 237431-14-0) [8]

In a similar manner as described for alkenyl alcohol (*S*)-**9**, the reduction of (*R*)-3-methylhept-6-enoic acid ((*R*)-**8**, 0.76 g, 5.34 mmol) with $NH_3{\cdot}BH_3$ (0.33 g, 10.68 mmol) afforded (*R*)-3-methylhept-6-en-1-ol ((*R*)-**9**, 0.48 g, 70% yield) as a pale yellow oil. $[\alpha]_D^{22}$ = +0.38 (c = 2.13, $CHCl_3$). ^{1}H NMR (500 MHz, $CDCl_3$) δ 5.76 – 5.69 (m, 1H), 4.93 (dd, *J* = 17.1, 1.7 Hz, 1H), 4.86 (dd, *J* = 10.3, 1.3 Hz, 1H), 3.62 – 3.56 (m, 2H), 2.04 – 1.95 (m, 3H), 1.56 – 1.50 (m, 2H), 1.36 – 1.30 (m, 2H), 1.19 – 1.14 (m, 1H), 0.83 (d, *J* = 6.5 Hz, 3H). ^{13}C NMR (126 MHz, $CDCl_3$) δ 139.2, 114.3, 61.0, 39.9, 36.3, 31.3, 29.1, 19.5. HRMS (ESI) m/z: calcd for $C_8H_{16}ONa$ $[M+Na]^+$ 151.10934, found 151.10875.

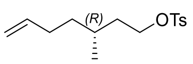

(*R*)-3-Methylhept-6-en-1-yl 4-methylbenzenesulfonate ((*R*)-**10**) (CAS 1147880-36-1)

In a similar manner as described for chiral tosylate (*S*)-**5**, the tosylation of (*R*)-3-methylhept-6-en-1-ol ((*R*)-**9**, 0.39 g, 3.04 mmol) with *p*-TsCl (0.64 g, 3.34 mmol) afforded (*R*)-3-methylhept-6-en-1-yl 4-methylbenzenesulfonate ((*R*)-**10**, 0.60 g, 70% yield) as a pale yellow oil. ^{1}H NMR (500 MHz, $CDCl_3$) δ 7.79 (d, *J* = 8.1 Hz, 2H), 7.35 (d, *J* = 8.2 Hz, 2H), 5.73 (ddt, *J* = 16.9, 10.2, 6.6 Hz, 1H), 4.96 (dd, *J* = 17.1, 1.8 Hz, 1H), 4.93 (dd, *J* = 10.2, 1.7 Hz, 1H), 4.09 – 4.03 (m, 2H), 2.45 (s, 3H), 2.04 – 1.94 (m, 2H), 1.71 – 1.65 (m, 1H), 1.57 – 1.53 (m, 1H), 1.48 – 1.41 (m, 1H), 1.35 – 1.26 (m, 1H), 1.19 – 1.11 (m, 1H), 0.82 (d, *J* = 6.6 Hz, 3H). ^{13}C NMR (126 MHz, $CDCl_3$) δ 144.8, 138.8, 133.3, 129.9, 128.0, 114.6, 69.1, 35.9, 35.7, 31.1, 28.8, 21.8, 19.1. HRMS (ESI) m/z: calcd for $C_{15}H_{21}O_3S$ $[M-H]^+$ 281.12059, found 281.12079.

Scheme S3: Synthesis of aggregation pheromone (4*R*,8*R*)-**1**.

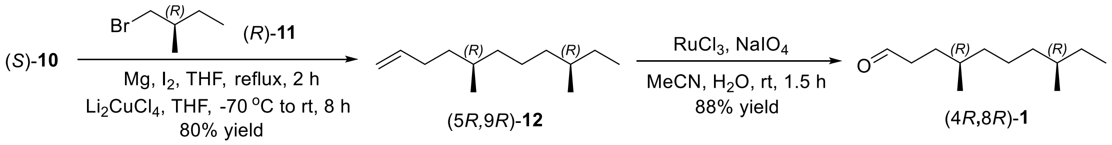

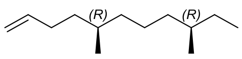

(5*R*,9*R*)-5,9-Dimethylundec-1-ene ((5*R*,9*R*)-**12**) (CAS 110595-31-8) [9]

To a 50 mL Schlenk flask was added Mg (0.24 g, 10.0 mmol) and I_2 (catalytic amount) in dry THF (6 mL) at room temperature, and (*R*)-1-bromo-2-methylbutane ((*R*)-**11**, 0.20 g, 1.34 mmol) was added. The resulting mixture was heated cautiously to initiate the reaction, and maintained simmering until the

solution became colorless. Subsequently, (*R*)-1-bromo-2-methylbutane ((*R*)-**11**, 0.80 g, 5.34 mmol) was added slowly. The reaction mixture was refluxed for 2 h and diluted with dry THF (6 mL) to afford (*R*)-(2-methylbutyl)magnesium bromide.

To another 50 mL Schlenk flask was added chiral tosylate (*S*)-**10** (0.47 g, 1.67 mmol) and dry THF (10 mL) at room temperature. After being cooled to −70 °C, Li_2CuCl_4 (5.0 mL, 0.1 M in THF, 0.50 mmol) was added and stirred for 20 min. Subsequently, the new prepared (*R*)-(2-methylbutyl)magnesium bromide was added dropwise over 1 h. After allowing the reaction mixture to warm to room temperature and stir for 8 h, the reaction was quenched with water (20 mL). The two layers were separated, and the aqueous layer was extracted with EtOAc (30 mL × 3). The ester extracts were combined with the organic layer, and was washed with brine (100 mL). The solution was dried over anhydrous Na_2SO_4 and concentrated by a rotary evaporator. The residue was purified by column chromatography on silica gel with an eluent of *n*-pentane to afford (5*R*,9*R*)-5,9-dimethylundec-1-ene ((5*R*,9*R*)-**12**, 0.24 g, 80% yield) as a colorless oil. $[\alpha]_D^{22}$ = –4.41 (c = 1.36, $CHCl_3$). ^1H NMR (500 MHz, $CDCl_3$) δ 5.81 (ddt, *J* = 17.0, 10.2, 6.6 Hz, 1H), 5.00 (dq, *J* = 17.1, 1.7 Hz, 1H), 4.92 (dq, *J* = 10.1, 1.4 Hz, 1H), 2.11 – 1.99 (m, 2H), 1.42 – 1.38 (m, 2H), 1.33 – 1.24 (m, 6H), 1.21 – 1.17 (m, 1H), 1.13 – 1.07 (m, 3H), 0.87 – 0.83 (m, 9H). ^{13}C NMR (126 MHz, $CDCl_3$) δ 139.7, 114.1, 37.5, 37.1, 36.4, 34.6, 32.5, 31.6, 29.6, 24.6, 19.7, 19.4, 11.6. HRMS (ESI) m/z: calcd for $C_{13}H_{27}$ $[M+H]^+$ 183.21254, found 183.21073.

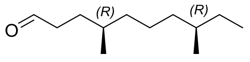

(4*R*,8*R*)-4,8-Dimethyldecanal ((4*R*,8*R*)-**1**) (CAS 85880-35-9) [10]

To a 10 mL Schlenk flask were added $NaIO_4$ (69.0 mg, 0.32 mmol) and a solution (CH_3CN/H_2O 6:1, 3.0 mL) at room temperature. Subsequently, chiral olefine (5*R*,9*R*)-**12** (30.0 mg, 0.16 mmol) and $RuCl_3$ (1.3 mg, 0.006 mmol) were

added and the mixture stirred for 1.5 h. The reaction was quenched with saturated $Na_2S_2O_3$ aqueous solution (5 mL). The two layers were separated, and the aqueous layer was extracted with Et_2OAc (10 mL × 3). The ester extracts were combined with the organic layer and washed with brine (30 mL). The solution was dried over anhydrous Na_2SO_4 and concentrated by rotary evaporation. The residue was a colorless liquid, which was (4*R*,8*R*)-4,8-dimethyldecanal ((4*R*,8*R*)-**1**, 26.0 mg, 88% yield). $[\alpha]_D^{22}$ = –5.00 (c = 1.36, $CHCl_3$). Lit.[11] $[\alpha]_D^{23}$ = –5.6 (c = 1.1, $CHCl_3$). ^1H NMR (500 MHz, $CDCl_3$) δ 9.78 (t, *J* = 2.0 Hz, 1H), 2.43 (tdd, *J* = 8.9, 6.0, 2.0 Hz, 2H), 1.69 – 1.64 (m, 1H), 1.46 – 1.41 (m, 2H), 1.35 – 1.25 (m, 6H), 1.14 – 1.06 (m, 3H), 0.89 – 0.84 (m, 9H). ^{13}C NMR (126 MHz, $CDCl_3$) δ 203.2, 41.9, 37.2, 37.0, 34.6, 32.6, 29.7, 29.1, 24.5, 19.5, 19.4, 11.6. HRMS (ESI) m/z: calcd for $C_{12}H_{26}O_2Na$ $[M+H_2O+Na]^+$ 225.18250, found 225.18239.

Scheme S4: Synthesis of aggregation pheromone (4*R*,8*S*)-**1**.

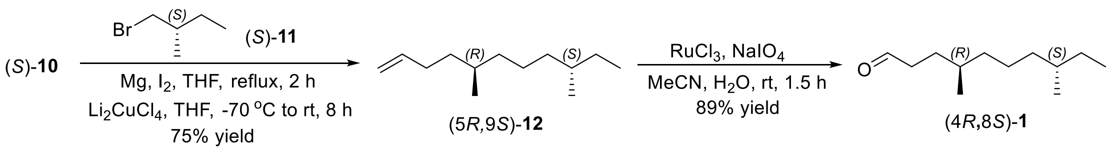

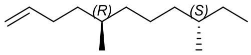

(5*R*,9*S*)-5,9-Dimethylundec-1-ene ((5*R*,9*S*)-**12**) (CAS 110595-35-2)

In a similar manner as described for chiral olefine (5*R*,9*R*)-**12**, the coupling of chiral tosylate (*S*)-**10** (0.47 g, 1.67 mmol) with (*S*)-(2-methylbutyl)magnesium bromide derived from Mg (0.24 g，10.0 mmol) and (*S*)-1-bromo-2-methylbutane ((*S*)-**11**, 1.00 g， 6.67 mmol) afforded ((5*R*,9*S*)-5,9-dimethylundec-1-ene ((5*R*,9*S*)-**12**, 0.23 g, 75% yield) as a colorless liquid. $[\alpha]_D^{22}$ = +4.44 (c = 1.62, $CHCl_3$).^1H NMR (500 MHz, $CDCl_3$) δ 5.84 – 5.79 (m, 1H), 5.00 (dq, *J* = 17.1, 1.7 Hz, 1H), 4.94 – 4.91 (m, 1H), 2.09 – 2.02 (m, 2H), 1.42 – 1.37 (m, 2H), 1.34 – 1.24 (m, 6H), 1.21 – 1.17 (m, 1H), 1.14 – 1.05 (m, 3H), 0.87 – 0.84 (m, 9H).

^{13}C NMR (126 MHz, $CDCl_3$) δ 139.7, 114.1, 37.5, 37.1, 36.4, 34.6, 32.5, 31.6, 29.6, 24.6, 19.7, 19.4, 11.6. HRMS (ESI) m/z: calcd for $C_{13}H_{27}$ $[M+H]^+$ 183.21073, found 183.21254.

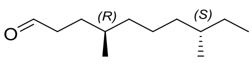

(4*R*,8*S*)-4,8-Dimethyldecanal ((4*R*,8*S*)-**1**) (CAS 85880-37-1)

In a similar manner as described for pheromone (4*R*,8*R*)-**1**, the oxidation of (5*R*,9*S*)-5,9-dimethylundec-1-ene ((5*R*,9*S*)-**12**, 182.0 mg, 1.0 mmol) with $NaIO_4$ (432.0 mg, 2.0 mmol) and $RuCl_3$ (7.0 mg, 0.035 mmol) afforded (4*R*,8*S*)-4,8-dimethyldecanal ((4*R*,8*S*)-**1**, 164.0 mg, 89% yield) as a colorless liquid. $[\alpha]_D^{22}$ = + 2.75 (c = 1.60, $CHCl_3$). Lit. [12] $[\alpha]_D^{23}$ = +5.1 (c = 6.00, $CHCl_3$). ^1H NMR (500 MHz, $CDCl_3$) δ 9.77 (t, *J* = 1.9 Hz, 1H), 2.45 – 2.40 (m, 2H), 1.69 – 1.64 (m, 1H), 1.46 – 1.41 (m, 2H), 1.35 – 1.25 (m, 6H), 1.14 – 1.06 (m, 3H), 0.89 – 0.84 (m, 9H). ^{13}C NMR (126 MHz, $CDCl_3$) δ 203.2, 41.9, 37.1, 36.9, 34.5, 32.5, 29.7, 29.1, 24.5, 19.5, 19.4, 11.5. HRMS (ESI) m/z: calcd for $C_{11}H_{22}O$ $[[M-CH_3+H]]^+$ 170.16652, found 170.16633.

Scheme S5: Synthesis of aggregation pheromone (4*S*,8*R*)-**1**.

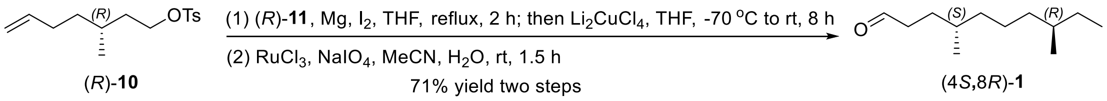

(*R*)-**10** (4*S*,8*R*)-**1**

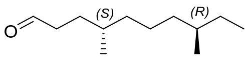

(4*S*,8*R*)-4,8-Dimethyldecanal ((4*S*,8*R*)-**1**) (CAS 85880-38-2)

In a similar manner as described for chiral olefine (5*R*,9*R*)-**12**, the coupling of chiral tosylate (*R*)-**10** (0.47 g, 1.67 mmol) with (*R*)-(2-methylbutyl)magnesium bromide derived from Mg (0.24 g, 10.0 mmol) and (*R*)-1-bromo-2-methylbutane ((*R*)-**11**, 1.00 g，6.67 mmol) afforded ((5*S*,9*R*)-5,9-dimethylundec-1-ene (0.23 g) as a colorless liquid. HRMS (ESI) m/z: calcd for $C_{13}H_{26}$ $[M]^+$ 183.2029, found 182.2024.

Then, similarly to the procedure for pheromone (4*R*,8*R*)-**1**, the oxidation of (5*S*,9*R*)-5,9-dimethylundec-1-ene (92.0 mg，0.5 mmol) with $NaIO_4$ (216.0 mg, 1.0 mmol) and $RuCl_3$ (4.0 mg, 0.0175 mmol) afforded (4*R*,8*S*)-4,8-dimethyldecanal ((4*S*,8*R*)-**1**, 87.0 mg, 71% yield two steps) as a colorless liquid. ^{1}H NMR (500 MHz, $CDCl_3$) δ 9.76 (t, *J* = 2.4 Hz, 1H), 2.42 – 2.37 (m, 1H), 2.25 – 2.20 (m, 1H), 1.45 – 1.40 (m, 1H), 1.35 – 1.32 (m, 2H), 1.31 – 1.28 (m, 6H), 1.15 – 1.08 (m, 3H), 0.89 – 0.84 (m, 9H). ^{13}C NMR (126 MHz, $CDCl_3$) δ 203.4, 41.9, 37.4, 36.8, 34.5, 32.5, 29.6, 29.0, 24.5, 19.5, 19.3, 11.5. HRMS (ESI) m/z: calcd for $C_{12}H_{24}ONa$ [M+Na]$^+$ 207.1719, found 207.1720.

Scheme S6: Synthesis of aggregation pheromone (4*S*,8*S*)-**1**.

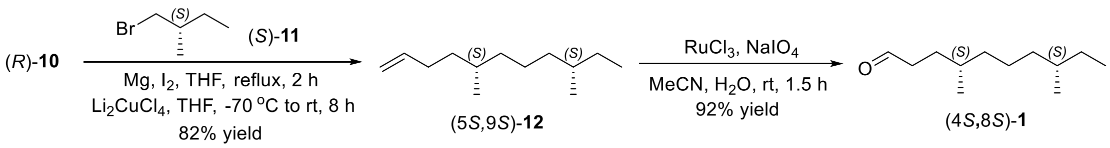

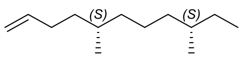

(5*S*,9*S*)-5,9-Dimethylundec-1-ene (5*S*,9*S*-**12**) (new compound)

In a similar manner as described for chiral olefine (5*R*,9*R*)-**12**, the coupling of chiral tosylate (*R*)-**10** (0.47 g, 1.67 mmol) with (*S*)-(2-methylbutyl)magnesium bromide derived from Mg (0.24 g，10.0 mmol) and (*S*)-1-bromo-2-methylbutane ((*S*)-**11**, 1.00 g，6.67 mmol) afforded (5*S*,9*R*)-5,9-dimethylundec-1-ene ((5*S*,9*S*)-**12**, 0.25 g, 82% yield) as a colorless liquid. $[\alpha]_D^{22}$ = +4.80 (c = 1.00, $CHCl_3$).^{1}H NMR (500 MHz, $CDCl_3$) δ 5.82 (ddt, *J* = 16.9, 10.2, 6.7 Hz, 1H), 5.00 (dq, *J* = 17.1, 1.7 Hz, 1H), 4.92 (dq, *J* = 10.1, 1.4 Hz, 1H), 2.11 – 1.99 (m, 2H), 1.42 – 1.38 (m, 2H), 1.32 – 1.26 (m, 6H), 1.21 – 1.18 (m, 1H), 1.12 – 1.04 (m, 3H), 0.87 – 0.84 (m, 9H). ^{13}C NMR (126 MHz, $CDCl_3$) δ 139.7, 114.1, 37.5, 37.1, 36.4, 34.6, 32.5, 31.6, 29.6, 24.6, 19.7, 19.4, 11.6. HRMS (ESI) m/z: calcd for $C_{13}H_{26}$ [M]$^+$ 182.20290, found 182.20235.

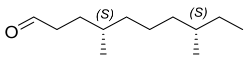

(4*S*,8*S*)-4,8-Dimethyldecanal ((4*S*,8*S*)-**1**) (CAS 85880-36-0)

In a similar manner as described for pheromone (4*R*,8*R*)-**1**), the oxidation of (5*S*,9*S*)-5,9-dimethylundec-1-ene ((5*S*,9*S*)-**12**, 92.0 mg, 0.5 mmol) with $NaIO_4$ (216.0 mg,1.0 mmol), and $RuCl_3$ (4.0 mg, 0.0175 mmol) afforded (4*S*,8*S*)-4,8-dimethyldecanal ((4*S*,8*S*)-**1**, 85.0 mg, 92% yield) as a colorless liquid. $[\alpha]_D^{22}$ = +18.00 (c = 1.00, $CHCl_3$). Lit.[13] $[\alpha]_D^{25}$ = +7.20 (c = 4.17, $CHCl_3$). ^{1}H NMR (500 MHz, $CDCl_3$) δ 9.78 (d, *J* = 3.0 Hz, 1H), 2.45 – 2.40 (m, 2H), 1.69 – 1.64 (m, 1H), 1.46 – 1.40 (m, 2H), 1.35 – 1.22 (m, 6H), 1.11 – 1.05 (m, 3H), 0.89 – 0.84 (m, 9H). ^{13}C NMR (126 MHz, $CDCl_3$) δ 203.2, 41.9, 37.2, 37.0, 34.5, 32.6, 29.7, 29.1, 24.5, 19.5, 19.4, 11.6. HRMS (ESI) m/z: calcd for $C_{12}H_{23}OKNa$ [M+K+Na-H]$^+$ 245.12782, found 245.12840.

3. Research on the optical purity of chiral alcohols (*R*)- and (*S*)-4

Scheme S7: Synthesis of Mosher esters **14**,**15**, and**16**.

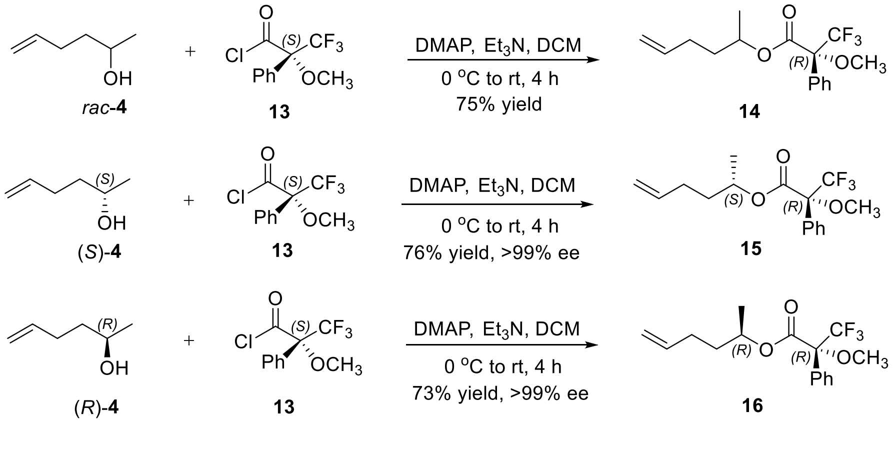

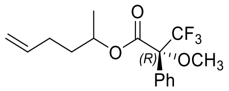

Hex-5-en-2-yl (*R*)-3,3,3-trifluoro-2-methoxy-2-phenylpropanoate **14** (new compound) [14,15]

To a 10 mL Schlenk flask were added hex-5-en-2-ol (*rac*-**4**, 20.0 mg, 0.2 mmol) and DCM (2 mL) at room temperature. After being cooled to 0 °C, DMAP (24.0 mg, 0.2 mmol) and Et_3N (101.0 mg, 1.0 mmol) were added and stirred for 5 min. Subsequently, (*S*)-MTPACl (**13**, 51.0 mg, 0.2 mmol) was added. After allowing the reaction solution to warm to room temperature and maintain for 4 h, the reaction was quenched with water (2 mL). The two layers were separated, and the aqueous layer was extracted with DCM (2 mL × 3). The dichloromethane extracts were combined with the organic layer and washed with brine (6 mL). The solution was dried over anhydrous Na_2SO_4 and concentrated by rotary evaporation at low temperature. The residue was purified by column chromatography on silica gel with an eluent of petroleum ether/ethyl acetate 10:1 to afford hex-5-en-2-yl (*R*)-3,3,3-trifluoro-2-methoxy-2-phenylpropanoate (**14**, 47.0 mg, 75% yield) as a colorless oil. ^{1}H NMR (500 MHz, $CDCl_3$) δ 7.55 – 7.52 (m, 2H), 7.42 – 7.39 (m, 3H), 5.79 – 5.77 (m, 1H), 5.17 – 5.15 (m, 1H), 5.04 – 4.93 (m, 2H), 3.57 – 3.55 (m, 3H), 2.10 – 2.08 (m, 1H), 1.97 – 1.95 (m, 1H), 1.82 – 1.74 (m, 1H), 1.64 – 1.60 (m, 1H), 1.35 (d, *J* = 6.3 Hz, 1.5H), 1.27 (d, *J* = 6.3 Hz, 1.5H). ^{13}C NMR (126 MHz, $CDCl_3$) δ 166.3, 166.2, 137.40, 137.37, 132.7, 132.5, 129.7, 128.52, 128.50, 127.5, 127.4, 123.5 (q, *J* = 293.5 Hz), 115.6, 115.5, 84.6 (q, *J* = 27.2 Hz), 73.7, 73.5, 55.5, 34.93, 34.90, 29.6, 29.4, 19.9, 19.6. ^{19}F NMR (471 MHz, $CDCl_3$) δ -71.41, -71.43. HRMS (ESI) m/z: calcd for $C_{16}H_{19}O_3F_3$ $[M]^+$ 316.1286, found 316.1280.

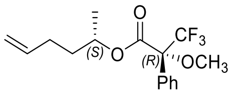 (*S*)-Hex-5-en-2-yl (*R*)-3,3,3-trifluoro-2-methoxy-2-phenylpropanoate **15** (new compound)

In a similar manner as described for Mosher ester **14**, the esterification of (*S*)-hex-5-en-2-ol ((*S*)-**4**, 20.0 mg, 0.2 mmol) with (*S*)-MTPACl (**13**, 51.0 mg, 0.2 mmol) afforded (*S*)-hex-5-en-2-yl (*R*)-3,3,3-trifluoro-2-methoxy-2-phenylpropanoate (**15**, 48.0 mg, 76% yield) as a colorless oil. ^{1}H NMR (500 MHz, $CDCl_3$) δ 7.53 (dd, *J* = 7.1, 3.0 Hz, 2H), 7.42 – 7.39 (m, 3H), 5.81 – 5.75 (m, 1H), 5.17 – 5.14 (m, 1H), 5.04 – 4.98 (m, 2H), 3.55 (d, *J* = 1.3 Hz, 3H), 2.12 – 2.08 (m, 2H), 1.82 – 1.79 (m, 1H), 1.68 – 1.64 (m, 1H), 1.27 (d, *J* = 6.3 Hz, 3H). ^{13}C NMR (126 MHz, $CDCl_3$) δ 166.3, 137.4, 132.5, 129.7, 128.5, 127.5, 127.0, 123.5 (q, *J* = 288.8 Hz), 115.6, 84.8 (q, *J* = 27.7 Hz), 73.7, 55.5, 34.9, 29.6, 19.6. ^{19}F NMR (471 MHz, $CDCl_3$) δ -71.44. HRMS (ESI) m/z: calcd for $C_{16}H_{19}O_3F_3$ $[M]^+$ 316.1286, found 316.1292.

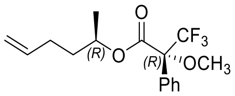 (*R*)-Hex-5-en-2-yl (*R*)-3,3,3-trifluoro-2-methoxy-2-phenylpropanoat **16** (new compound)

In a similar manner as described for Mosher ester **14**, the esterification of (*R*)-hex-5-en-2-ol ((*R*)-**4**, 20.0 mg, 0.2 mmol) with (*S*)-MTPACl (**13**, 51.0 mg, 0.2 mmol) afforded (*R*)-hex-5-en-2-yl (*R*)-3,3,3-trifluoro-2-methoxy-2-phenylpropanoate (**16**, 46.0 mg, 73% yield) as a colorless oil. ^{1}H NMR (500 MHz, $CDCl_3$) δ 7.55 (dd, *J* = 6.9, 3.0 Hz, 2H), 7.41 – 7.39 (m, 3H), 5.75 – 5.68 (m, 1H), 519 – 5.13 (m, 1H), 4.97 – 4.94 (m, 2H), 3.57 (d, *J* = 1.5 Hz, 3H), 2.04 – 1.93 (m, 2H), 1.78 – 1.71 (m, 1H), 1.64 – 1.58 (m, 1H), 1.35 (d, *J* = 6.3 Hz, 3H). ^{13}C NMR (126 MHz, $CDCl_3$) δ 166.2, 137.4, 132.7, 129.7, 128.5, 127.4, 127.0, 123.6 (q, *J* = 289.3 Hz), 115.5, 84.5 (q, *J* = 27.7 Hz), 73.5, 55.5, 34.9, 29.4, 19.9. ^{19}F NMR (471 MHz, $CDCl_3$) δ -71.41. HRMS (ESI) m/z: calcd for $C_{16}H_{18}O_3F_3$ $[M-H]^+$ 315.12026 found 315.12035.

4. ^{1}H and ^{13}C NMR spectra of the products

Figure S1: ^{1}H NMR Spectrum of (*R*)-hex-5-en-2-ol ((*R*)-**4**) (500 MHz, CDCl3).

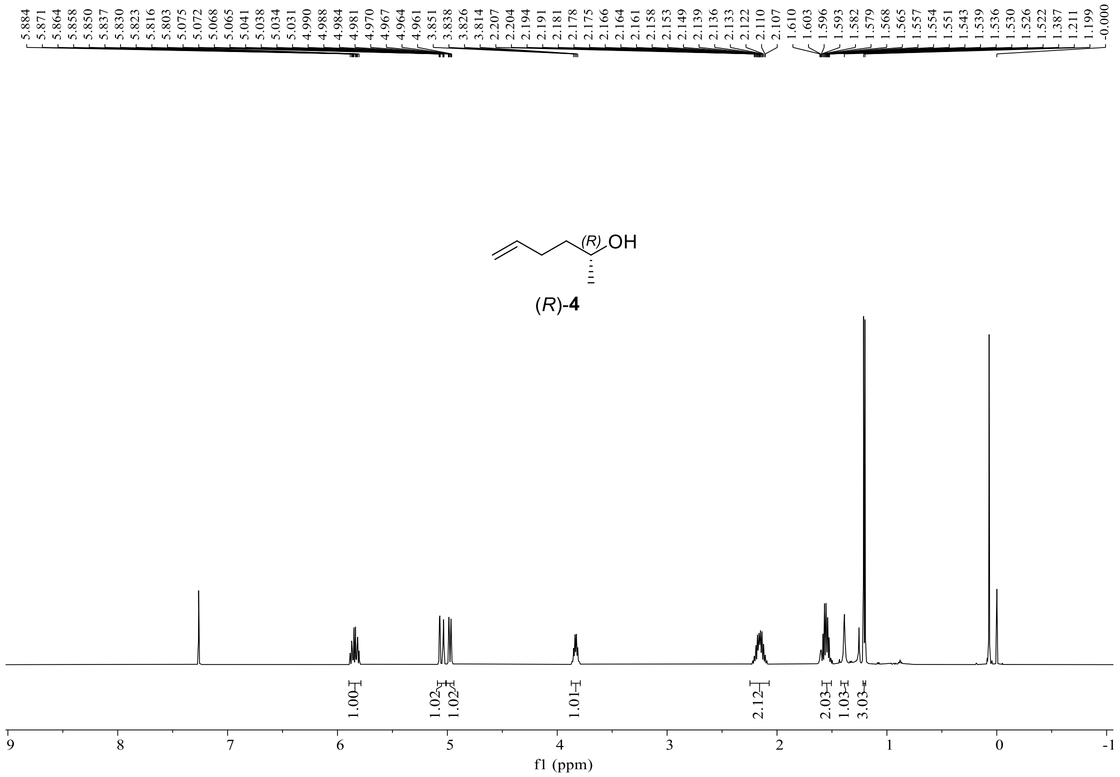

Figure S2: ^{13}C NMR Spectrum of (*R*)-hex-5-en-2-ol ((*R*)-**4**) (126 MHz, CDCl3).

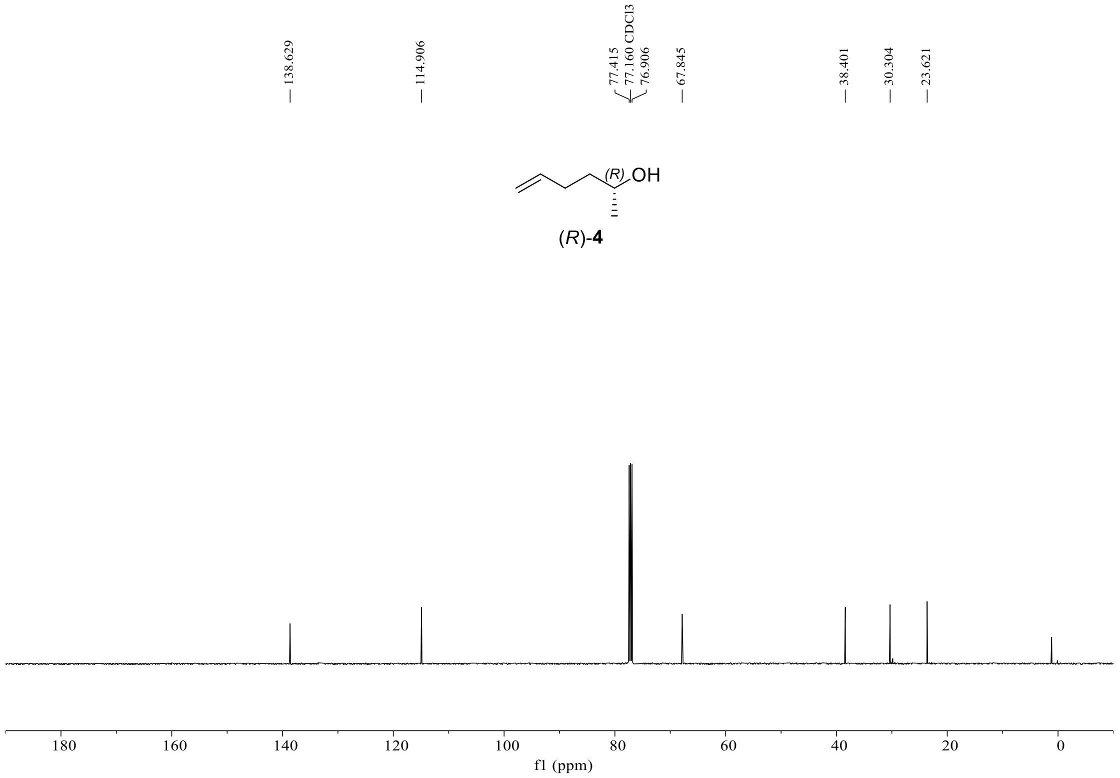

Figure S3: ^{1}H NMR Spectrum of (*R*)-hex-5-en-2-yl 4-methylbenzenesulfonate ((*R*)-**5**) (500 MHz, $CDCl_3$).

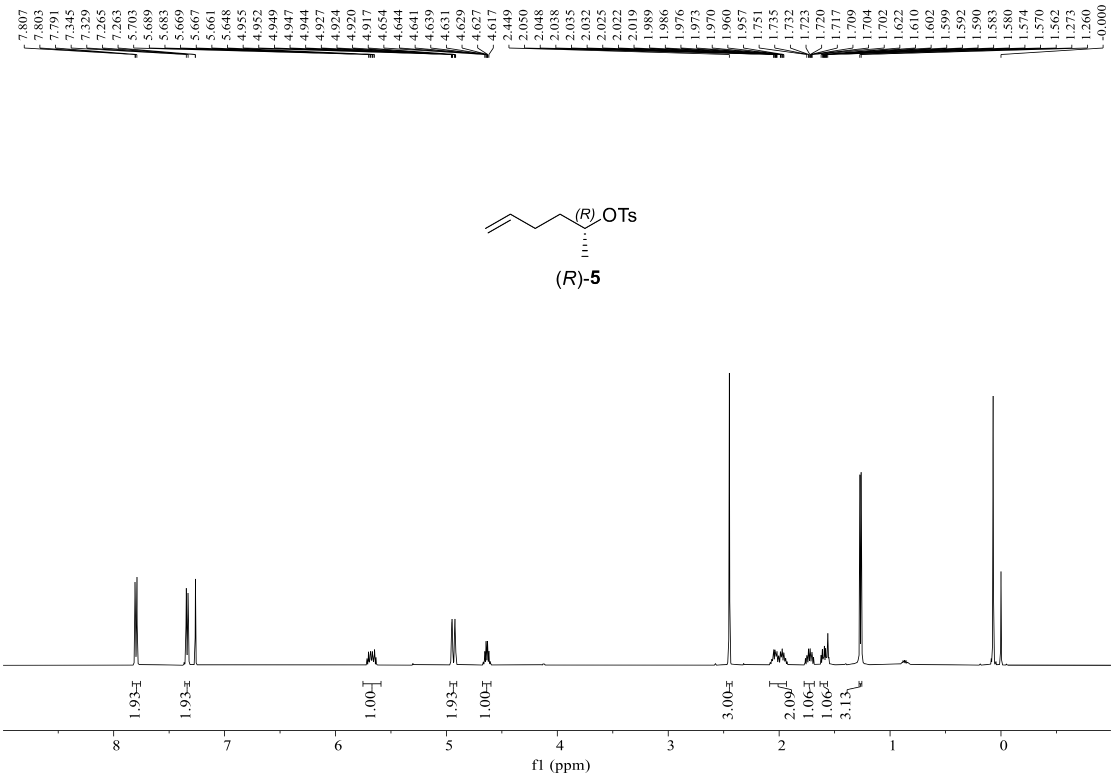

Figure S4: ^{13}C NMR Spectrum of (*R*)-hex-5-en-2-yl 4-methylbenzenesulfonate ((*R*)-**5**) (126 MHz, $CDCl_3$).

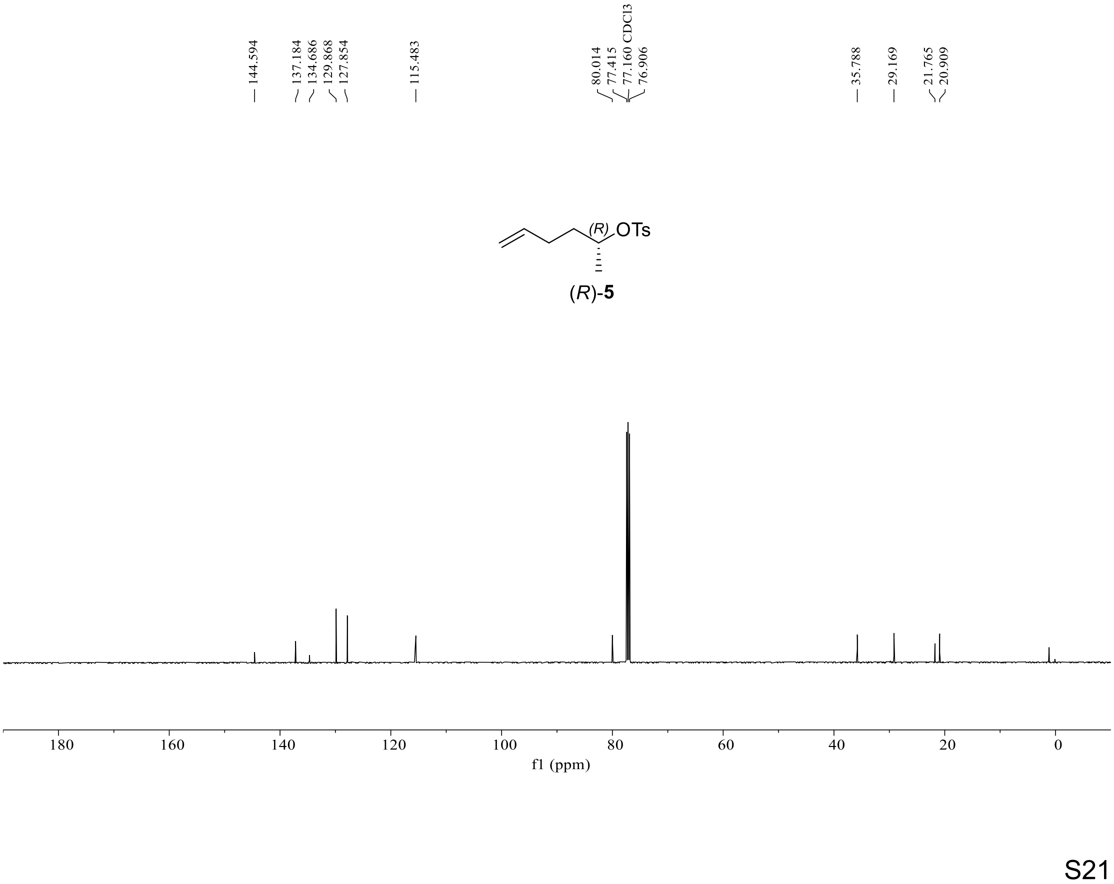

Figure S5: ^{1}H NMR Spectrum of diethyl (*S*)-2-(hex-5-en-2-yl) malonate ((*S*)-**6**) (500 MHz, CDCl$_3$).

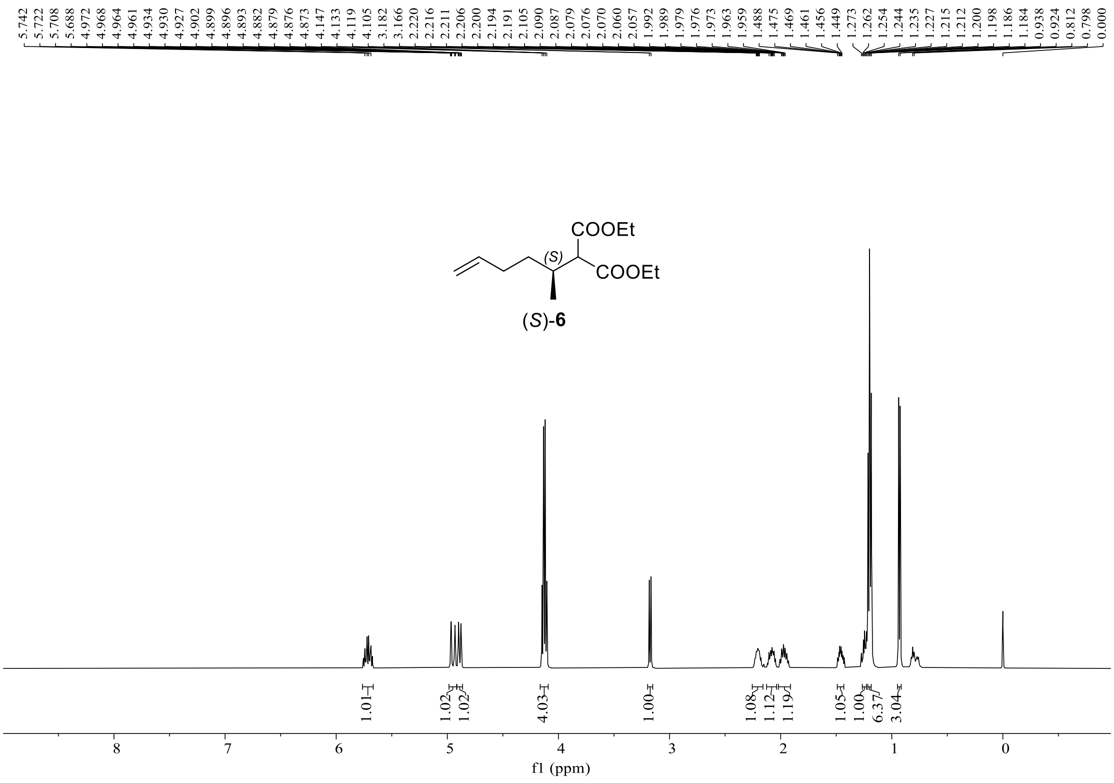

Figure S6: ^{13}C NMR Spectrum of diethyl (*S*)-2-(hex-5-en-2-yl) malonate ((*S*)-**6**) (126 MHz, CDCl$_3$).

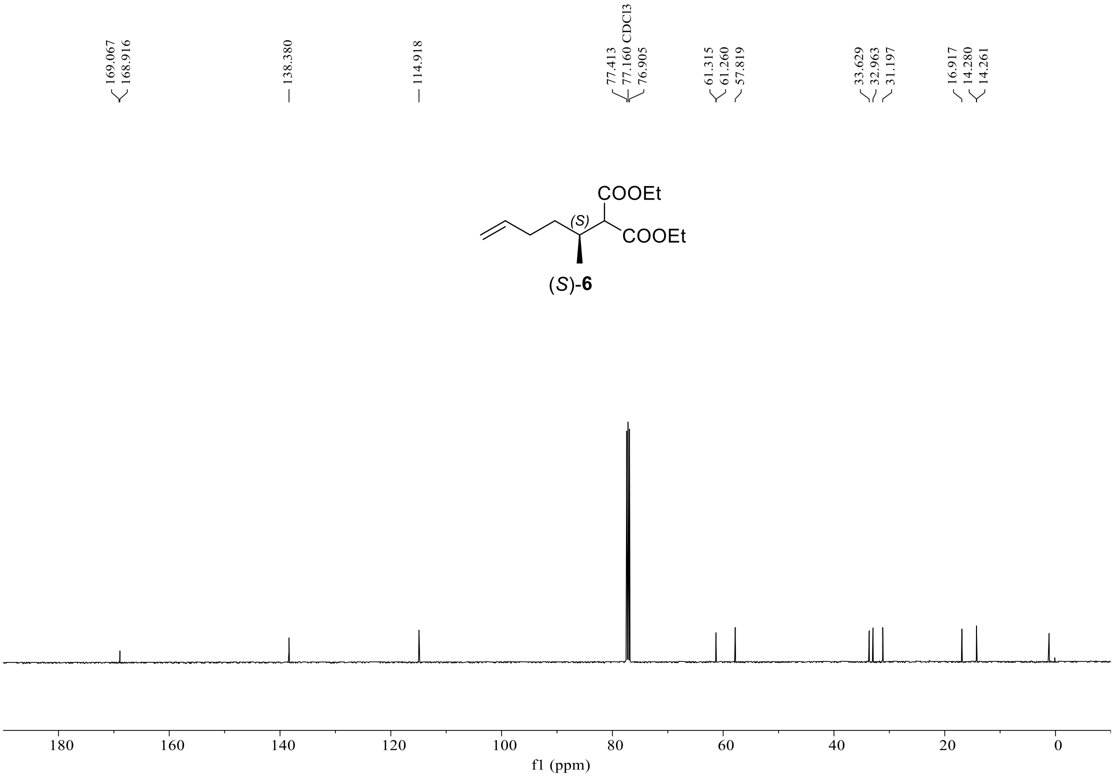

Figure S7: ^{1}H NMR Spectrum of (*S*)-2-(hex-5-en-2-yl) malonic acid ((*S*)-**7**) (500 MHz, CD_3SOCD_3).

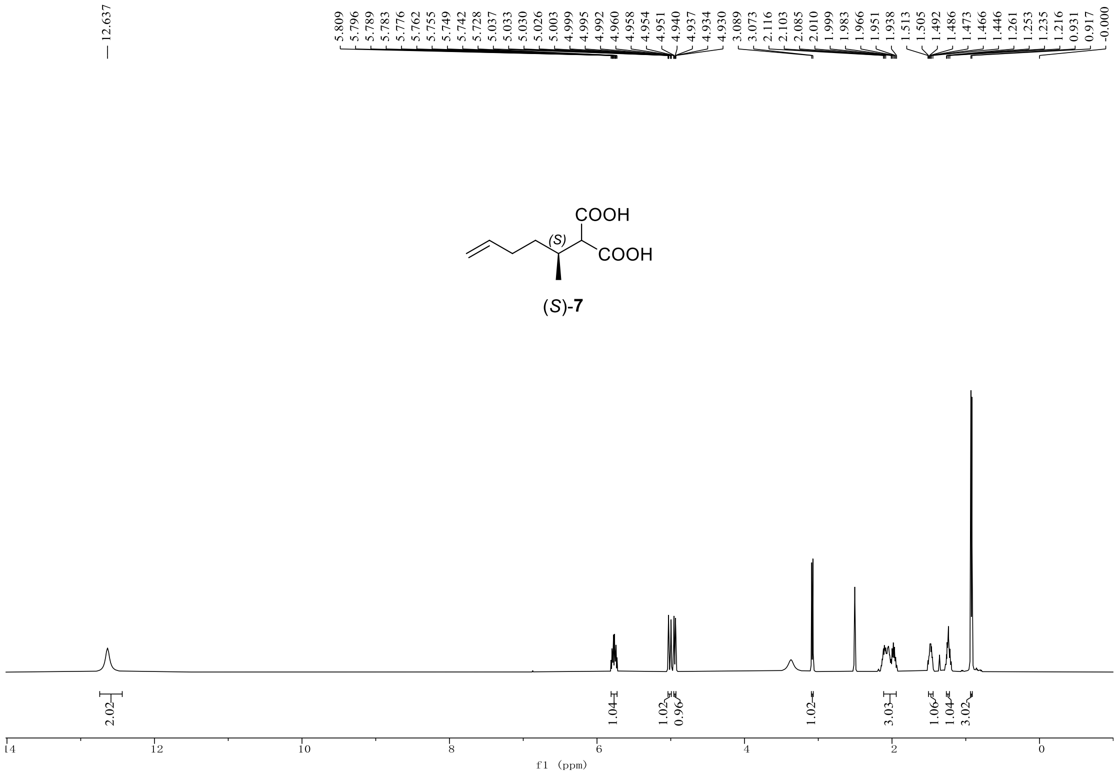

Figure S8: ^{13}C NMR Spectrum of (*S*)-2-(hex-5-en-2-yl) malonic acid ((*S*)-**7**) (126 MHz, CD_3SOCD_3).

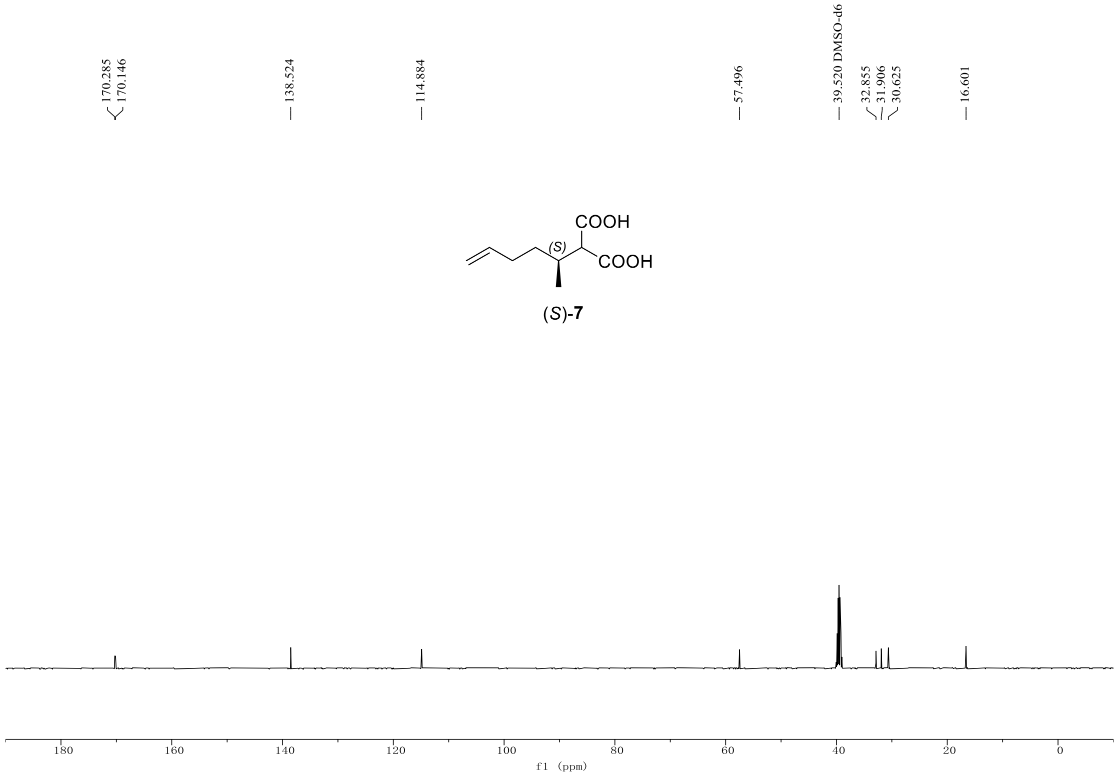

Figure S9: ^{1}H NMR Spectrum of (*S*)-3-methylhept-6-enoic acid ((*S*)-**8**) (500 MHz, CD_3SOCD_3).

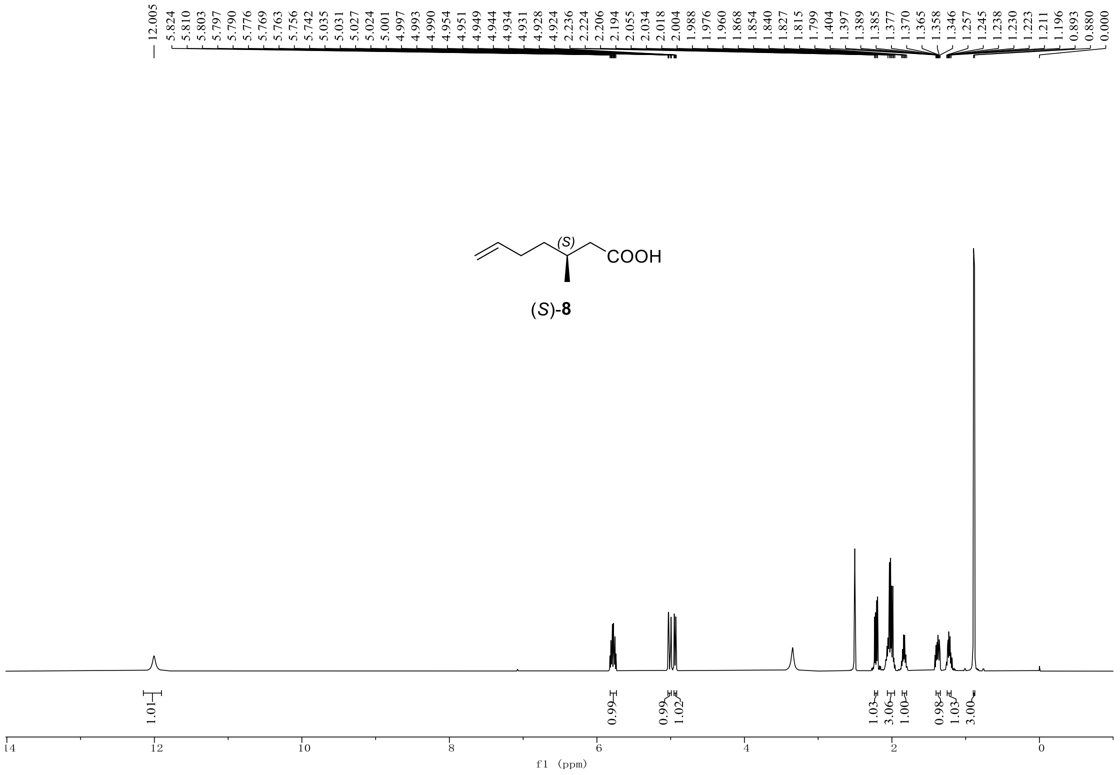

Figure S10: ^{13}C NMR Spectrum of (*S*)-3-methylhept-6-enoic acid ((*S*)-**8**) (126 MHz, CD_3SOCD_3).

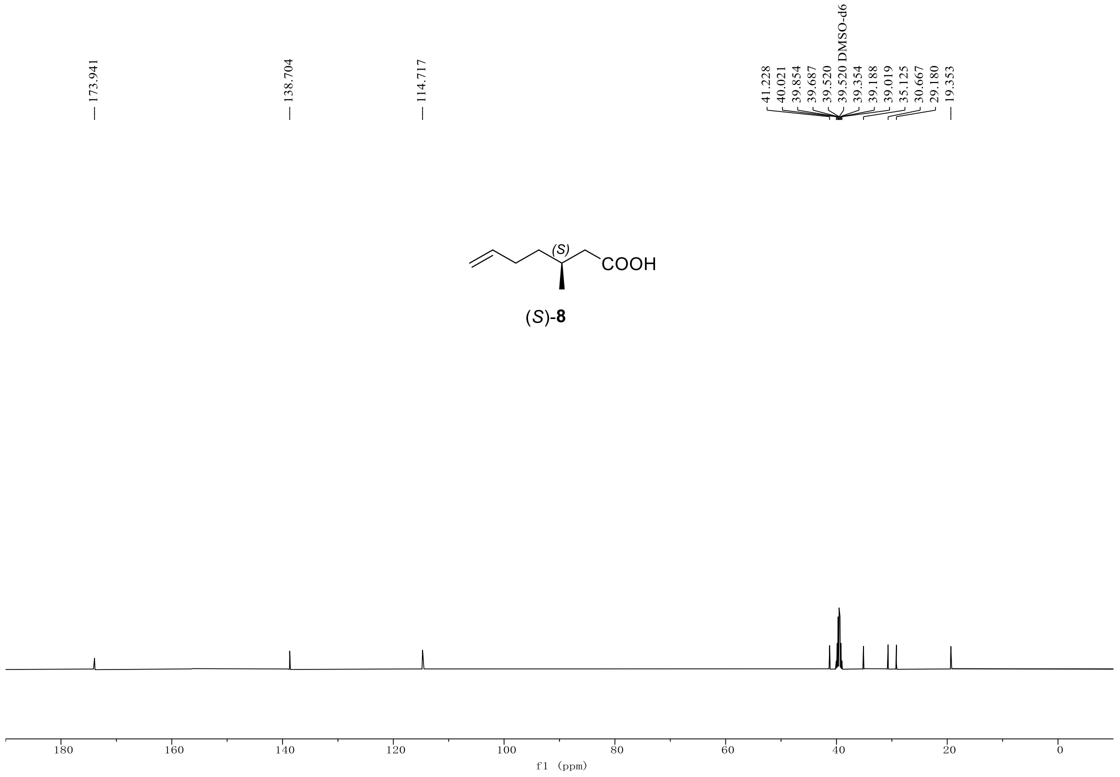

Figure S11: ^{1}H NMR Spectrum of (*S*)-3-methylhept-6-en-1-ol ((*S*)-**9**) (500 MHz, CDCl$_3$).

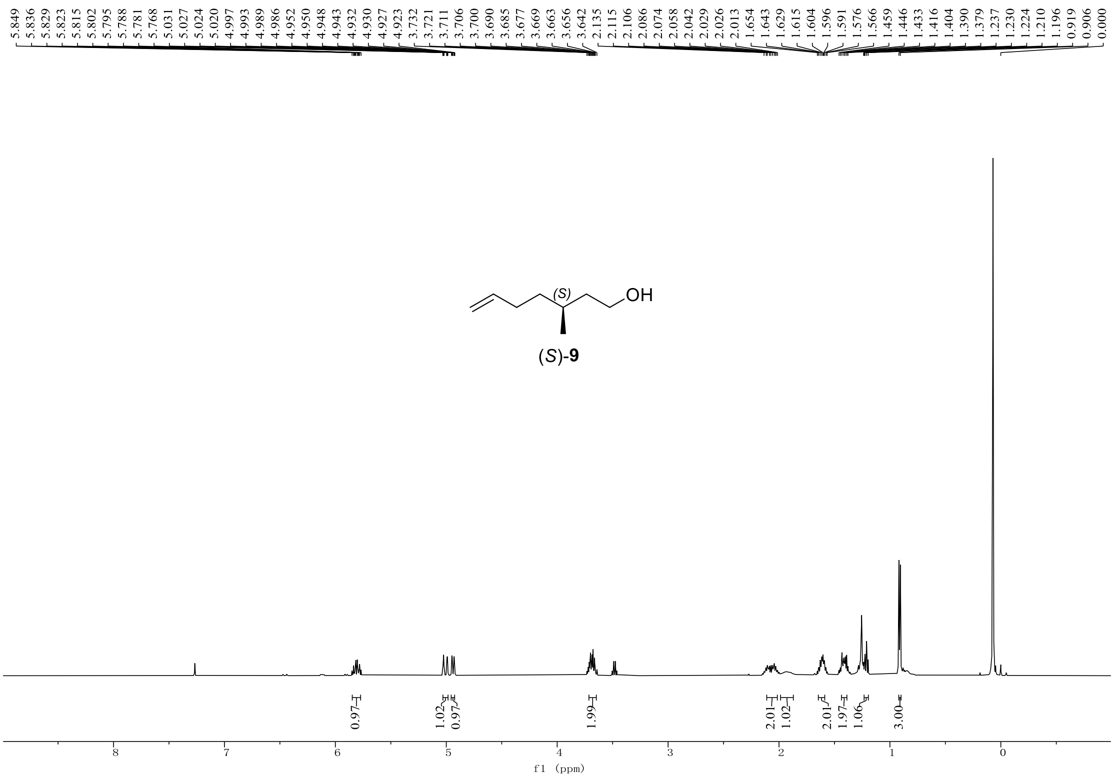

Figure S12: ^{13}C NMR Spectrum of (*S*)-3-methylhept-6-en-1-ol ((*S*)-**9**) (126 MHz, CDCl$_3$).

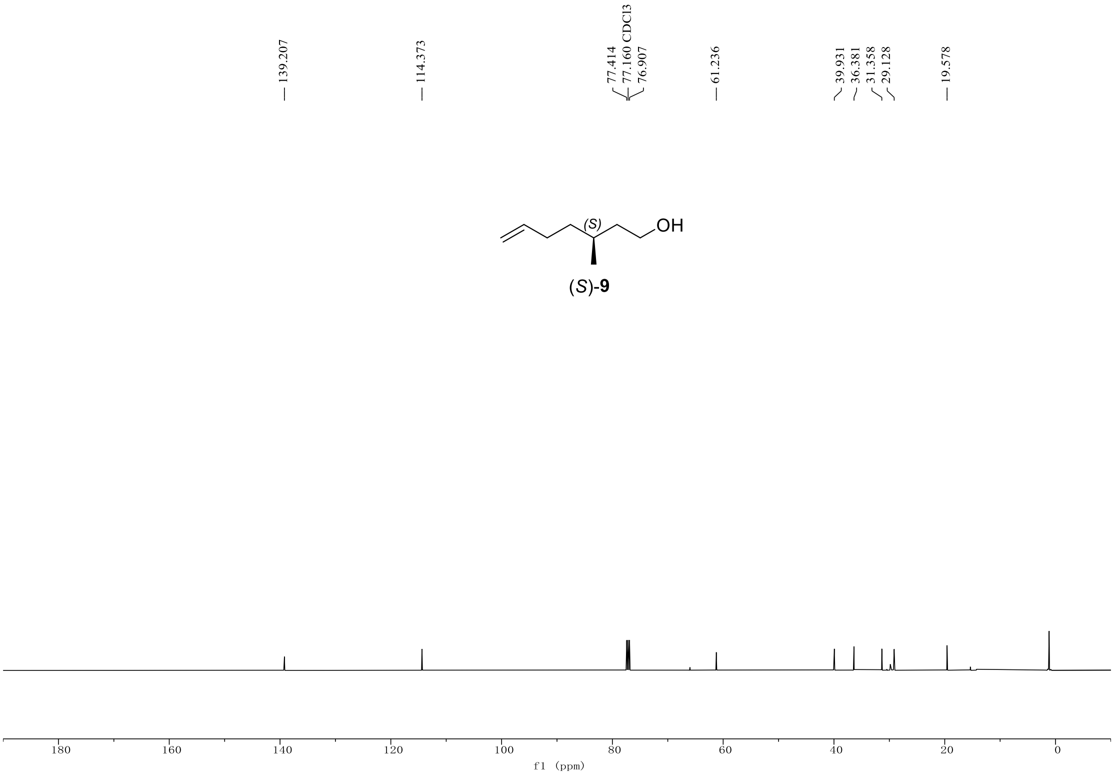

Figure S13: ^{1}H NMR Spectrum of (*S*)-3-methylhept-6-en-1-yl 4-methylbenzenesulfonate ((*S*)-**10**) (500 MHz, $CDCl_3$).

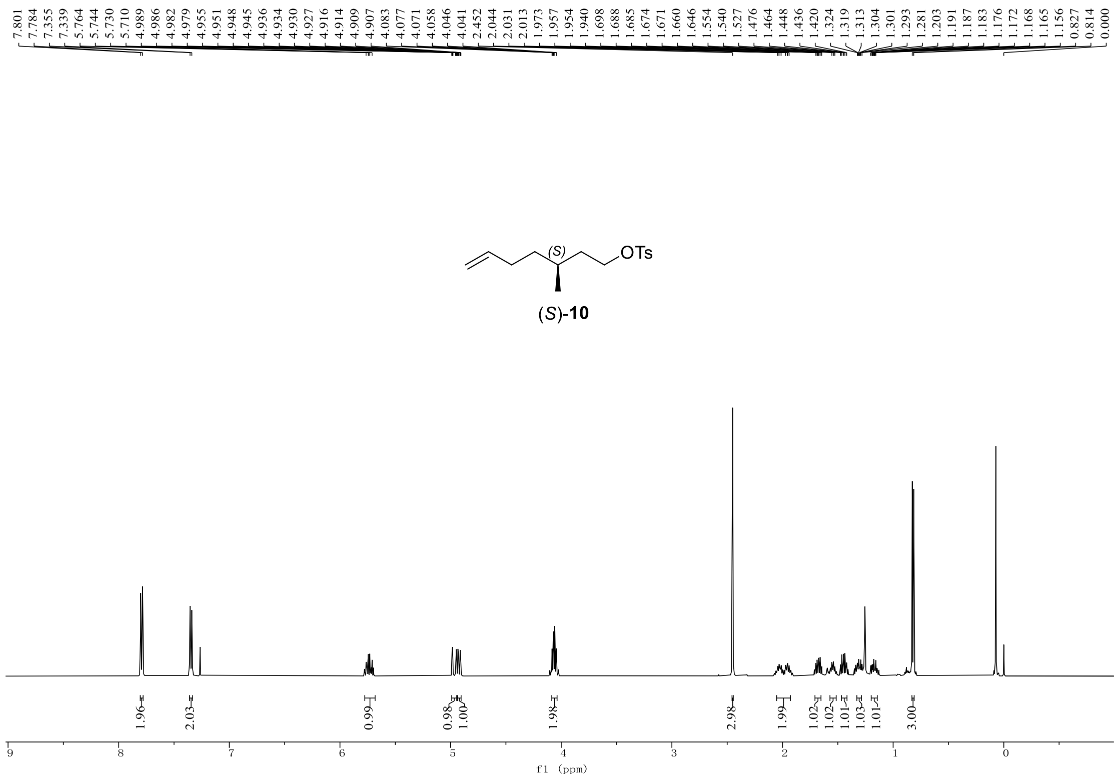

Figure S14: ^{13}C NMR Spectrum of (*S*)-3-methylhept-6-en-1-yl 4-methylbenzenesulfonate ((*S*)-**10**) (126 MHz, $CDCl_3$).

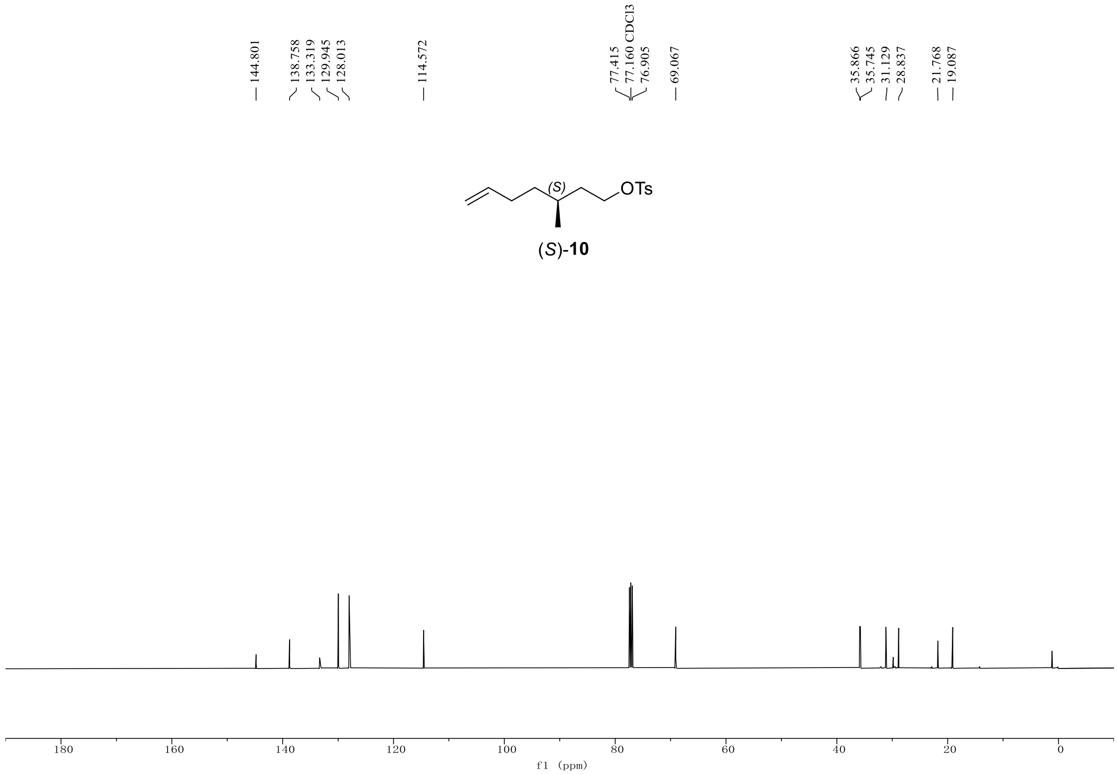

Figure S15: ^{1}H NMR Spectrum of (*S*)-hex-5-en-2-ol ((*S*)-**4**) (500 MHz, CDCl3).

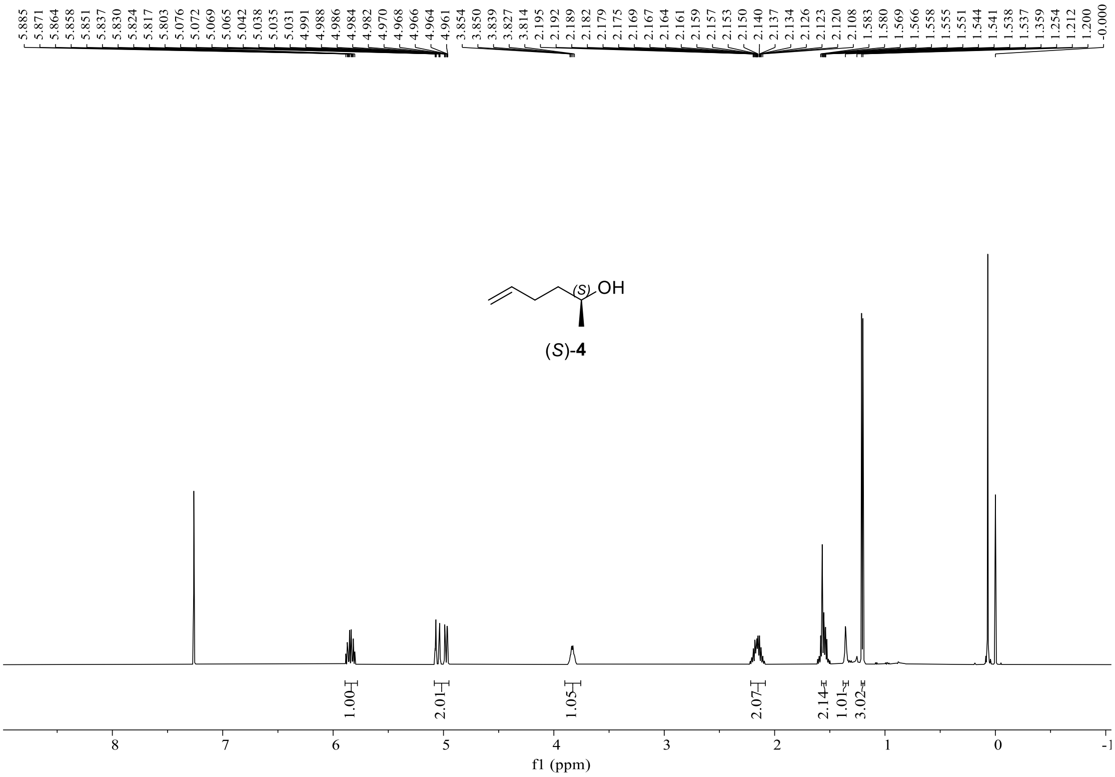

Figure S16: ^{13}C NMR Spectrum of (*S*)-hex-5-en-2-ol ((*S*)-**4**) (126 MHz, CDCl3).

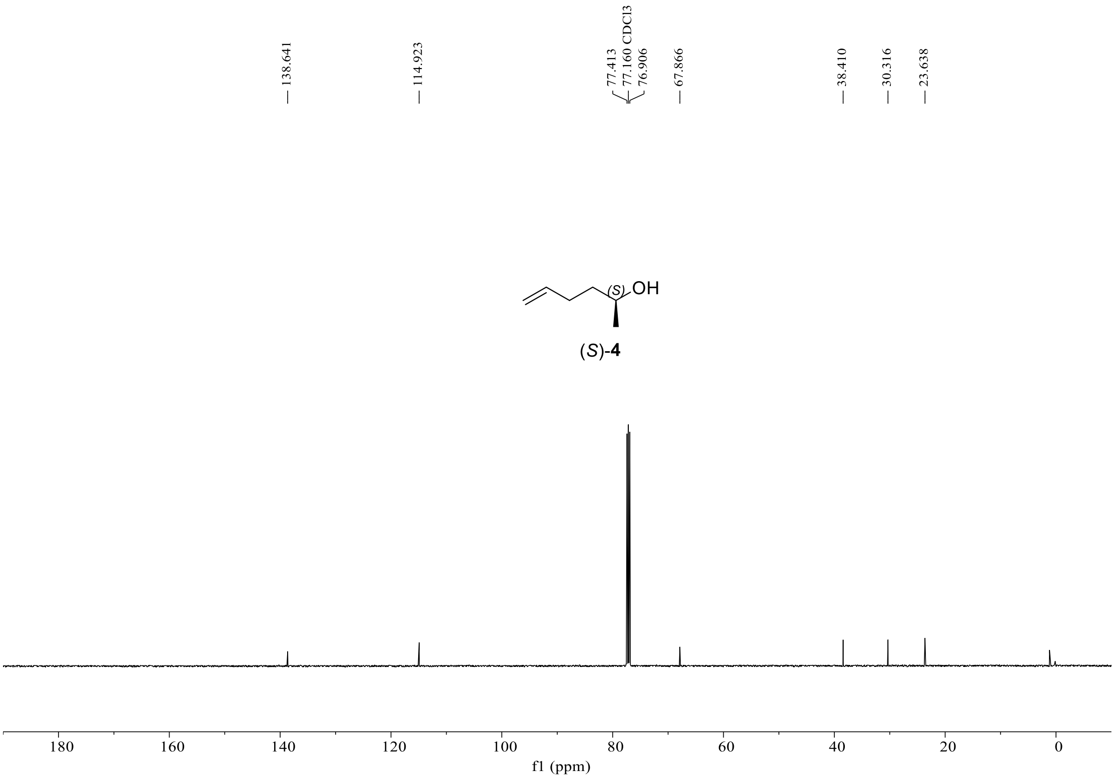

Figure S17: ^{1}H NMR Spectrum of (*S*)-hex-5-en-2-yl 4-methylbenzenesulfonate ((*S*)-**5**) (500 MHz, $CDCl_3$).

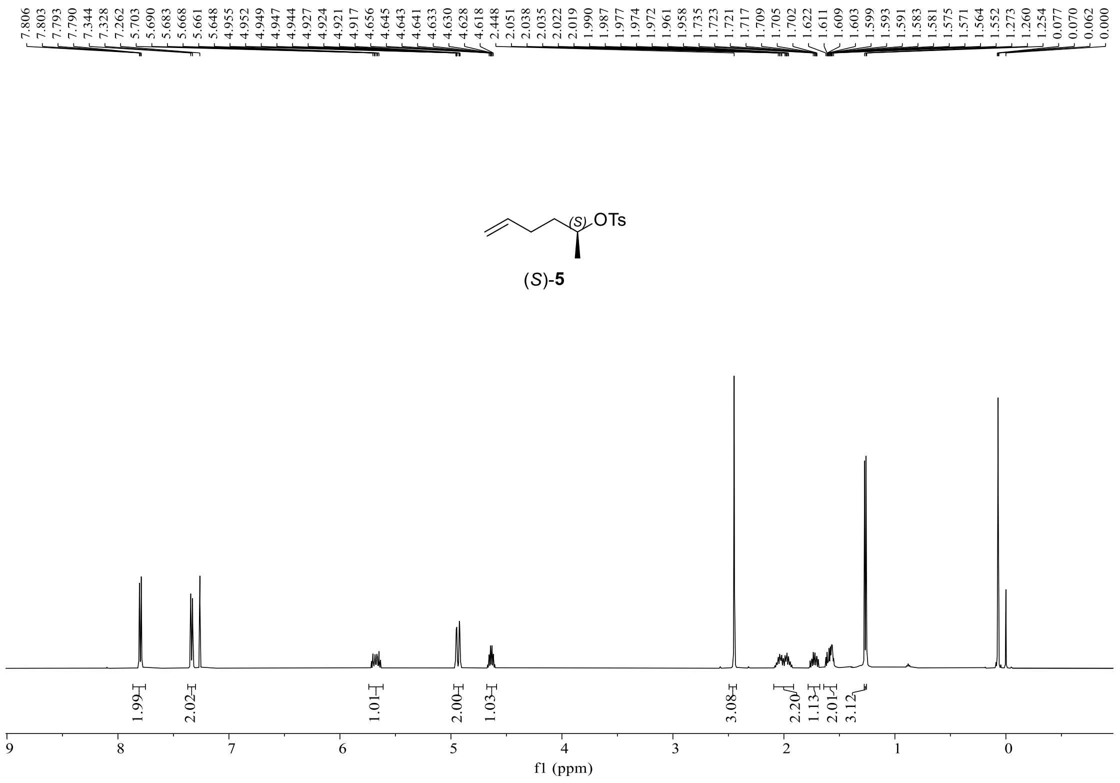

Figure S18: ^{13}C NMR Spectrum of (*S*)-hex-5-en-2-yl 4-methylbenzenesulfonate ((*S*)-**5**) (126 MHz, $CDCl_3$).

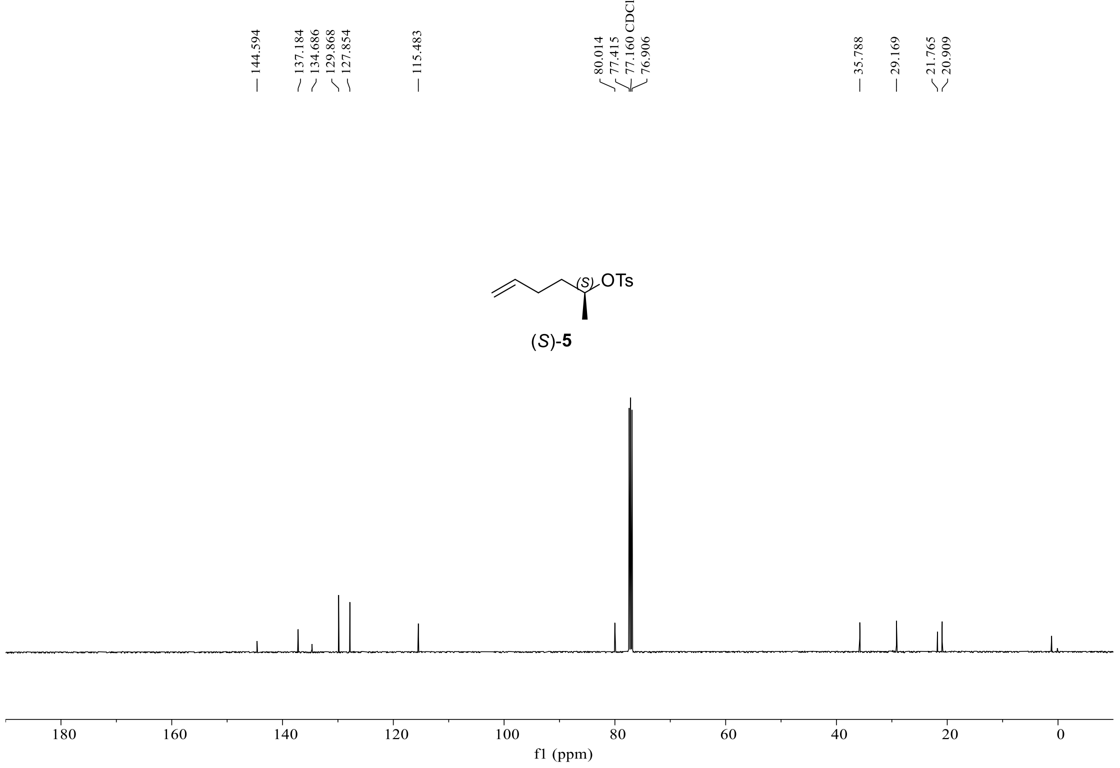

Figure S19: ^{1}H NMR Spectrum of diethyl (*R*)-2-(hex-5-en-2-yl) malonate ((*R*)-**6**) (500 MHz, CDCl$_3$).

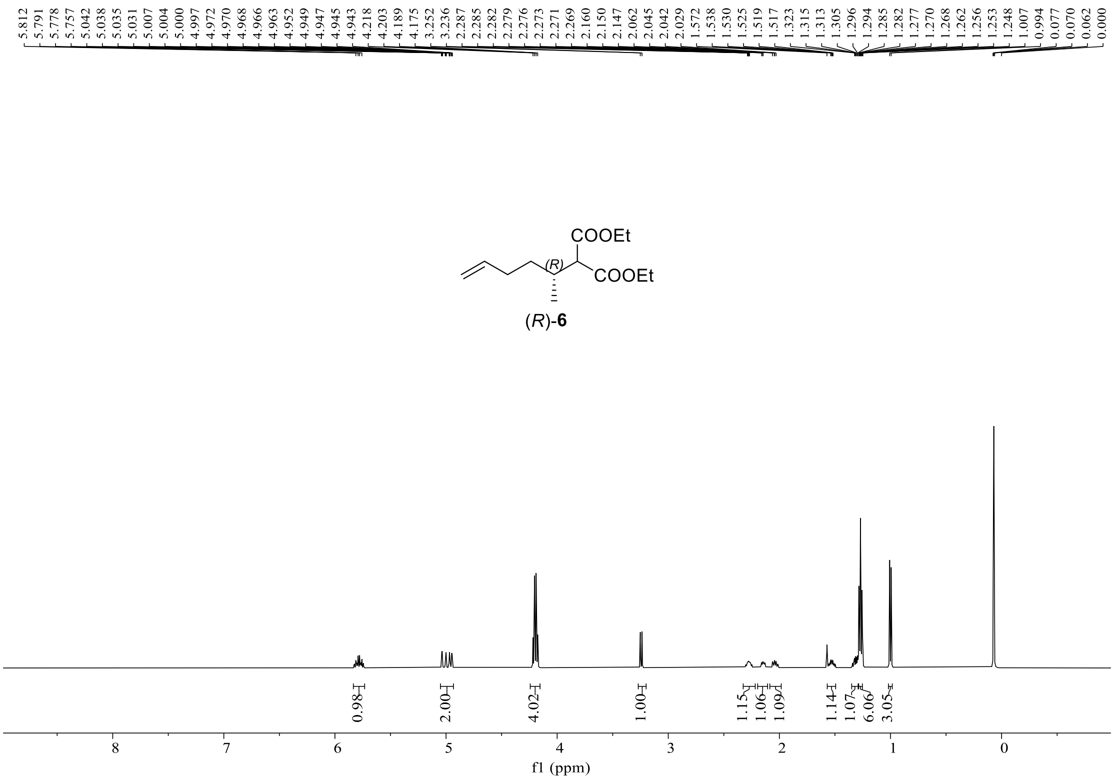

Figure S20: ^{13}C NMR Spectrum of (*R*)-2-(hex-5-en-2-yl) malonate ((*R*)-**6**) (126 MHz, CDCl$_3$).

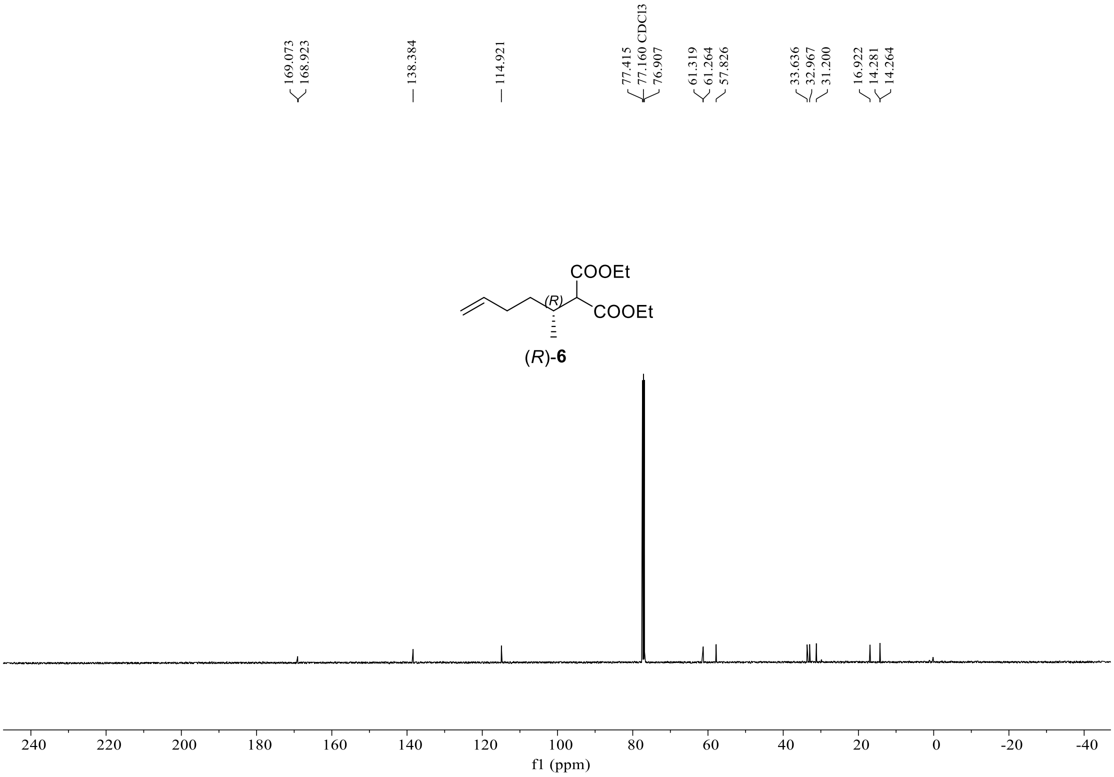

Figure S21: ^{1}H NMR Spectrum of diethyl (*R*)-2-(hex-5-en-2-yl) malonic acid ((*R*)-**7**) (500 MHz, $CDCl_3$).

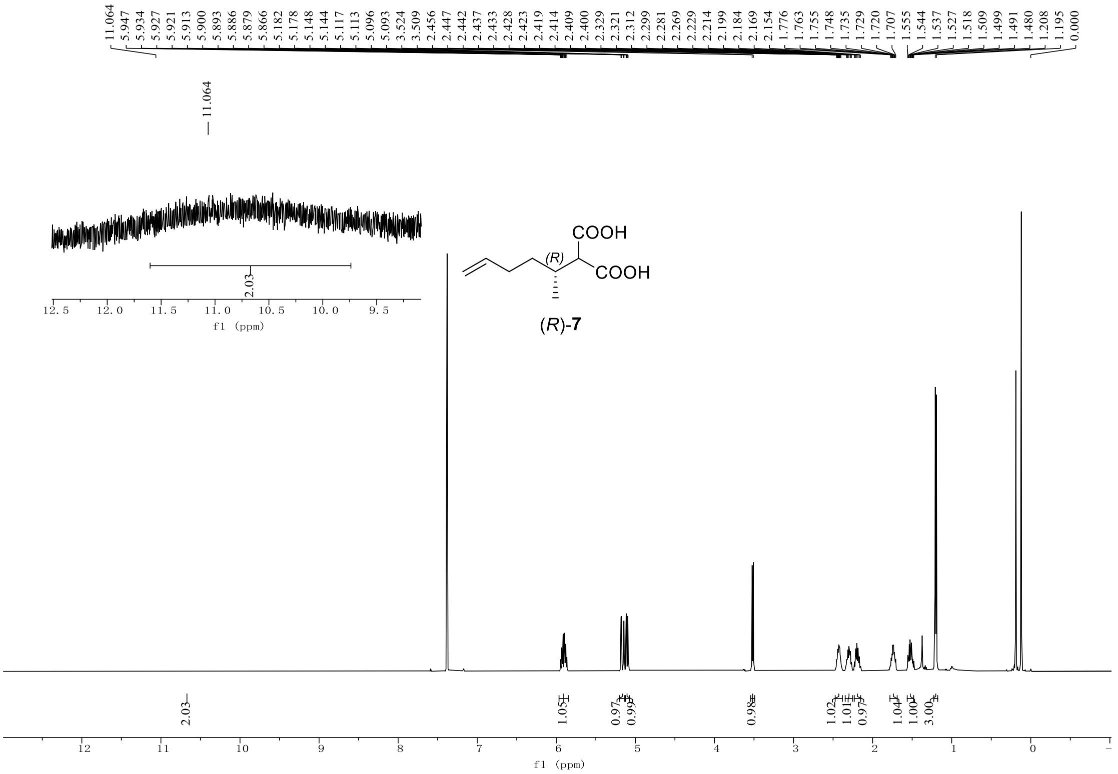

Figure S22: ^{13}C NMR Spectrum of (*R*)-2-(hex-5-en-2-yl) malonic acid ((*R*)-**7**) (126 MHz, $CDCl_3$).

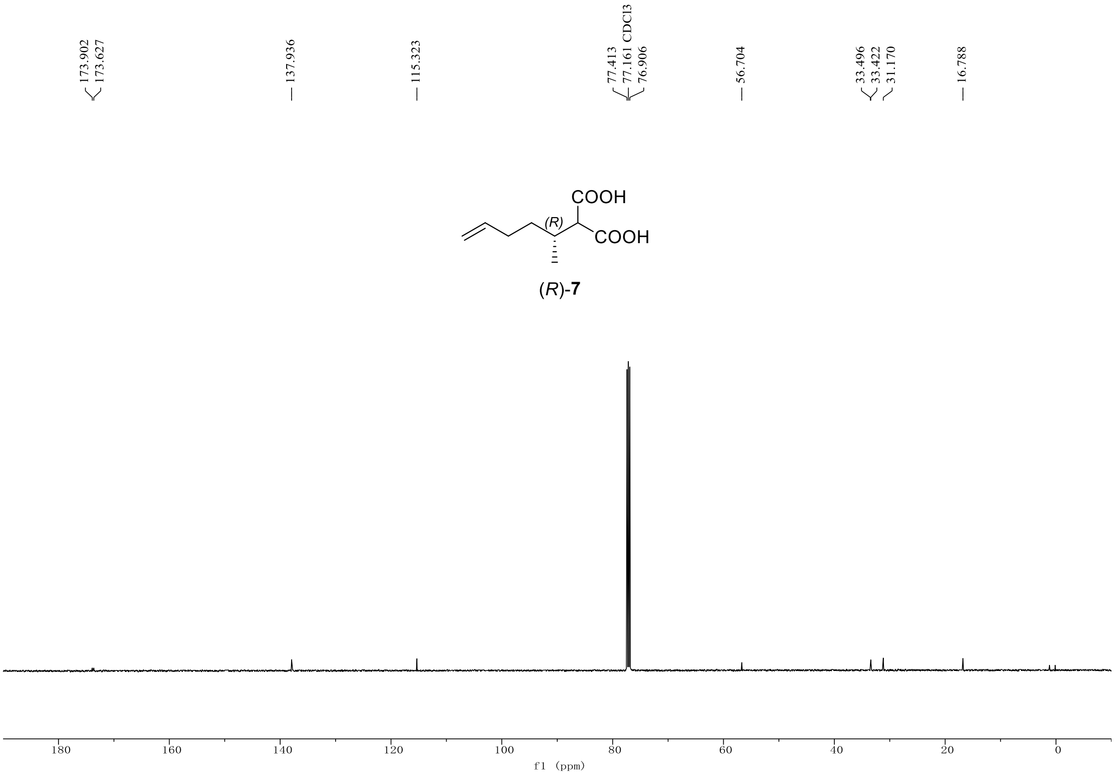

Figure S23: ^{1}H NMR Spectrum of (*R*)-3-methylhept-6-enoic acid ((*R*)-**8**) (500 MHz, $CDCl_3$).

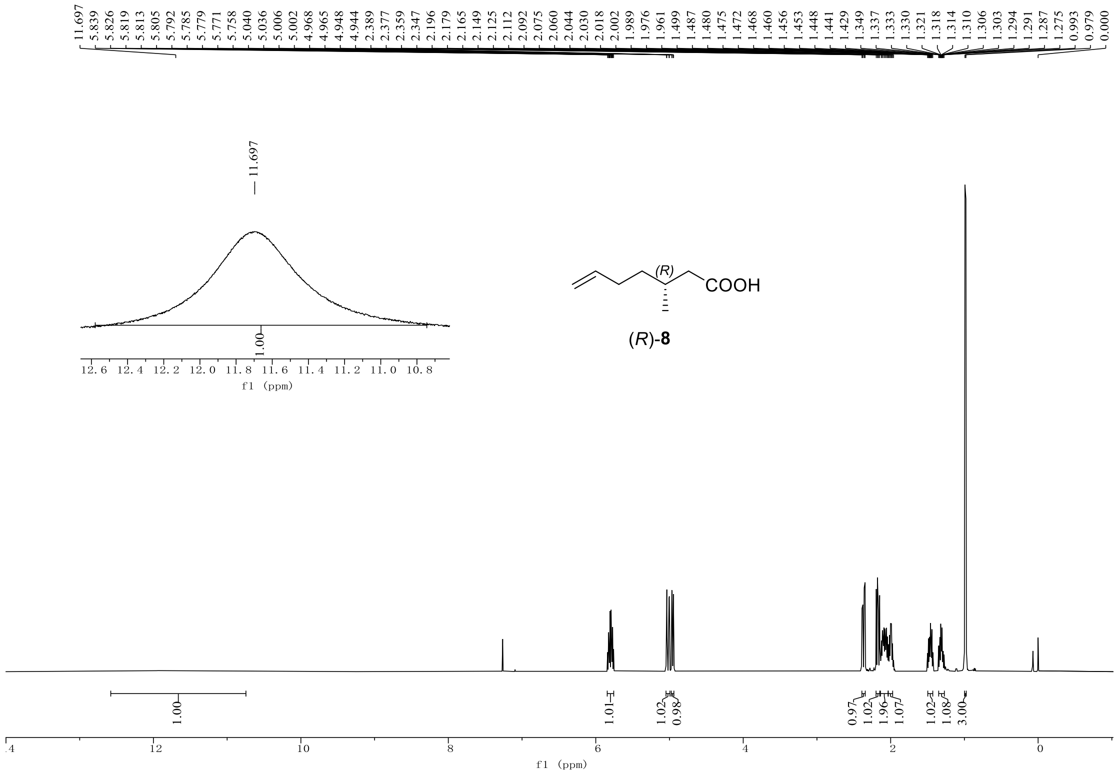

Figure S24: ^{13}C NMR Spectrum of (*R*)-3-methylhept-6-enoic acid ((*R*)-**8**) (126 MHz, $CDCl_3$).

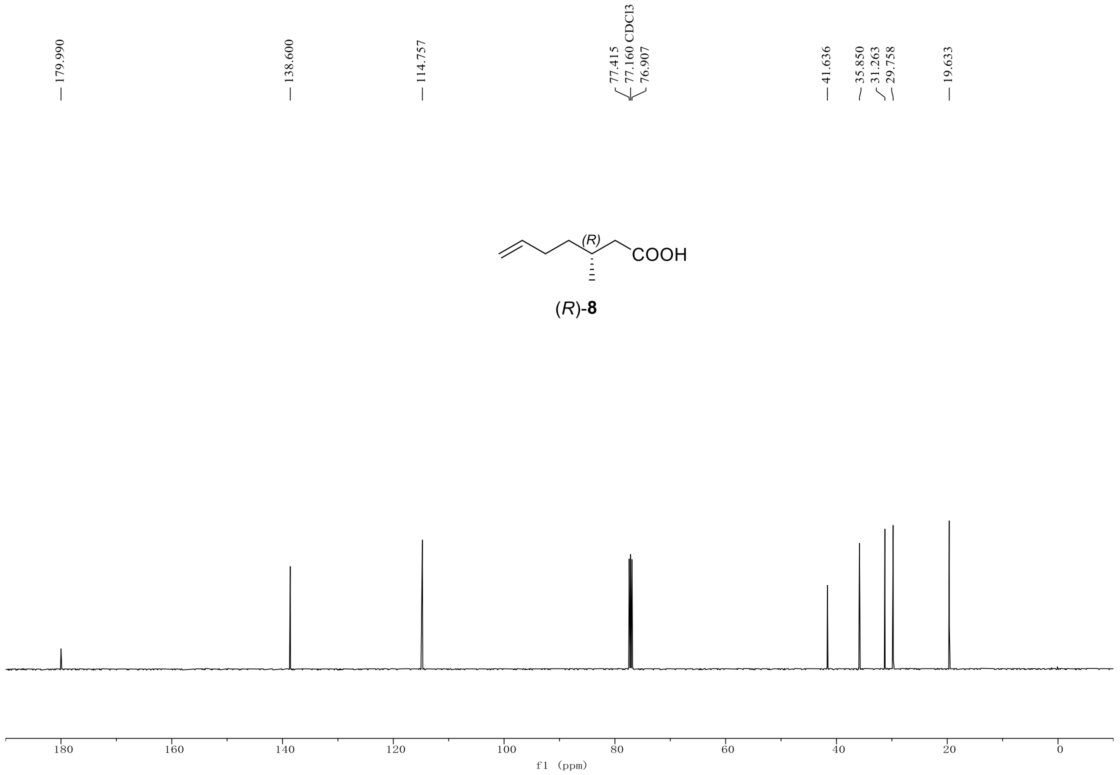

Figure S25: ^{1}H NMR Spectrum of (*R*)-3-methylhept-6-en-1-ol ((*R*)-**9**) (500 MHz, $CDCl_3$).

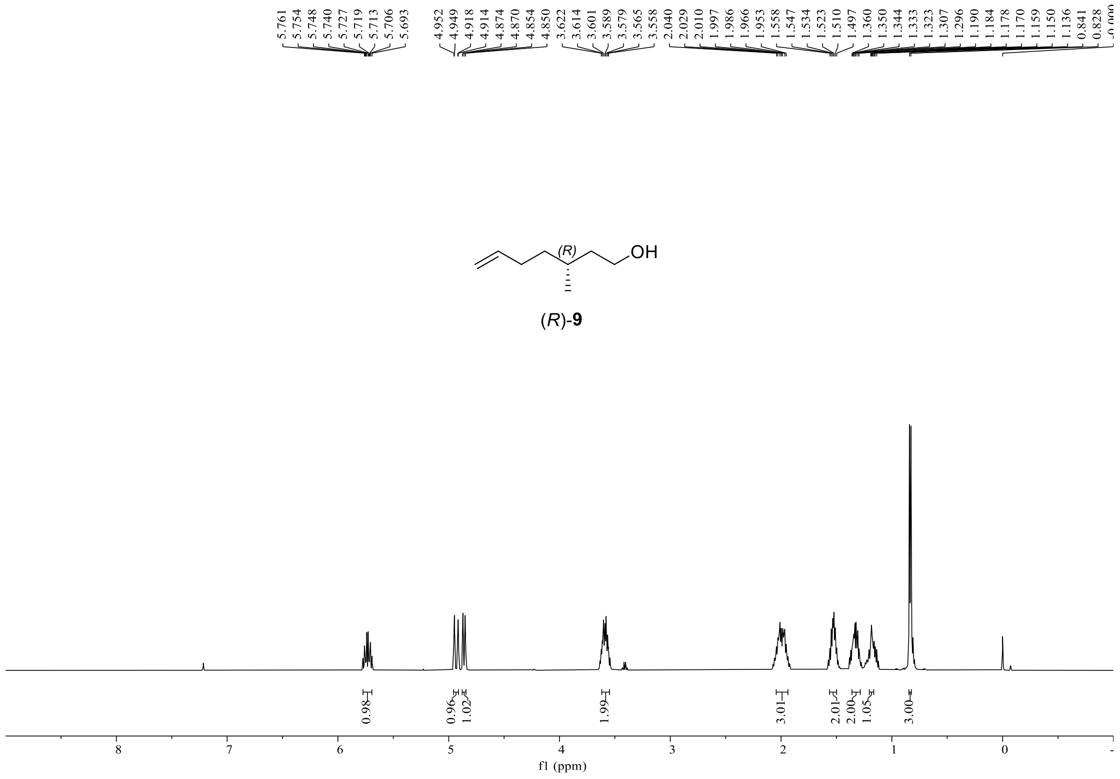

Figure S26: ^{13}C NMR Spectrum of (*R*)-3-methylhept-6-en-1-ol ((*R*)-**9**) (126 MHz, $CDCl_3$).

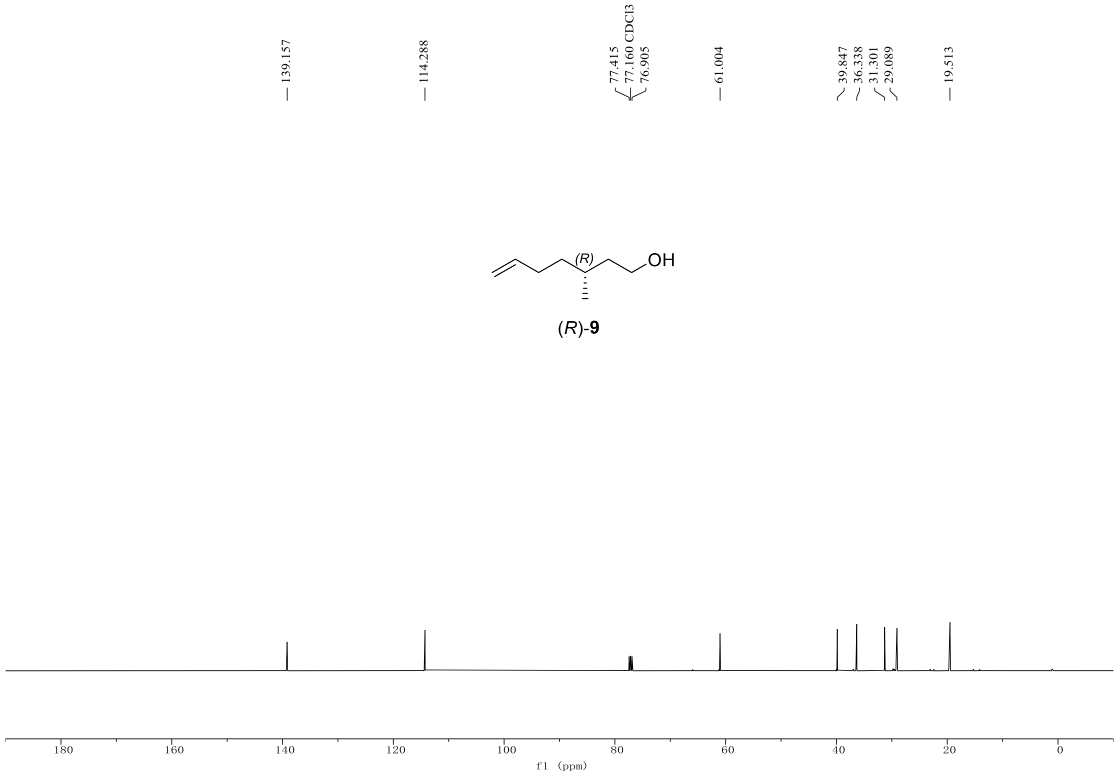

Figure S27: ^{1}H NMR Spectrum of (*R*)-3-methylhept-6-en-1-yl 4-methylbenzenesulfonate ((*R*)-**10**) (500 MHz, CDCl$_3$).

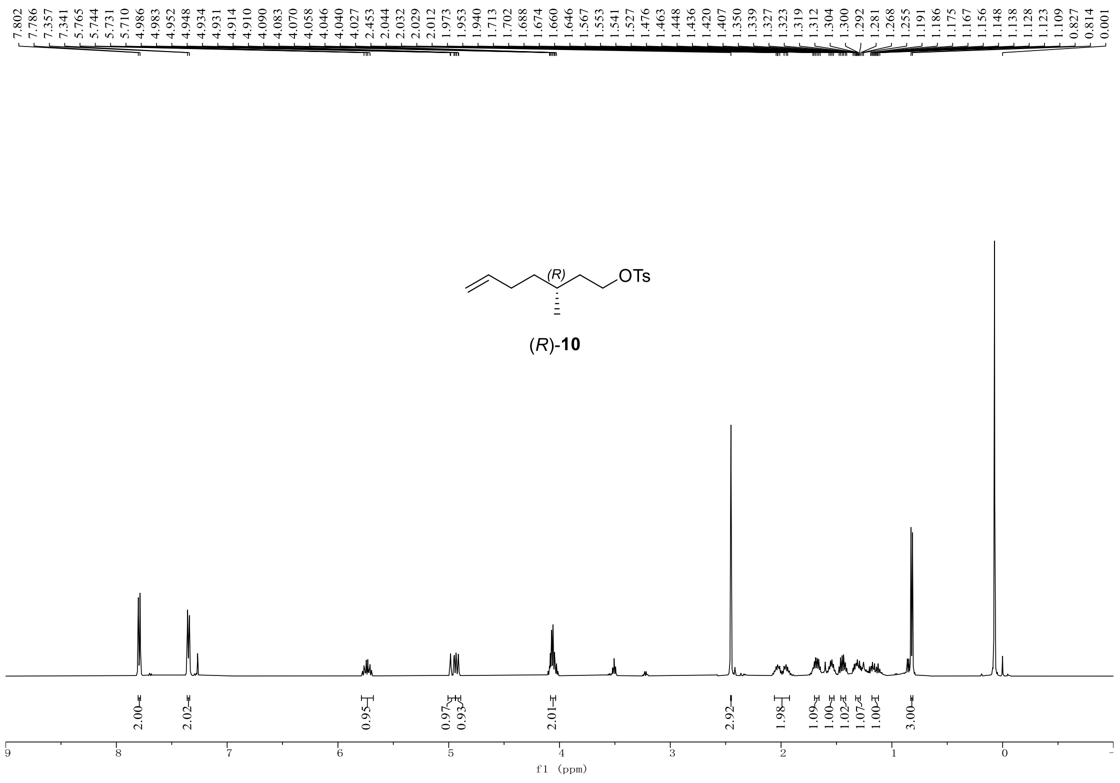

Figure S28: ^{13}C NMR Spectrum of (*R*)-3-methylhept-6-en-1-yl 4-methylbenzenesulfonate ((*R*)-**10**) (126 MHz, CDCl$_3$).

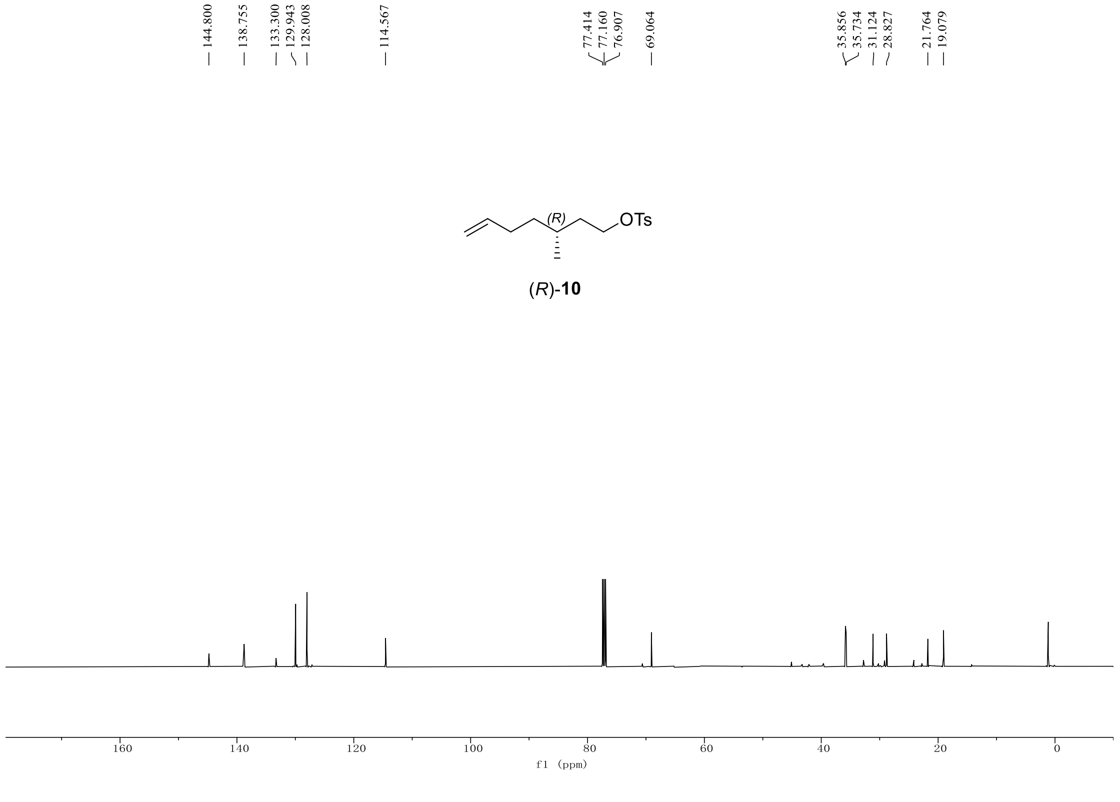

Figure S29: ^{1}H NMR Spectrum of (5*R*,9*R*)-5,9-dimethylundec-1-ene ((5*R*,9*R*)-**12**) (500 MHz, $CDCl_3$).

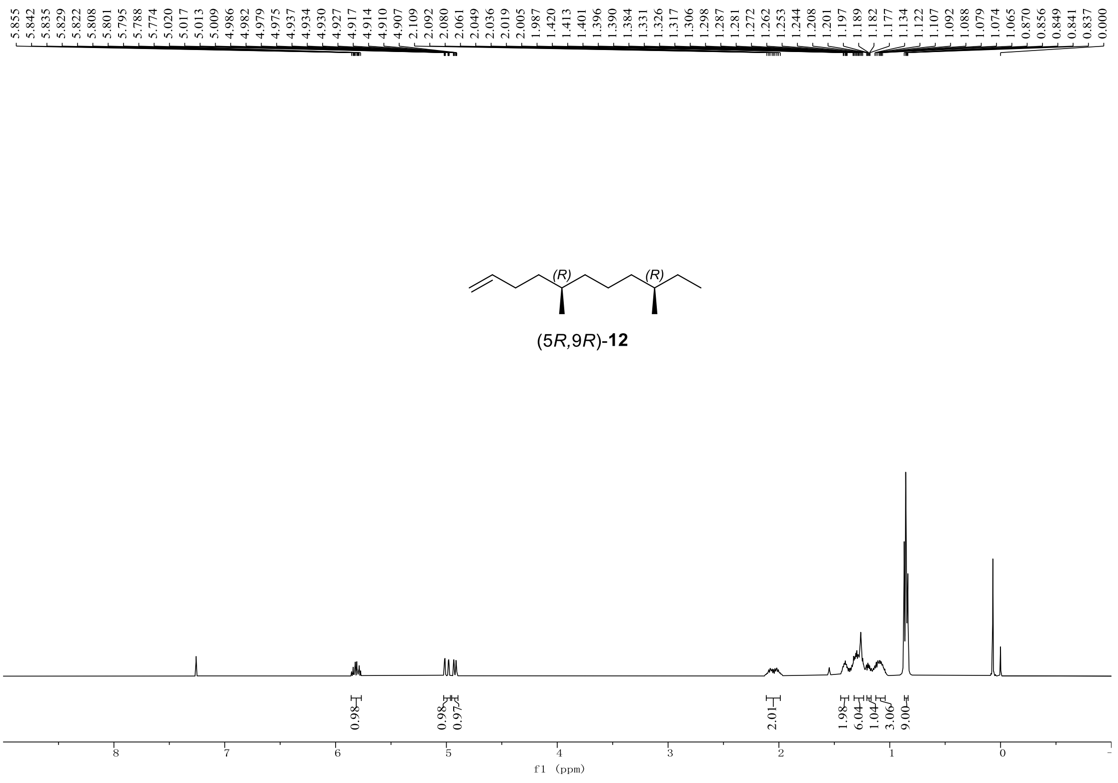

Figure S30: ^{13}C NMR Spectrum of (5*R*,9*R*)-5,9-dimethylundec-1-ene ((5*R*,9*R*)-**12**) (126 MHz, $CDCl_3$).

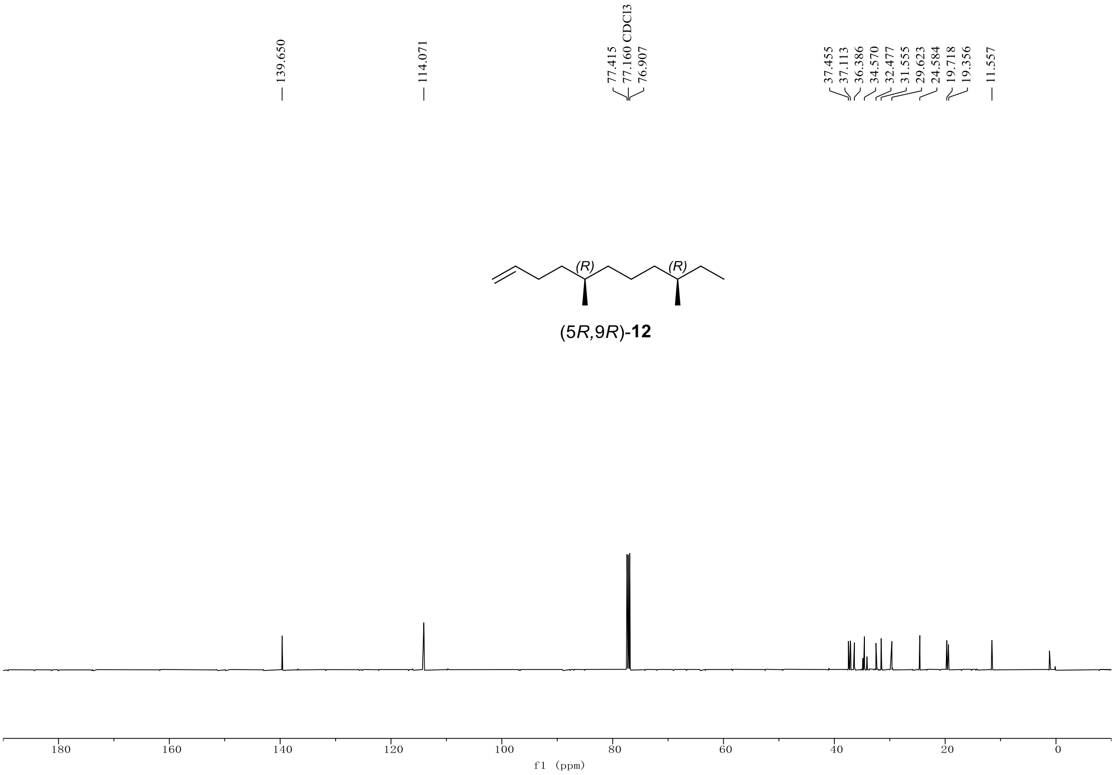

Figure S31: ^{1}H NMR Spectrum of (4*R*,8*R*)-4,8-dimethyldecanal ((4*R*,8*R*)-**1**) (500 MHz, CDCl$_3$).

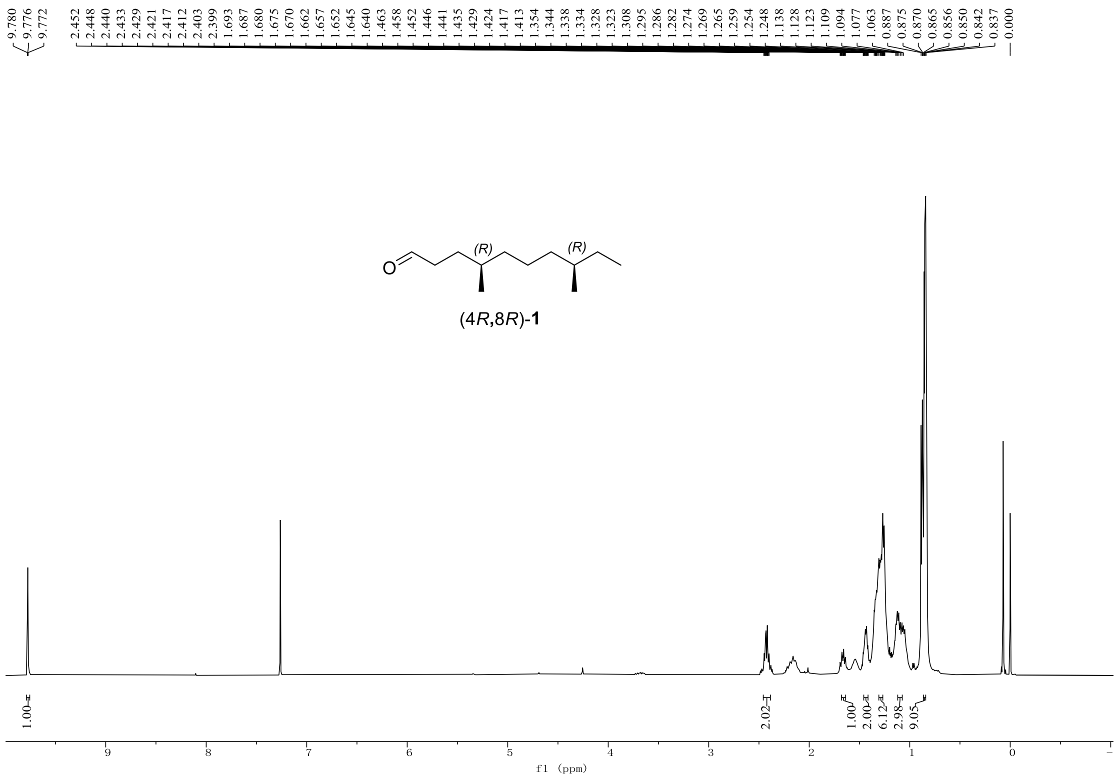

Figure S32: ^{13}C NMR Spectrum of (4*R*,8*R*)-4,8-dimethyldecanal ((4*R*,8*R*)-**1**) (126 MHz, CDCl$_3$).

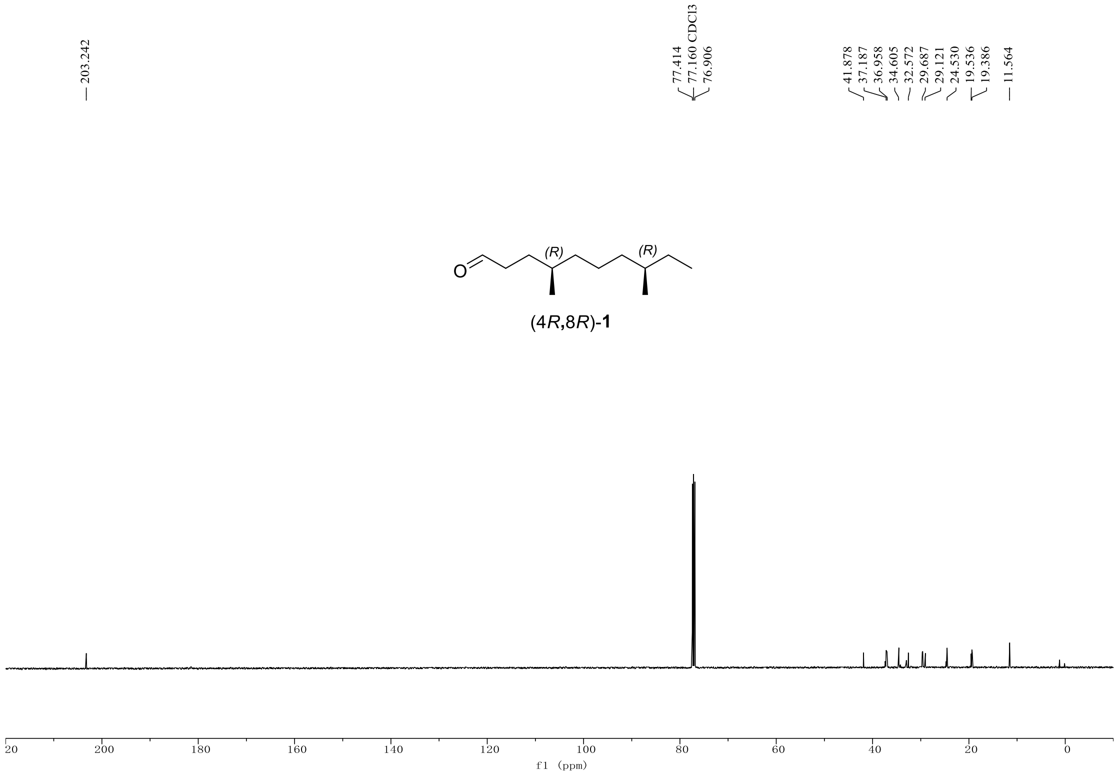

Figure S33: ^{1}H NMR Spectrum of (5*R*,9*S*)-5,9-dimethylundec-1-ene ((5*R*,9*S*)-**12**) (500 MHz, $CDCl_3$).

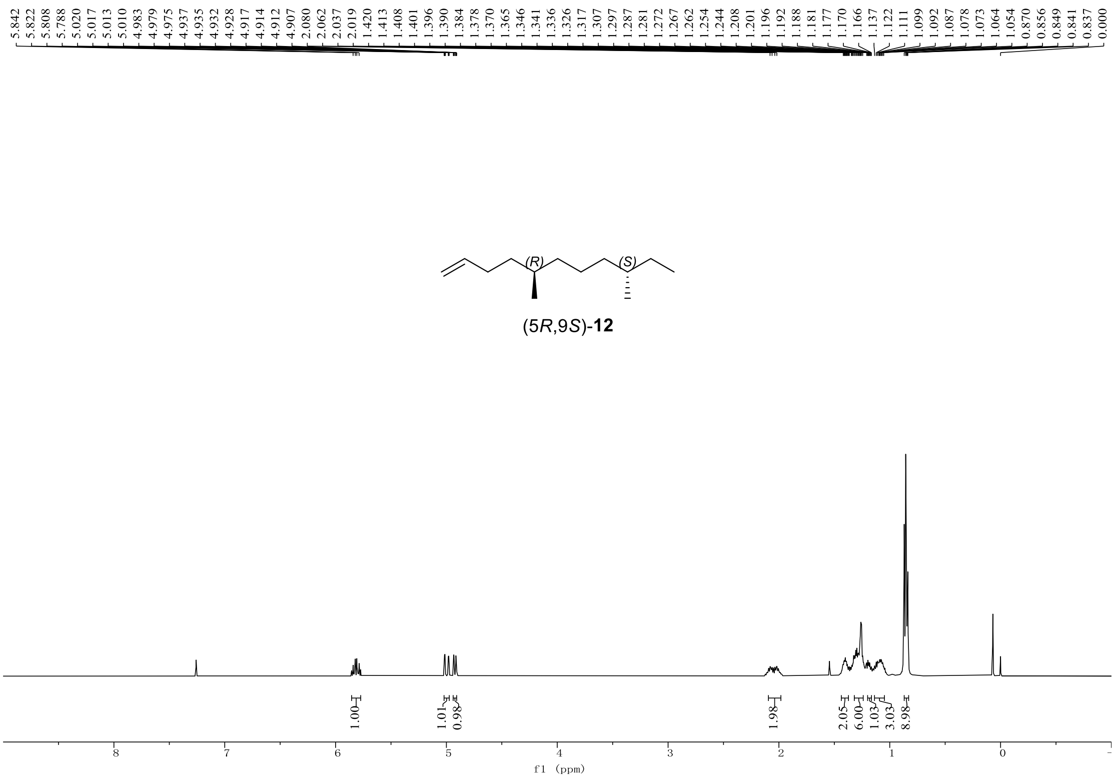

Figure S34: ^{13}C NMR Spectrum of (5*R*,9*S*)-5,9-dimethylundec-1-ene ((5*R*,9*S*)-**12**) (126 MHz, $CDCl_3$).

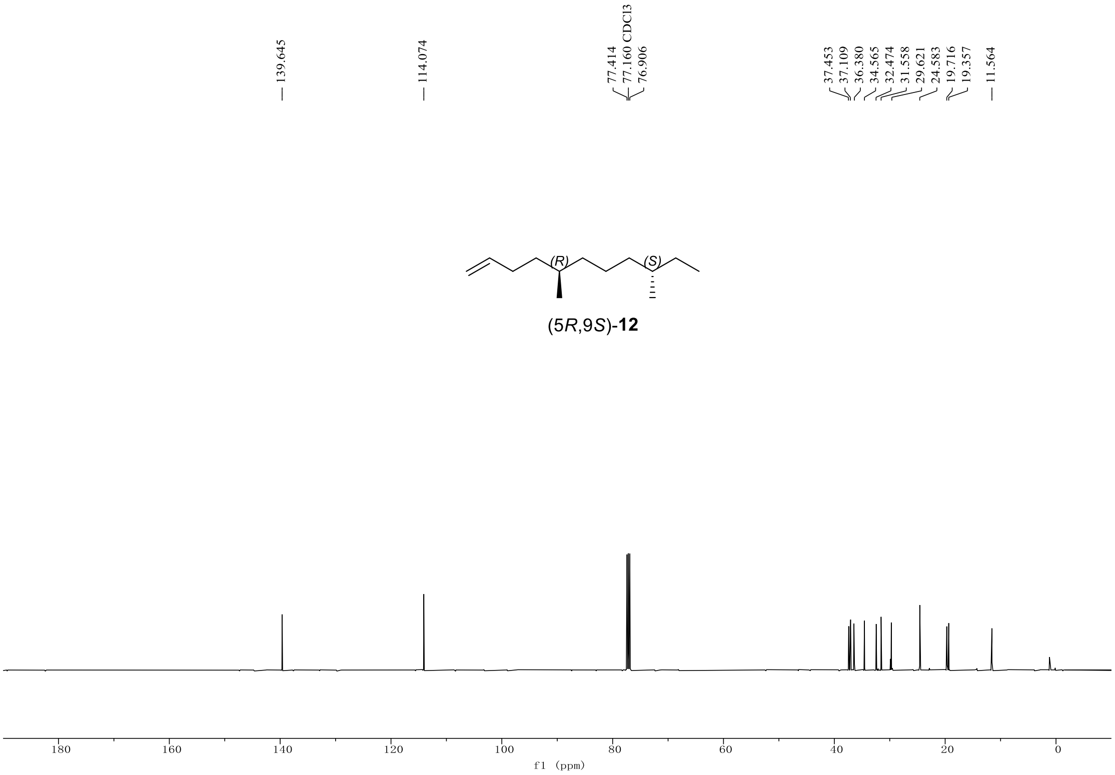

Figure S35: ^{1}H NMR Spectrum of (4*R*,8*S*)-4,8-dimethyldecanal ((4*R*,8*S*)-**1**) (500 MHz, CDCl$_3$).

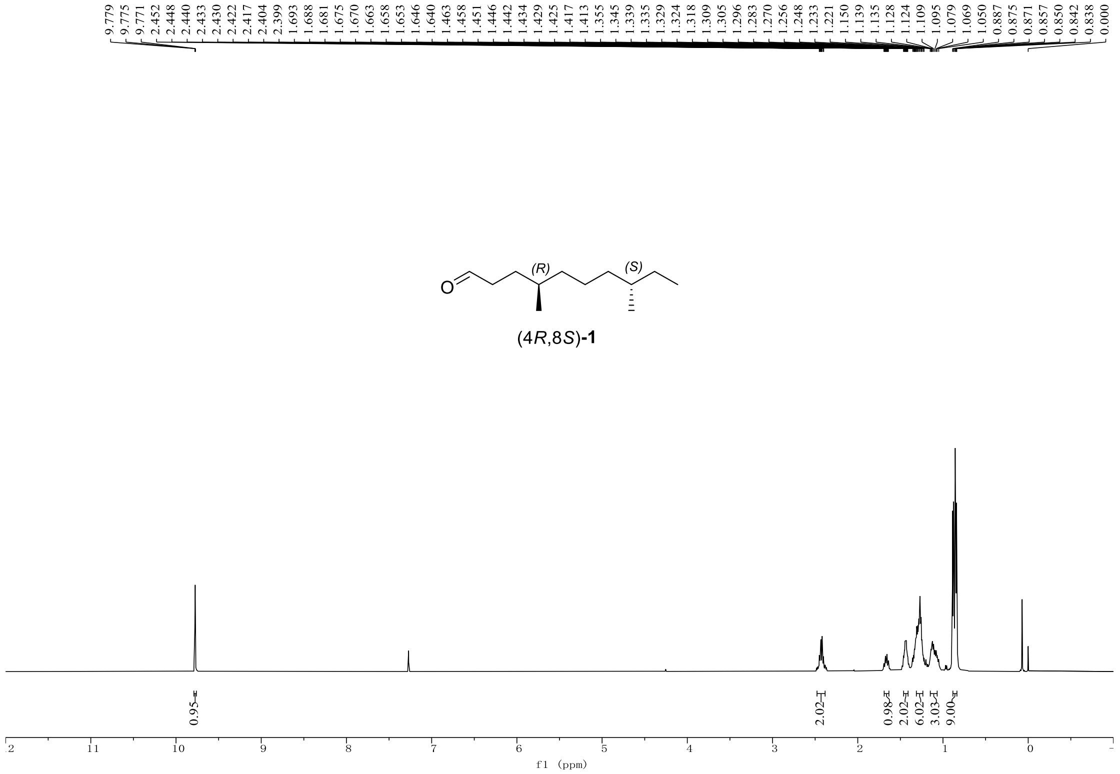

Figure S36: ^{13}C NMR Spectrum of (4*R*,8*S*)-4,8-dimethyldecanal ((4*R*,8*S*)-**1**) (126 MHz, CDCl$_3$).

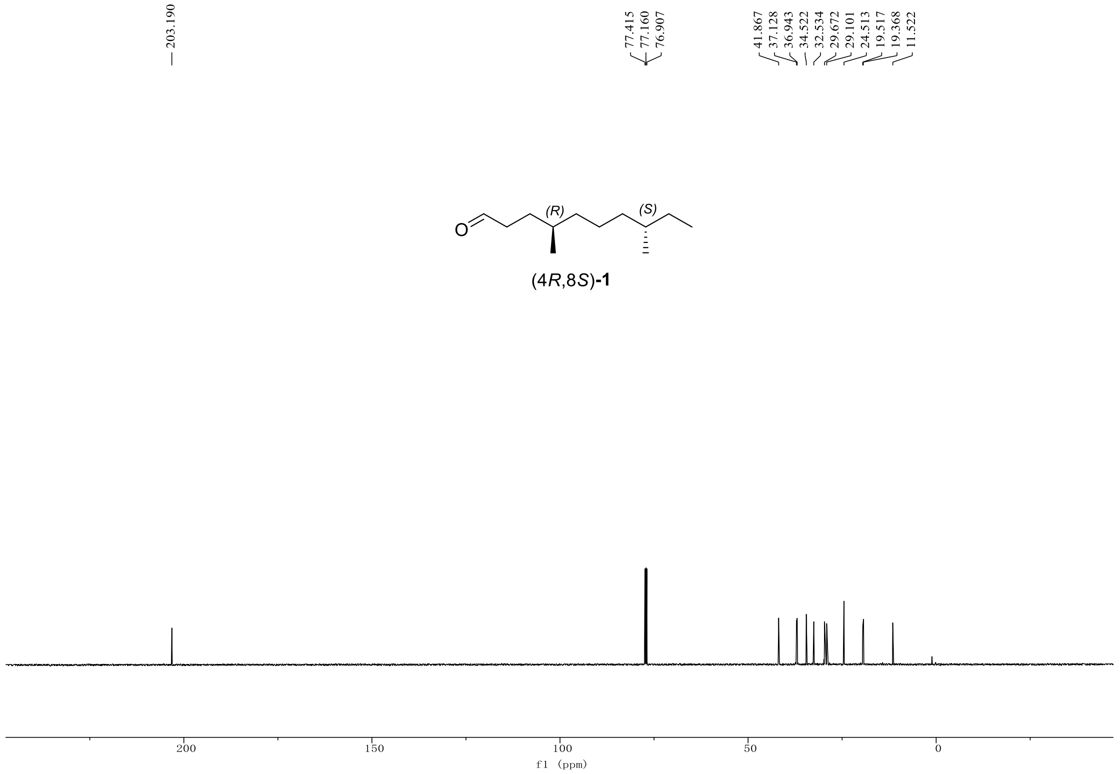

Figure S37: ^{1}H NMR Spectrum of (4*S*,8*R*)-4,8-dimethyldecanal ((4*S*,8*R*)-**1**) (500 MHz, CDCl$_3$).

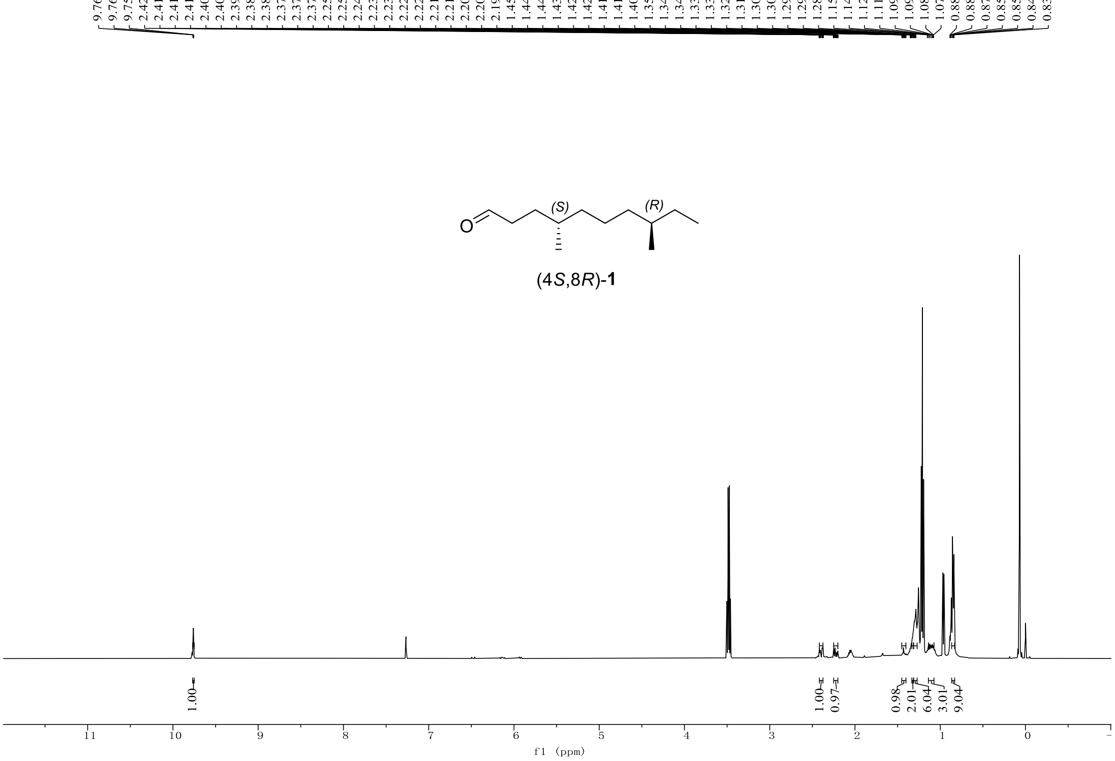

Figure S38: ^{13}C NMR Spectrum of (4*S*,8*R*)-4,8-dimethyldecanal ((4*S*,8*R*)-**1**) (126 MHz, CDCl$_3$).

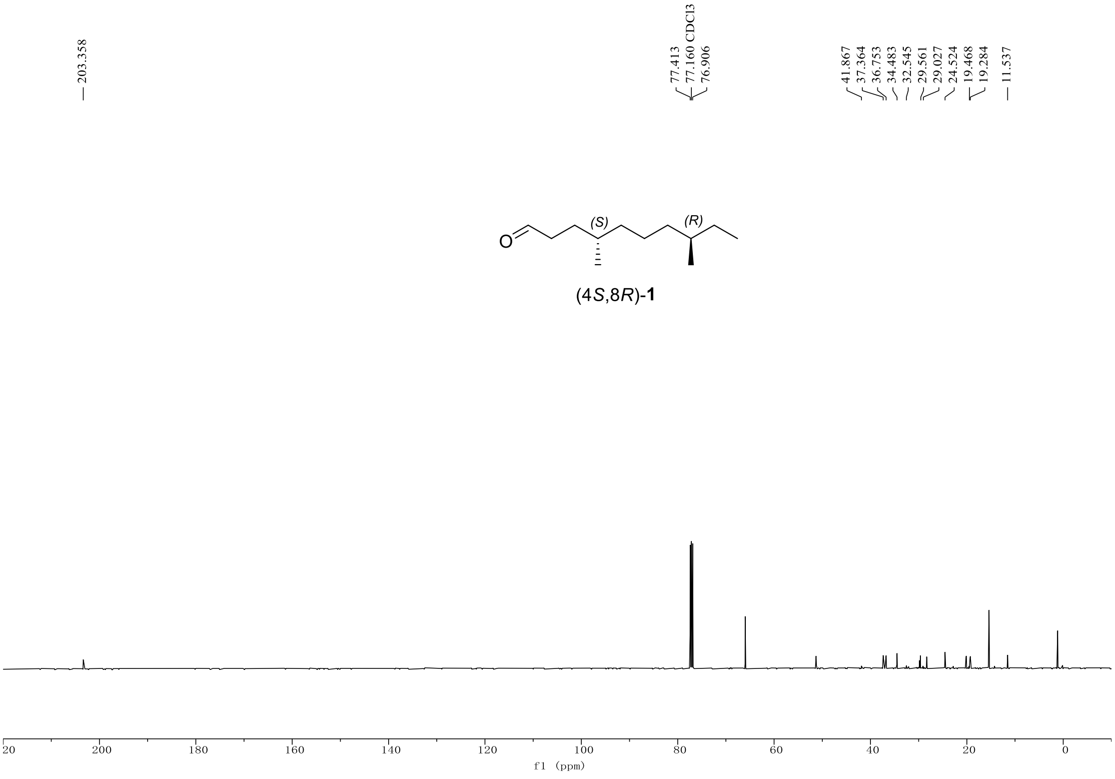

Figure S39: ^{1}H NMR Spectrum of (5*S*,9*S*)-5,9-dimethylundec-1-ene ((5*S*,9*S*)-**12**) (500 MHz, $CDCl_3$).

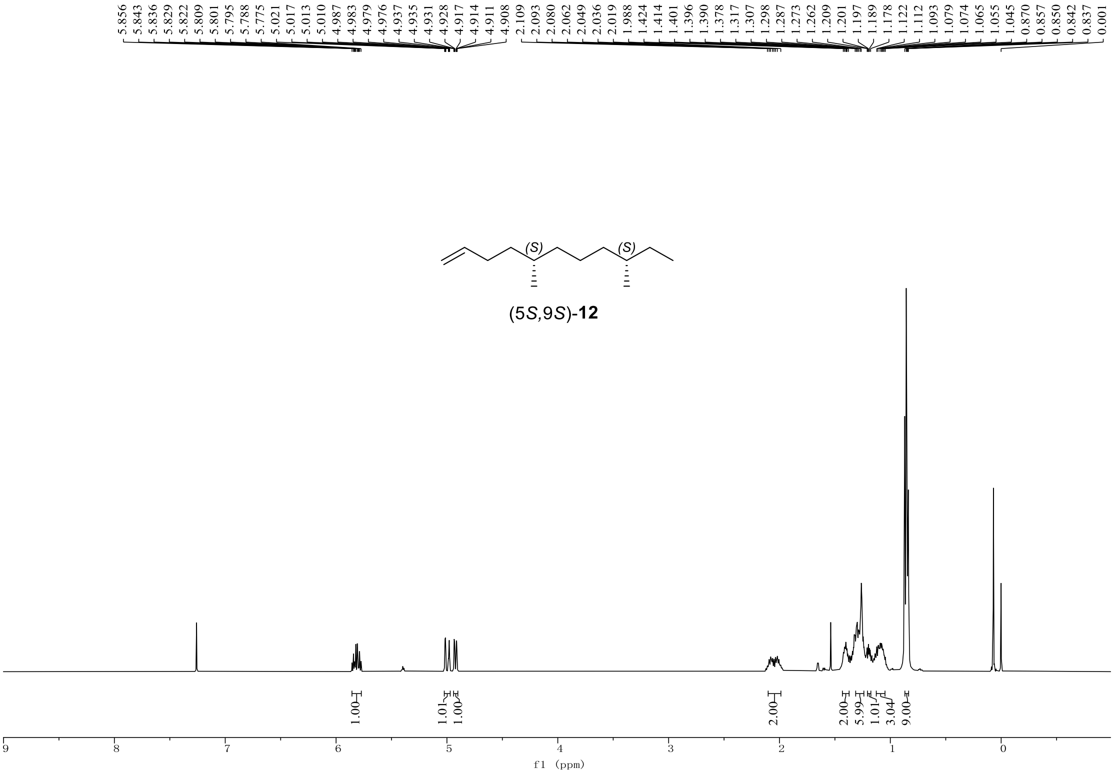

Figure S40: ^{13}C NMR Spectrum of (5*S*,9*S*)-5,9-dimethylundec-1-ene ((5*S*,9*S*)-**12**) (126 MHz, $CDCl_3$).

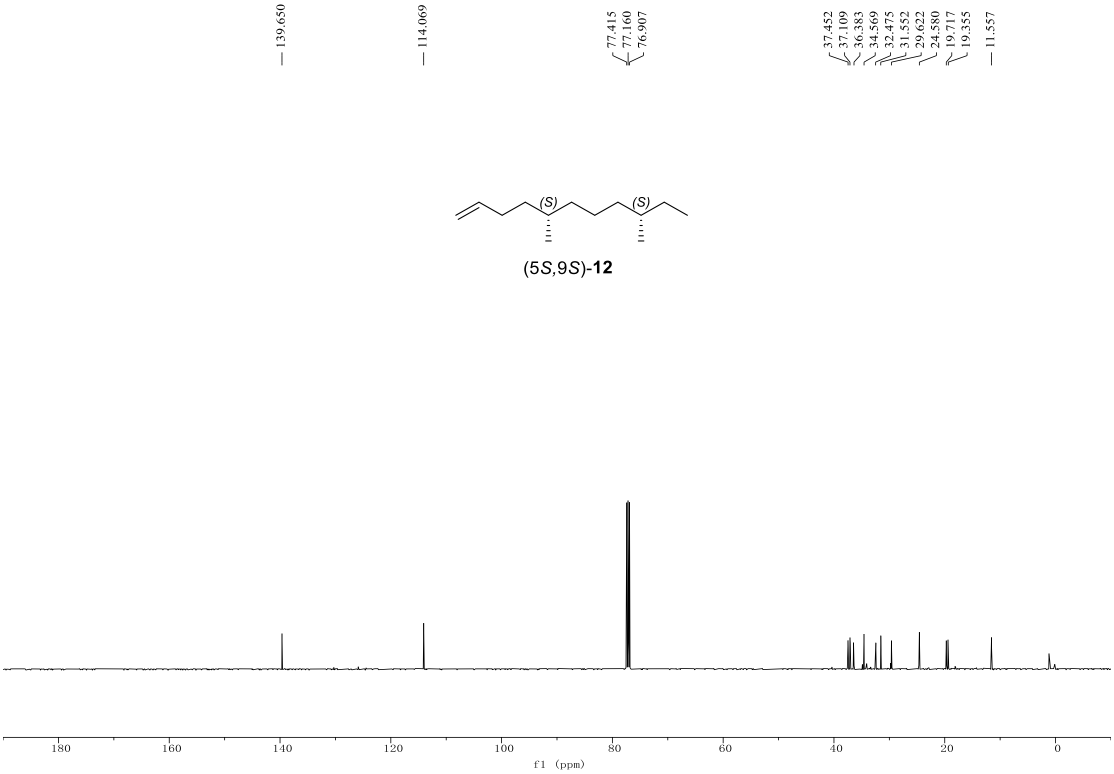

Figure S41: ^{1}H NMR Spectrum of (4*S*,8*S*)-4,8-dimethyldecanal ((4*S*,8*S*)-**1**) (500 MHz, $CDCl_3$).

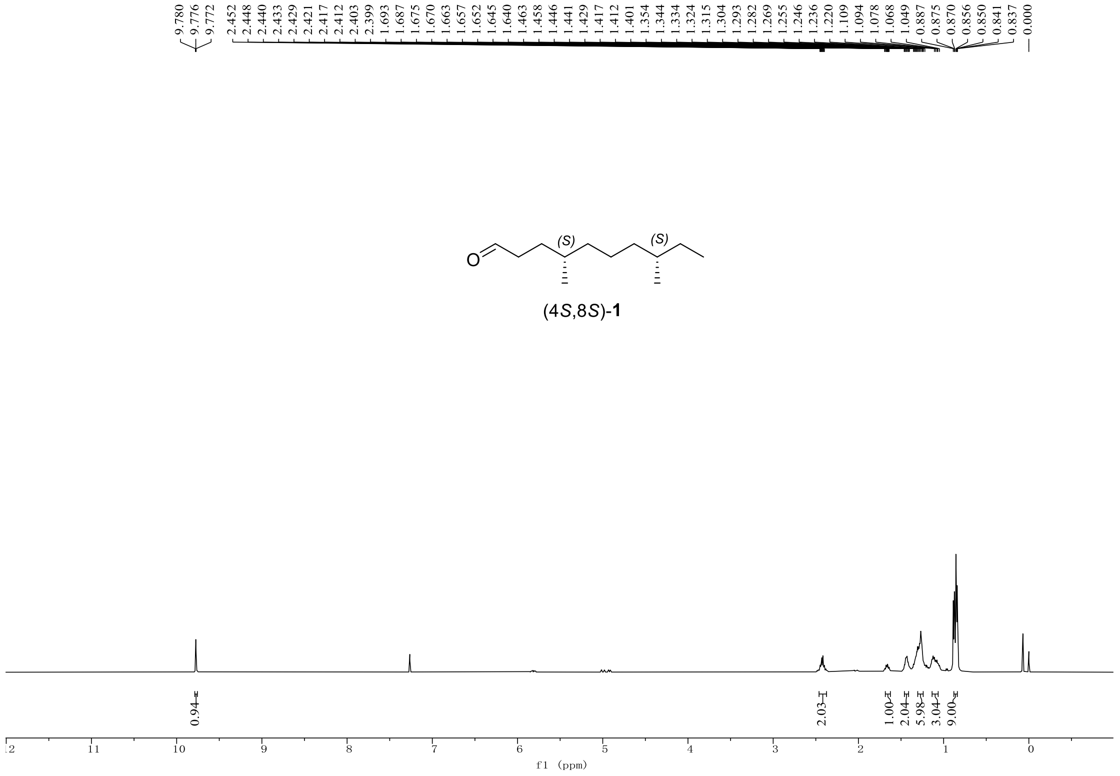

Figure S42: ^{13}C NMR Spectrum of (4*S*,8*S*)-4,8-dimethyldecanal ((4*S*,8*S*)-**1**) (126 MHz, $CDCl_3$).

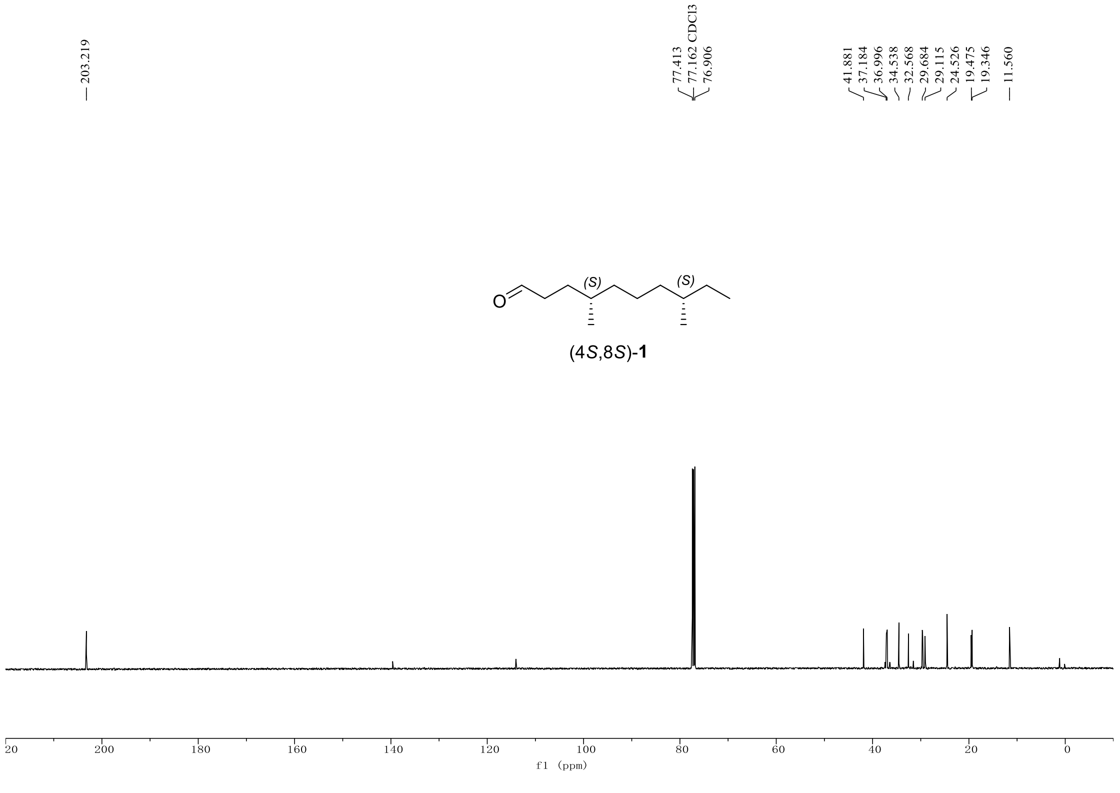

Figure S43: ^{1}H NMR Spectrum of hex-5-en-2-yl (2*R*)-3,3,3-trifluoro-2-methoxy-2-phenylpropanoate (**14**) (500 MHz, $CDCl_3$).

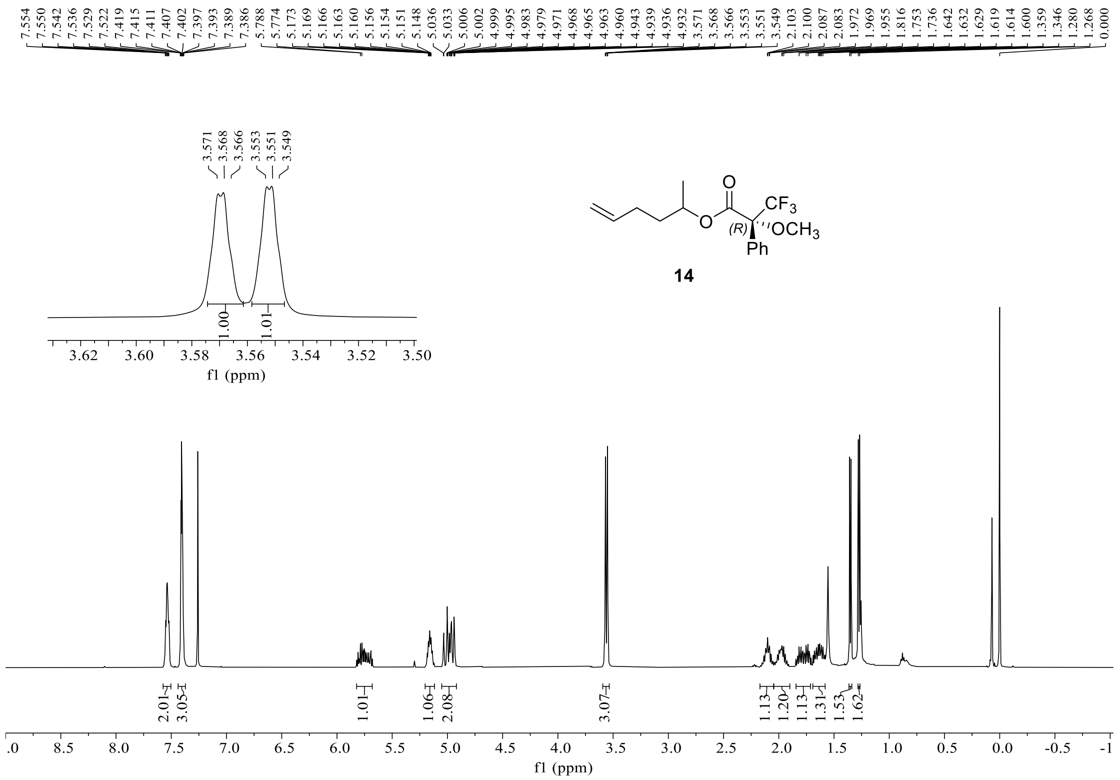

Figure S44: ^{13}C NMR Spectrum of hex-5-en-2-yl (2*R*)-3,3,3-trifluoro-2-methoxy-2-phenylpropanoate (**14**) (126 MHz, $CDCl_3$).

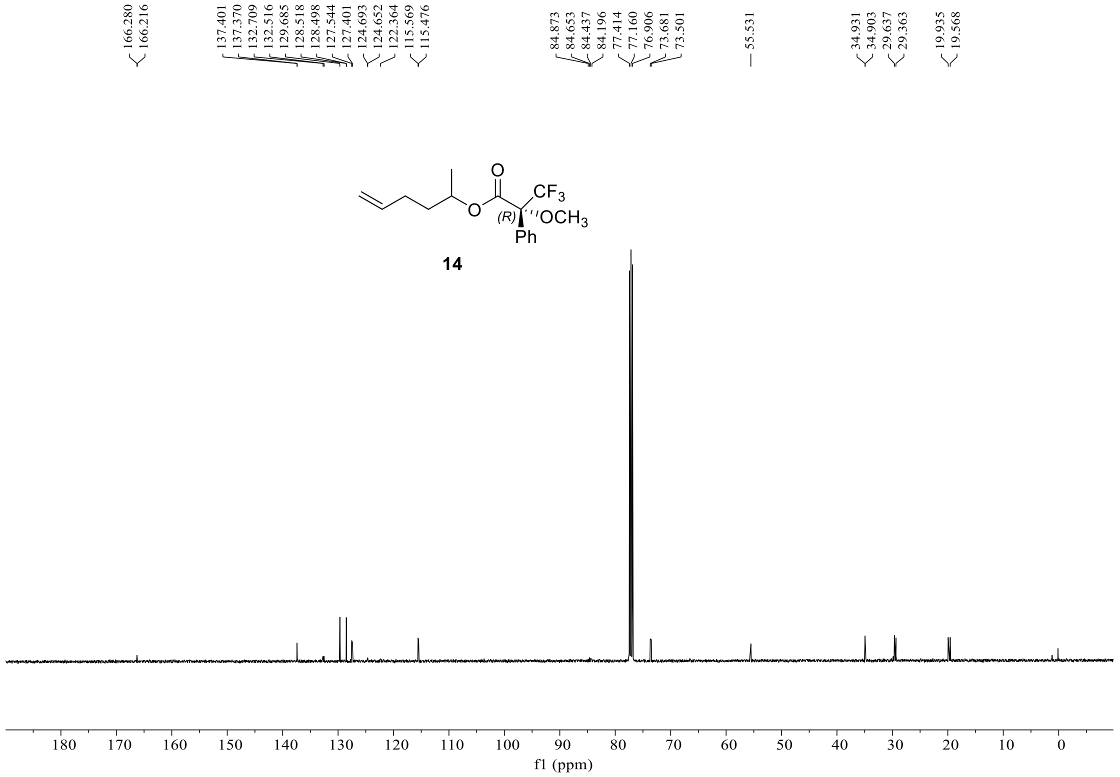

Figure S45: ^{19}F NMR Spectrum of hex-5-en-2-yl (2*R*)-3,3,3-trifluoro-2-methoxy-2-phenylpropanoate (**14**) (417 MHz, $CDCl_3$).

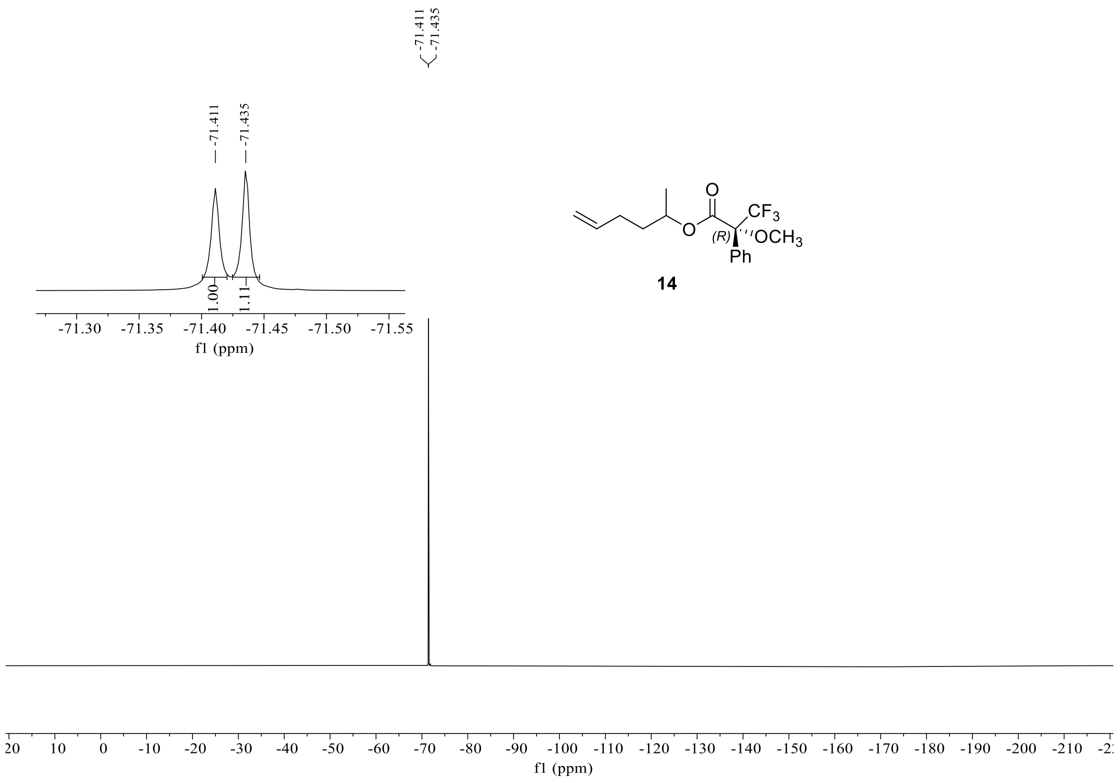

Figure S46: ^{1}H NMR Spectrum of (*S*)-hex-5-en-2-yl (*R*)-3,3,3-trifluoro-2-methoxy-2-phenylpropanoate (**15**) (500 MHz, $CDCl_3$).

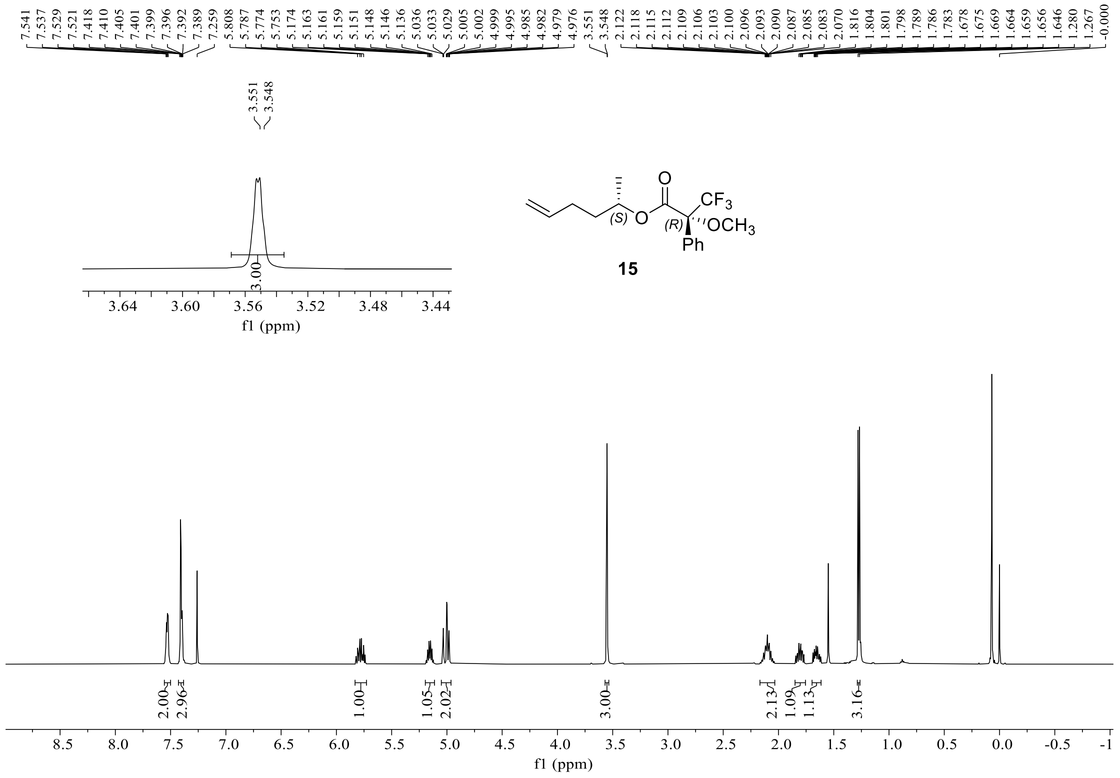

Figure S47: ^{13}C NMR Spectrum of (*S*)-hex-5-en-2-yl (*R*)-3,3,3-trifluoro-2-methoxy-2-phenylpropanoate (**15**) (126 MHz, $CDCl_3$).

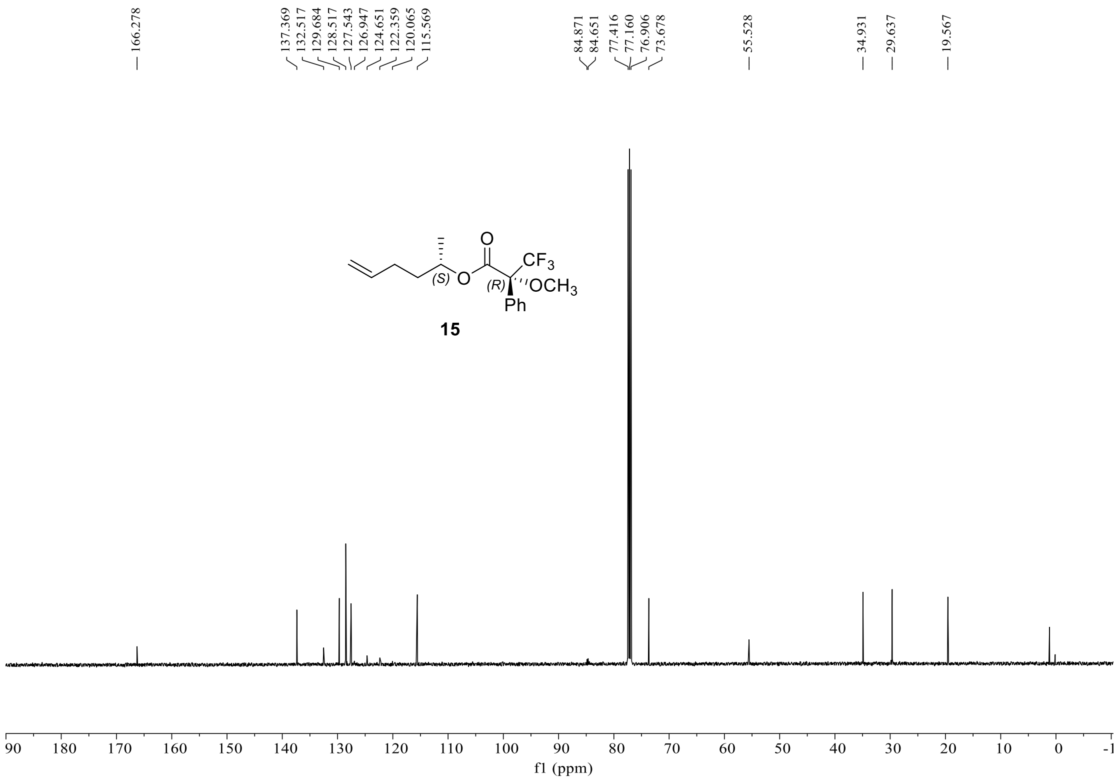

Figure S48: ^{19}F NMR Spectrum of (*S*)-hex-5-en-2-yl (*R*)-3,3,3-trifluoro-2-methoxy-2-phenylpropanoate (**15**) (417 MHz, $CDCl_3$).

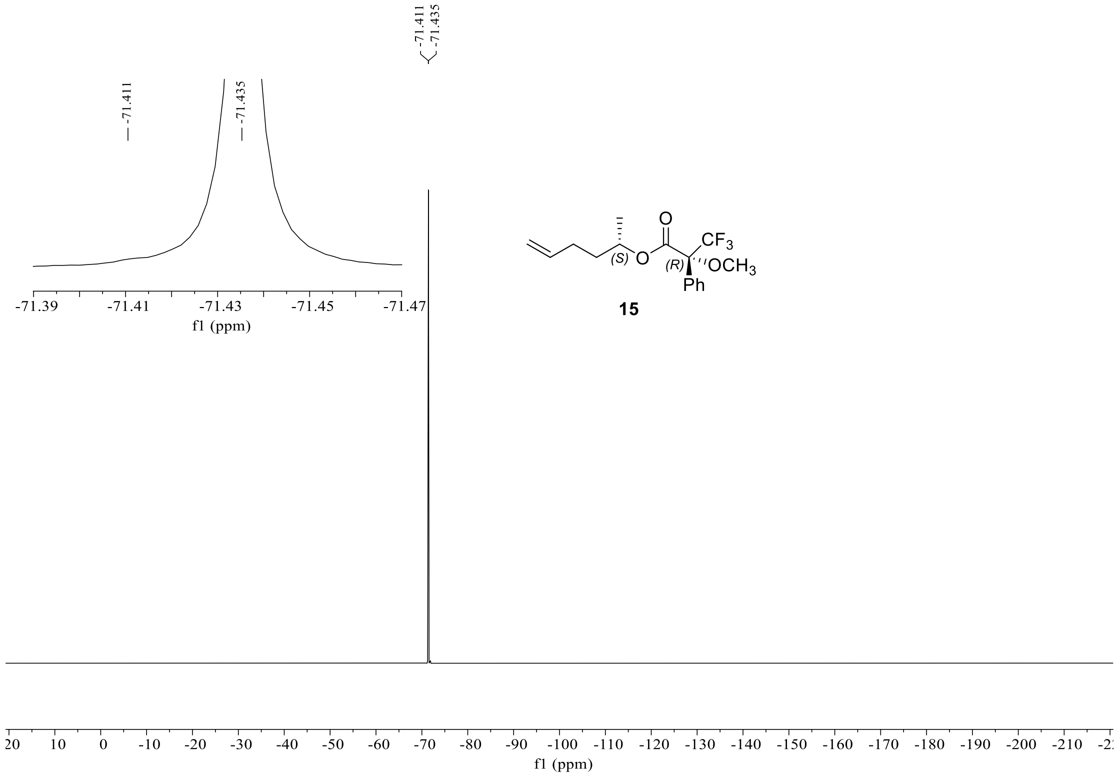

Figure S49: ^{1}H NMR Spectrum of (*R*)-hex-5-en-2-yl (*R*)-3,3,3-trifluoro-2-methoxy-2-phenylpropanoat (**16**) (500 MHz, $CDCl_3$).

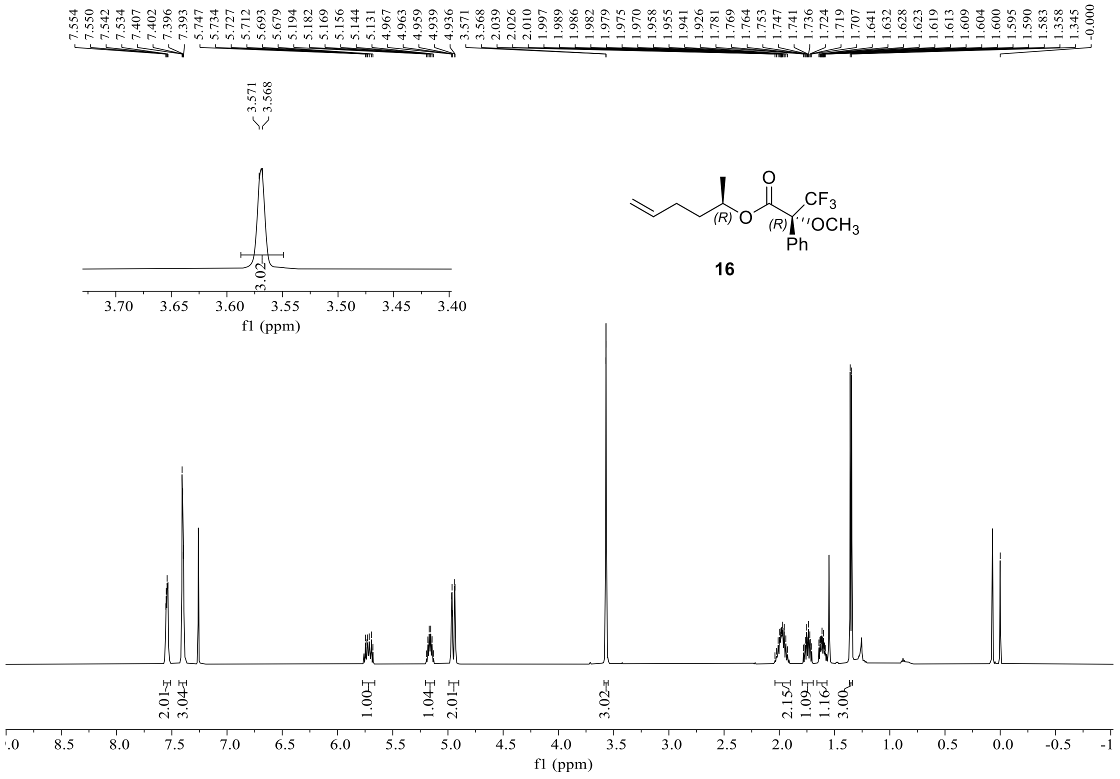

Figure S50: ^{13}C NMR Spectrum of (*R*)-hex-5-en-2-yl (*R*)-3,3,3-trifluoro-2-methoxy-2-phenylpropanoat (**16**) (126 MHz, $CDCl_3$).

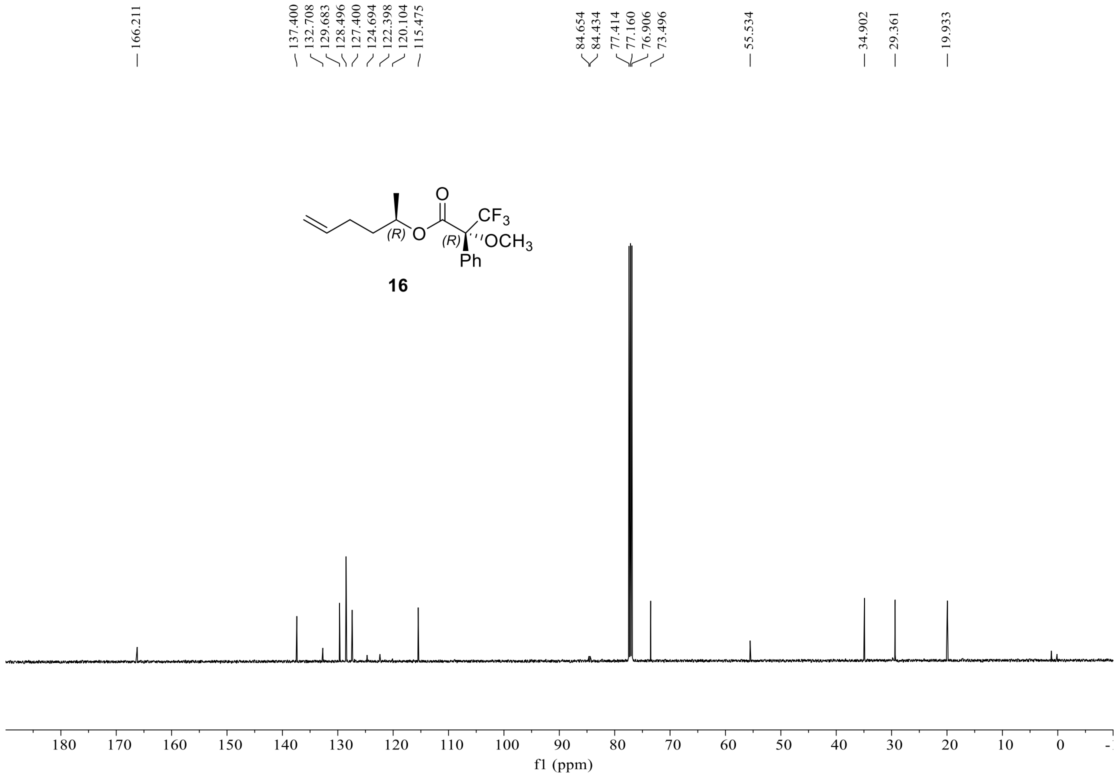

Figure S51: ^{19}F NMR Spectrum of (*R*)-hex-5-en-2-yl (*R*)-3,3,3-trifluoro-2-methoxy-2-phenylpropanoat (**16**) (417 MHz, $CDCl_3$).

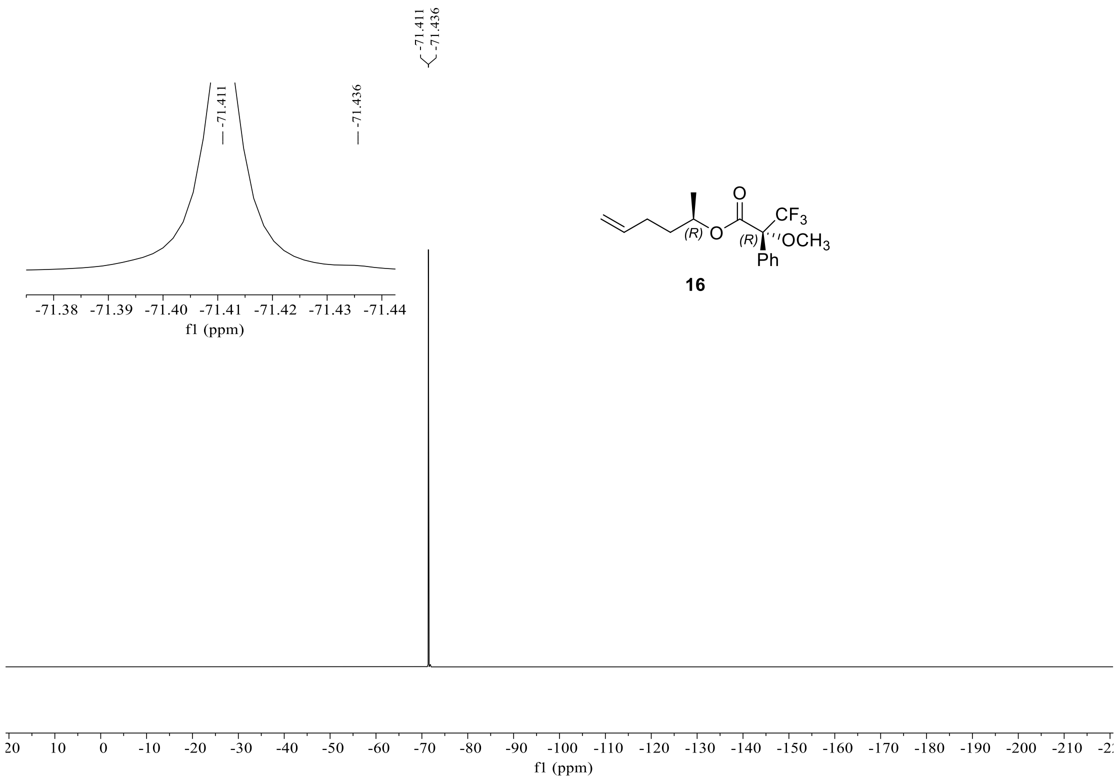

5. References

1. Thiraporn, A.; Iawsipo, P.; Tadpetch, K. *Synlett* **2022**, *33*, 1341-1346.
2. Nasufovic, V.; Kuellmer, F.; Bossneck, J.; Dahse, H.-M.; Gorls, H.; Bellstedt, P.; Stallforth, P.; Arndt, H.-D. *Chem. - Eur. J.* **2021**, *27*, 11633-11642.
3. Schaumann, E.; Kirschning, A.; Narjes, F. *J. Org. Chem.* **1991**, *56*, 717-723.
4. Taguri, T.; Yamakawa, R.; Fujii, T.; Muraki, Y.; Ando, T. *Tetrahedron Asymmetry* **2012**, *23*, 852-858.
5. Muraki, Y.; Taguri, T.; Yamakawa, R.; Ando, T. *J. Chem. Ecol.* **2014**, *40*, 250-258.
6. Von Doering, W.; Keliher, E. J. *J. Am. Chem. Soc.* **2007**, *129*, 2488-2495.
7. Baeckvall, J. E.; Andersson, P. G.; Vaagberg, J. O. *Tetrahedron Lett.* **1989**, *30*, 137-140.
8. a) Ramachandran, P. V.; Alawaed, A. A.; Hamann, H. J. *Org. Lett.* **2022**, *24*, 8481-8486. b) Mori, K. *Tetrahedron* **2009**, *65*, 2798-2805.
9. Zarbin, P. H. G.; Reckziegel, A.; Plass, E.; Borges, M.; Francke, W. *J. Chem. Ecol.* **2000**, *26*, 2737-2746.
10. Yang, D.; Zhang, C. *J. Org. Chem.* **2001**, *66*, 4814-4818.
11. Kameda, Y.; Nagano, H. *Tetrahedron* **2006**, *62*, 9751-9757.
12. Akhaev, N. S.; Zakladnoi, G. A.; Mavrov, M. V.; Moiseenkov, A. M.; Nguyen Cong, H.; Serebryakov, E. P.; Ceskis, B. *Bioorg. Khim.* **1988**, *14*, 243.
13. Akasaka, K.; Tamogami, S.; Beeman, R. W.; Mori, K. *Tetrahedron* **2011**, *67*, 201-209.
14. Ohtani, I.; Kusumi, T.; Kashman, Y.; Kakisawa, H. *J. Am. Chem. Soc.* **1991**, *113*, 4092-4096.
15. Dale, J. A.; Dull, D. L.; Mosher, H. S. *J. Org. Chem.* **1969**, *34*, 2543-2549.
